# Depression and risk of arthritis: A Mendelian randomization study

**DOI:** 10.1002/brb3.3551

**Published:** 2024-06-07

**Authors:** Min‐Yi Wu, Yu‐Ying Liang, Qi‐Jia Han, Zhu Ai, Hao‐Wen Yan, Zhi‐Ming Xiang

**Affiliations:** ^1^ Department of Radiology Guangzhou Panyu Central Hospital Guangzhou China

**Keywords:** arthritis, depressive, Mendelian randomization

## Abstract

**Introduction:**

Observational studies have found that most patients with arthritis have depression. We aimed to determine the causal relationship between various types of arthritis and depression.

**Methods:**

We conducted a two‐sample bidirectional Mendelian randomized (MR) analysis to determine whether there was a significant causal relationship between depression and multiple types of arthritis. The data of our study were derived from the publicly released genome‐wide association studies (GWASs) and the largest GWAS meta‐analysis. MR analysis mainly used inverse‐variance weighted method; supplementary methods included weighted median, weighted mode, and MR‐Egger using MR pleiotropy residual sum and outlier to detect and correct for the presence of pleiotropy.

**Results:**

After adjusting for heterogeneity and horizontal pleiotropy, we found that depression was associated with an increased risk of osteoarthritis (OA) (OR = 1.02, 95%CI: 1.01–1.02, *p* = 2.96 × E − 5). In the reverse analysis, OA was also found to increase the risk of depression (OR = 1.10, 95%CI: 1.04–1.15, *p* = .0002). Depression only increased the risk of knee OA (KOA) (OR = 1.25, 95%CI: 1.10–1.42, *p* = 6.46 × E − 4). Depression could potentially increase the risk of spondyloarthritis (OR = 1.52, 95%CI: 1.19–1.94, *p* ≤ 8.94 × E − 4).

**Conclusion:**

There is a bidirectional causal relationship of depression with OA. However, depression only augments the risk of developing KOA. Depression may increase the risk of spondyloarthritis and gout.

## Graphical abstract

This study investigated A two‐sample two‐way Mendelian randomization (MR) analysis was performed to determine whether there is a relationship between depression and multiple types of arthritis. The present study found a bidirectional causal relationship between depression and OA. The depression only increased the risk of KOA.

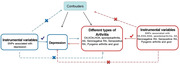

## 1 INTRODUCTION

Globally, arthritis serves as a disabling ubiquitous disease, impacting approximately 250 million individuals. Alarmingly, this condition represents one of the most rapidly proliferating health challenges worldwide (Cross et al., 2014; Theo et al., 2020). It is also a major cause of joint dysfunction, disability, and high medical costs. Arthritis is a general term for various types of joint inflammatory diseases. Classified by etiology, it can be divided into (1) degenerative joint disease, (2) idiopathic inflammatory diseases, (3) crystal‐induced arthritis, and (4) infectious arthritis. Many studies have found a high co‐occurrence of depression and arthritis. In a study of adults and children by Hanns et al. (2018), 14.7% of 102 children with juvenile idiopathic arthritis also had major depression. However, the causal relationship between depression and arthritis requires further investigation due to the limitations of cross‐sectional studies.

Mendelian randomization (MR) employs genetic variation and single‐nucleotide polymorphisms (SNPs) to infer causality between exposure and disease outcomes (Richmond & Davey Smith, 2022). MR provides a concise and robust approach to assess the relationship between risk factors and disease prognosis (Burgess et al., 2020). This analysis employs SNPs linked to exposure‐related genes as instrumental variables (IVs), under specific assumptions, to estimate the causal effect of exposure on outcomes (Skrivankova et al., 2021). Two‐sample MR analysis uses data from two independent genome‐wide association study (GWAS) to identify potential exposures and outcomes. Eliminating confounders enhances the reliability of MR analysis results (Hartwig et al., 2016). To the best of our knowledge, this is the inaugural two‐sample MR study investigating the causal association between arthritis and depression. In this study, MR analysis was performed on two‐sample GWAS data to evaluate the causal relationship among different types of arthritis and depression.

## 2 MATERIALS AND METHODS

### 2.1 Research and data sources

**FIGURE 1:**
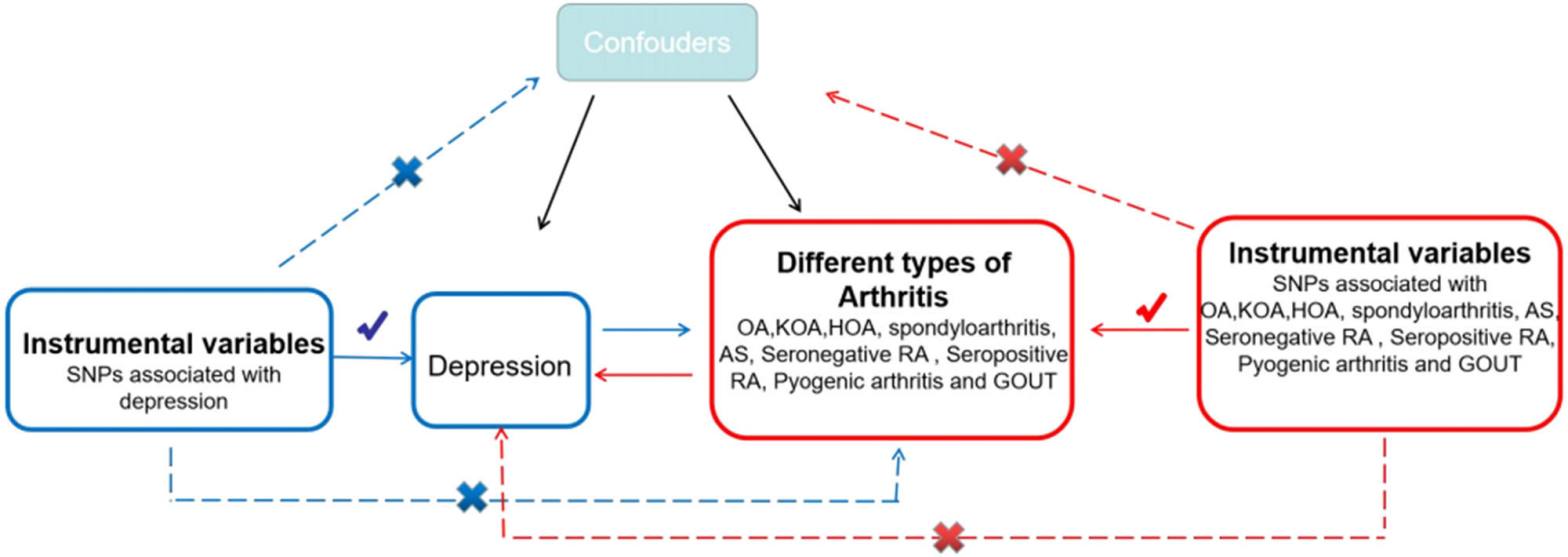
Flow chart of the Mendelian randomized (MR) study.

In MR research, the IV must meet three fundamental assumptions: (1) Genetic variation ought to be significantly relevant to exposure; (2) genetic variation ought to be linked to exposure, not linked to any confounding factors associated with the outcome; (3) genetic variation should have nothing to do with exposure or confounding factor‐dependent outcome. It is difficult to estimate causality without making any of the above assumptions (Lawlor et al., 2008). We used data from published research and publicly available GWAS statistics. An overview of the MR framework is illustrated in Figure 1.

We used genetic instruments for depression from Howard et al.’s (2019) comprehensive GWAS meta‐analysis of 807,553 individuals, including 246,363 depression cases and 561,190 controls, mainly of European ancestry, from the UK Biobank study, 23andMe, and Major Depressive Disorder Working Group of the Psychiatric Genomics Consortium (PGC) (Howard et al., 2019). We obtained a total of 98 SNPs associated with depression. In the reverse study, depression‐associated genes were derived from a European GWAS meta‐analysis of 9851,867 individuals, including 26,595 cases and 436,338 controls, from the MRC‐IEU consortium. Which database collects self‐reported depression information for non‐cancer disease codes? In this study, we focused on the following clinically common types of arthritis: (1) osteoarthritis (OA), knee OA (KOA), and hip OA (HOA) within degenerative arthritis; (2) spondylitis, ankylosing spondylitis (AS), seronegative RA, and seropositive RA in idiopathic inflammatory diseases; (3) infectious arthritis of suppurative arthritis; and (4) gout in crystalline arthritis. The genetic IVs for different types of arthritis were obtained from published research or published GWAS. Diagnosis of KOA, HOA, RA, SA, and so on is based on self‐reported clinical evaluation, laboratory evaluation, and histopathology confirmed. The details of the dataset are listed in Table S1. To mitigate pleiotropy bias (Burgess et al., 2019), all subjects were of European ancestry (males and females).

### 2.2 Selection of instrumental variables

To ensure research integrity and accuracy, the IV estimation was conducted with the following criteria: first, significant SNP‐exposure relation (*p* < 5 × E − 8 or *p* < 5 × E − 6) (Zhu et al., 2018); second, no linkage disequilibrium (LD) aggregation among IVs (*r* ^2^ < .001, distance >10,000 kb); third, the exclusion of all palindrome SNPs to align the effect of SNPs on exposure with the effect on results. In our study, the PhenoScanner database was used to exclude pleiotropic SNPs linked to exposure outcomes (Kamat et al., 2019). The correlation between IV estimation and exposure was measured using the *F*‐statistic, with values greater than 10 (Deng et al., 2020), indicating strong correlation.

### 2.3 Statistical analysis

All SNPs were harmonized between the exposure and the outcome using allele alignment to ensure consistent effect estimation. The primary analysis employed the inverse‐variance weighted (IVW) method (Burgess et al., 2019), the weighted median, MR‐Egger, weighted mode, and simple mode as other analysis methods (Bowden et al., 2015; Burgess et al., 2017; Rees et al., 2017). To ensure the reliability of our results, we used Cochran's *Q* analysis in IVW to assess heterogeneity. If *p* > .05 indicates no heterogeneity (Minică et al., 2018), we considered the fixed‐effect IVW method as the primary approach (Bowden et al., 2017; Lin et al., 2021). If *p* < .05 indicates heterogeneity, we prioritized using the multiplicative‐random IVW method. We utilized the MR‐Egger intercept test to identify directional pleiotropy (Bowden et al., 2015). To conduct sensitivity analyses, we employed a leave‐one‐out test to examine the influence of individual SNPs on the overall estimates. Additionally, we assessed pleiotropy using MR pleiotropy residual sum and outlier (MR‐PRESSO) and reevaluated effect estimates after excluding outliers (Karageorgiou et al., 2023; Verbanck et al., 2018). All statistical data analyses were carried out using R software version 4.1.3.v, employing the “TwoSampleMR” and “MR‐PRESSO” packages for this study (Hemani et al., 2018; Verbanck et al., 2018).

## 3 RESULTS

### 3.1 Causal relationship between depression and different types of arthritis

We used IV estimation by selecting depression SNPs with a significant association to the exposure (*p* < 5 × E − 8) and removed outliers using the MR‐PRESSO global outlier test. Our study identified 89 highly correlated SNPs for depression, including OA, KOA, HOA, spondyloarthritis, seronegative RA, seropositive RA, pyogenic arthritis, and gout. Additionally, AS showed 58 strongly associated SNPs for depression (Table S2).

**FIGURE 2:**
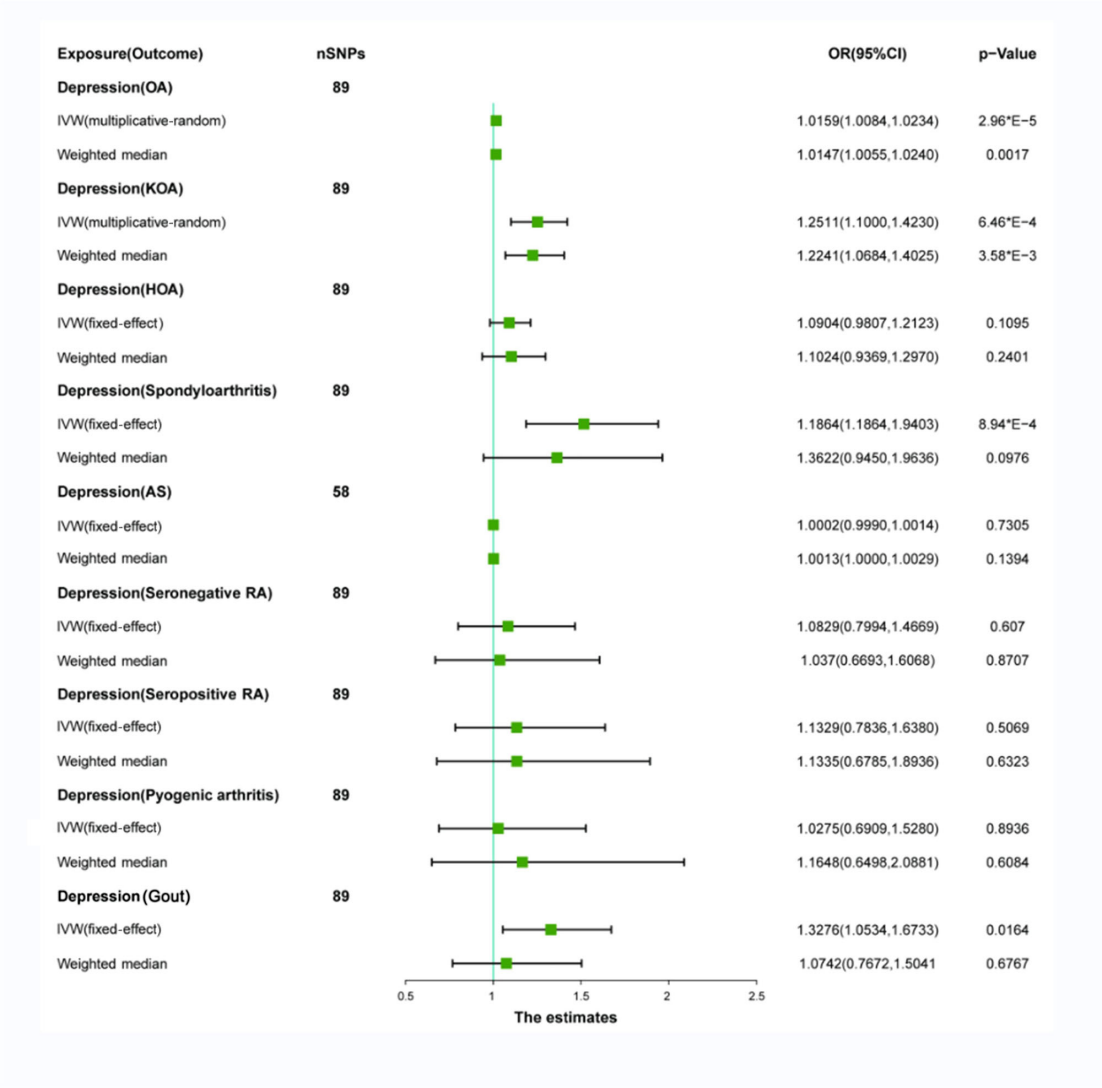
Causal relationship between depression and different types of arthritis.

Significant heterogeneity was found between OA and KOA (OA = 0.0029; KOA = 4.09 × 10^−6^) using Cochran's *Q* test with the multiplicative‐random IVW method. No significant heterogeneity was observed in HOA, spondyloarthritis, seronegative RA, seropositive RA, pyogenic arthritis, and gout, where the fixed‐effect IVW method was applied for analyses (Table 1). After excluding pleiotropic SNPs, we observed a significant positive association between depression and OA (OR: 1.0159; 95%CI: 1.0084–1.0234; *p* = 2.9 × E − 5). The weighted median model also revealed a similar positive association between depression and OA (OR: 1.0147; 95%CI: 1.0055–1.0240; *p* = 1.71 × E − 3). Depression showed a significant positive association with KOA using both the IVW method (OR: 1.2511; 95%CI: 1.1000–1.4230; *p* = 6.47 × E − 4) and the weighted median model (OR: 1.2241; 95%CI: 1.0684–1.4025; *p* = 3.58 × E − 3). Similarly, there was a positive correlation between depression and spondyloarthritis (OR: 1.5172; 95%CI: 1.1864–1.9403; *p* = 8.94 × E − 4), depression and gout (OR: 1.3276; 95%CI: 1.0534, 1.6733; *p* = .0164), but no causal relationship was found when using the weighted median model (OR: 1.3622; CI: 0.9450–1.9636, *p* = .0976) and (OR: 1.0742; 95%CI: .7672, 1.5041; *p* = .6767) (Figure 2). No significant horizontal pleiotropy was found in the sensitivity analysis, with MR‐Egger regression intercepts close to zero (Table 1). The leave‐one‐out analysis, forest plots, scatter plots, and funnel plots of MR are shown in Figures S1, S2, and S4. No causal relationship was found between depression and HOA, spondyloarthritis, AS, seronegative RA, seropositive RA, pyogenic arthritis, and gout in the MR analysis (Figure 2). Their MR leaky‐one‐out analysis, forest plots, scatter plots, and funnel plots are shown in Figures S3 and S5–S9.

**TABLE 1:** Heterogeneity and pleiotropy analysis of depression with different types of arthritis.

			Pleiotropy test		Heterogeneity test		MR‐PRESSO	
Exposure	Outcome	nSNPs	Beta (SE)	p‐Value	Cochran's *Q*	p‐Value	RSSOBs	*p*‐Value of global test
Depression	OA	89	.0006	.9222	128.9211	.0029	149.4082	.004
	KOA	89	.0095	.2911	156.1715	4.09E‐06	221.0826	<.001
	HOA	89	.0086	.3383	101.2585	.1580	131.8326	.022
	Spondyloarthritis	89	.0195	.4455	83.2207	.6242	94.6034	.7
	AS	58	.0001	.8176	54.3796	.5740	70.5547	.4
	Seronegative RA	89	.0240	.7409	67.2135	.9515	81.6953	.924
	Seropositive RA	89	.0293	.4672	78.2555	.7620	92.2606	.734
	Pyogenic arthritis	89	.0314	.9949	68.4624	.9391	75.6042	.962
	Gout	89	.0193	.1178	100.1062	.1779	123.2853	.082

Abbreviations: AS, ankylosing spondylitis; HOA, hip osteoarthritis; KOA, knee osteoarthritis; MR‐PRESSO, MR pleiotropy residual sum and outlier; OA, osteoarthritis; seronegative RA, seronegative rheumatoid arthritis; seropositive RA, seropositive rheumatoid arthritis; SNP, single‐nucleotide polymorphism.

### 3.2 Causal relationship between different types of arthritis and depression

We selected SNPs associated with arthritis, including OA, KOA, HOA, spondyloarthritis, AS, seronegative RA, seropositive RA, pyogenic arthritis, and gout (*p* < 5 × E − 6) as IVs. LD tests and confounder removal were performed. Finally, we obtained depression‐related SNPs: 33 (OA), 78 (KOA), 59 (HOA), 17 (spondyloarthritis), 9 (AS), 18 (seronegative RA), 13 (seropositive RA), 6 (pyogenic arthritis), and 22 (gout) (Table S3).

**FIGURE 3:**
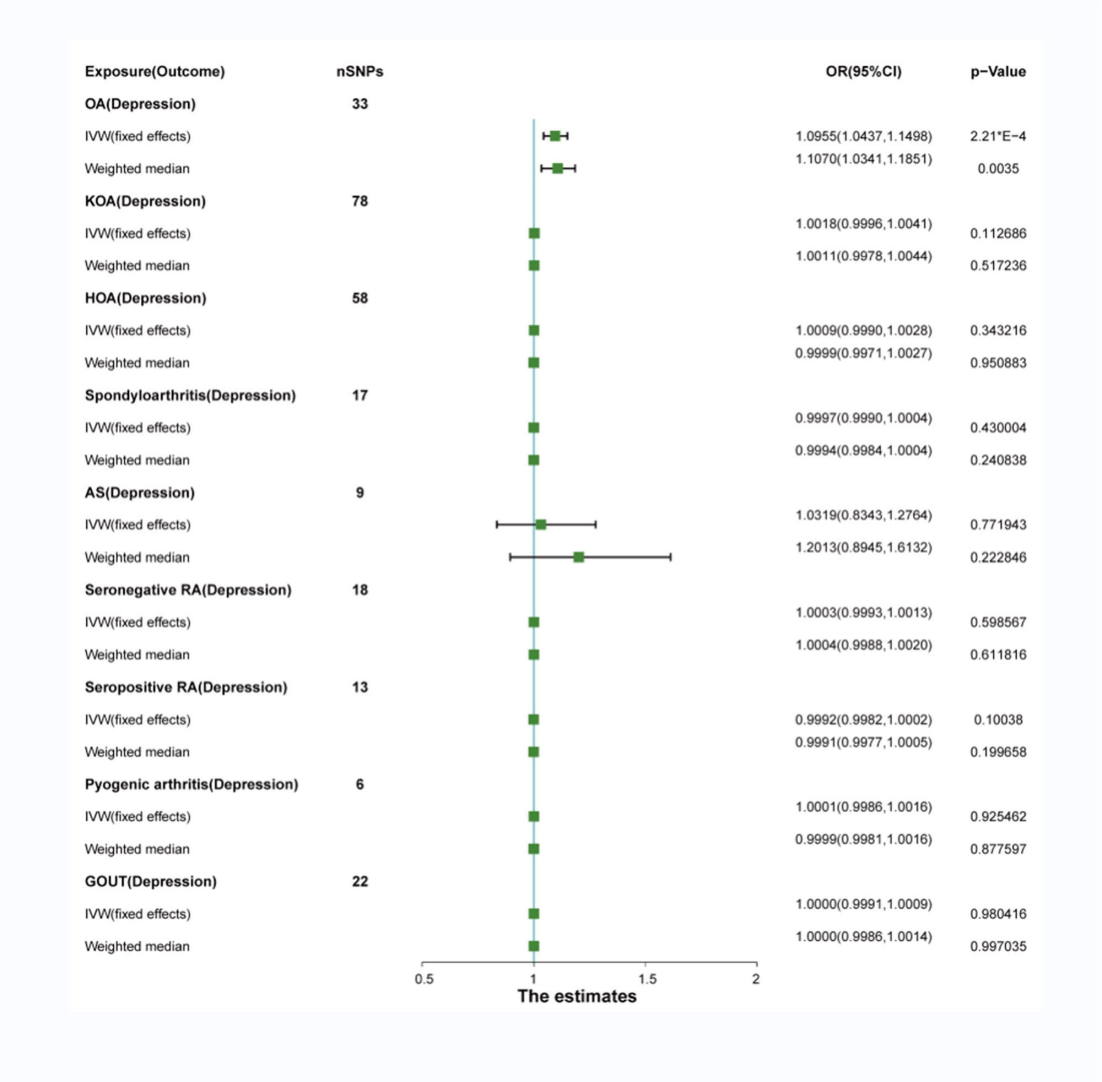
Causal relationship among different types of arthritis and depression.

Significant heterogeneity was observed only between seronegative RA and depression (seronegative RA = 0.0122) using Cochran's *Q* test, analyzed with the multiplicative‐random IVW method. No significant heterogeneity was found between other types of arthritis and depression, analyzed using the fixed‐effect IVW method (Table 2). After removing pleiotropic SNPs, a significant positive correlation was found between OA and depression (OR: 1.10; 95% CI: 1.0437–1.1498; *p* = 2.21 × 10^−4^). The weighted median model confirmed this association (OR: 1.1070; 95% CI: 1.0341–1.1851; p = 3.46 × E − 3) (Figure 3). Sensitivity analysis showed no significant horizontal pleiotropy for OA (OA = 0.5846) (Table 2). The leave‐one‐out analysis, forest plots, scatter plots, and funnel plots of MR are shown in Figure S10. However, there is no genetic correlation between other arthritis and depression (Figure 3). No significant horizontal pleiotropy was found using sensitivity analysis (KOA = 0.9118; HOA = 0.9295; sportyloarthritis = 0.0576; AS = 0.1206; seronegative RA = 0.6871; seropositive RA = 0.5197; pyogenic arthritis = 0.4453; and gout = 0.3776) (Table 2). Their MR leave‐one‐out analysis, forest plots, scatter plots, and funnel plots are shown in Figures S11–S18.

**TABLE 2:** Heterogeneity and pleiotropy analysis of different types of arthritis with depression.

			Pleiotropy test		Heterogeneity test		MR‐PRESSO	
Exposure	Outcome	nSNPs	Beta (SE)	p‐Value	Cochran's *Q*	p‐Value	RSSOBs	*p*‐Value of global test
OA	Depression	33	.0003	.5846	33.1524	.4108	36.9314	.478
KOA		78	.0003	.9118	95.3850	.0763	107.9302	.159
HOA		59	.0003	.9295	68.1531	.1480	40.8381	.067
Spondyloarthritis		17	.0002	.0576	19.8108	.2289	21.8256	.262
AS		9	.0004	.1206	15.2787	.0539	24.6937	.078
Seronegative RA		18	.0004	.6871	32.7143	.0122	59.5531	.76
Seropositive RA		13	.0005	.5197	15.6935	.2057	29.3588	.03
Pyogenic arthritis		6	.0005	.4453	1.0698	.9567	13.8990	.641
Gout		22	.0002	.3776	24.8296	.2546	27.3894	.263

Abbreviations: AS, ankylosing spondylitis; HOA, hip osteoarthritis; KOA, knee osteoarthritis; MR‐PRESSO, MR pleiotropy residual sum and outlier; OA, osteoarthritis; seronegative RA, seronegative rheumatoid arthritis; seropositive RA, seropositive rheumatoid arthritis; SNP, single‐nucleotide polymorphism.

## 4 DISCUSSION

Our results demonstrate that genetic susceptibility to depression increases the risk of developing OA and KOA. Similarly, individuals with a genetic predisposition to OA have an elevated risk of experiencing depression. However, no significant association was found between genetic predisposition to KOA and an increased risk of depression. In summary, our findings suggest a bidirectional causal relationship between depression and OA while indicating that depression only increases the risk of KOA. However, no causal relationship between depression and RA was found in the study.

Meta‐analyses have consistently found elevated levels of pro‐inflammatory cytokines in both arthritis and depression. Köhler et al.’s study, including 82 studies with 3212 MDD patients and 2798 healthy controls, revealed increased IL‐1β, IL‐6, IL‐12, IL‐13, IL‐18, IL‐1Ra, and TNF‐α levels in individuals with MDD. IL‐1β, TNF‐α, and IL‐6 are major inflammatory cytokines in OA, whereas IL‐1Ra and IL‐13 act as anti‐inflammatory factors (Akeson & Malemud, 2017; Hanns et al., 2018; Lin et al., 2021; Menard et al., 2017; Süß et al., 2020). Specifically, IL‐1β plays a key role in knee cartilage injury by promoting the breakdown of type IX collagen chain and release of COMP from cartilage (Halász et al., 2007). IL‐1β and TNF‐α play a pivotal role in the pathogenesis of OA by inducing a cascade that regulates important pathway genes. This includes NF‐kB, ERK, JNK, and p38 kinases, which perpetuate inflammation and accelerate OA progression. IL‐6 is another significant cytokine involved in OA inflammation. Binding to IL‐6 receptors activates the JAK/STAT pathway, promoting inflammation and MMP synthesis. Notably, IL‐6 receptor is present on chondrocyte membranes as well as acting as a soluble receptor in synovial fluid (Akeson & Malemud, 2017). Several studies have indicated associations between certain cytokines and OA activity. Specifically, IL‐1Ra has been linked to OA grade (Nguyen et al., 2017), whereas IL‐6, IL‐12, and IL‐13 have been correlated with knee pain and functional impairment (Giordano et al., 2020; Nees et al., 2019).

Comprehensive studies employing brain imaging, neurochemistry, and behavioral assessments have demonstrated that peripheral inflammatory factors in OA induce alterations in the central nervous system of both humans and experimental animals. Under physiological conditions, peripheral inflammatory factors remain outside the brain parenchyma. Yet, when the blood–brain barrier (BBB) is compromised, cytokines can penetrate and induce neurological symptoms. The BBB consists of specialized endothelial cells with tight junctions that prevent immune cells, blood components, and pathogens from entering the brain parenchyma. The BBB restricts over 98% of antibodies and small molecules while controlling molecular efflux. Research has demonstrated that in OA patients, the presence of IL‐6 and TNF can enhance BBB permeability, thereby facilitating the access of peripheral cytokines to the brain. This interaction may influence the central nervous system and contribute to the onset of depression. By blocking the effects of IL‐6 and TNF, the symptoms of depression can be effectively alleviated (Cheng et al., 2018; Menard et al., 2017). In a recent meta‐analysis comprising 36 randomized controlled trials, incorporating data from 10,000 patients, the supplementation of conventional depression treatment with nonsteroidal anti‐inflammatory drugs was observed to suppress the generation of diverse cytokines. This approach demonstrated enhanced efficacy in alleviating the depressive condition in comparison to the utilization of a singular antidepressant agent (Köhler‐Forsberg et al., 2019). This suggests that peripheral inflammatory factors can induce alterations in the central system.

Thus, we posit a bidirectional link between depression and OA, likely mediated by shared inflammatory factors. Why is depression not linked to other arthritis forms? Numerous studies highlight the coexistence of RA and depression. In a systematic review by Ng et al., involving 39,130 RA patients, 550,782 with depression, and 7802,230 controls, RA patients had a 47% increased depression risk, whereas those with depression had a 34% higher RA risk. RA patients with depression have an 80% higher all‐cause mortality than those without depression (Ng et al., 2022). Süß P et al. investigated the differential regulation of central nervous system myeloid and glial cells in different RA mouse models, as well as the central nervous system response to anti‐inflammatory therapy in human RA patients and mice. It is found that rheumatoid arthritis and depression can increase the incidence rate of the two through two‐way communication between the central nervous system and chronic peripheral inflammation (Süß et al., 2020). Scholars such as Nerurkar L believe that the close relationship between peripheral and brain immune responses may be a common pathophysiological mechanism between rheumatoid arthritis and depression, including the negative effects of pro‐inflammatory cytokines on monoamine neurotransmission, neurotrophic factors, and synaptic plasticity measurements (Nerurkar et al., 2019). However, MR analysis did not allow us to establish a causal relationship between depression and rheumatoid arthritis (whether seronegative or seropositive). This is not consistent with the results of previous studies. It has been reported that the main pathogenesis of RA is attributed to the abnormal proliferation of fibroblast‐like synoviocytes, which mainly express the factor CD55 in the synovium of patients with RA (Nerurkar et al., 2019), which is not the same as the key factor leading to depression. It is possible that potentially other unknown regulatory mechanisms may influence the co‐occurrence of RA and depression. This complex process, rather than a series of independent events, may involve different cell subsets and multiple regulatory pathways. Further detailed studies are needed (Smith et al., 2003).

Our study suggests a potential role of genetic predisposition to depression in elevating the risk of spondyloarthritis and gout. Nevertheless, due to limited empirical evidence, a thorough investigation is warranted to elucidate the association between the two.

Our study introduces bidirectional Mendelian randomization (MR) analysis between multiple arthritis types and depression, offering reduced susceptibility to measurement errors, reverse causation, and confounding compared to traditional observational methods. In the present study, a conservative iterative method was utilized, which ensured the consistency of data points both before and after the removal of outliers, thereby strengthening the empirical foundation. To validate the consistency of causal estimations, supplementary sensitivity analyses were conducted. Furthermore, the findings were subjected to rigorous robustness tests. As a result, the reliability of this study is firmly corroborated. Our study, despite its rigor, has limitations. (1) We could not clearly determine the overlap between exposure and outcomes. (2) The study focused solely on individuals of European ancestry, making the generalizability to other racial groups uncertain. (3) Additionally, although we controlled for confounding factors, the impact of external conditions remains a concern. (4) Perhaps a potential key mediator in the causal relationship between OA and depression. Thus, further validation of our results is necessary.

## 5 CONCLUSIONS

We have demonstrated a bidirectional causal relationship between depression and OA. At the same time, studies have found that depression only increases the risk of KOA. This provides more evidence for the diagnosis and prevention of clinical OA, KOA, and depression patients. We have not found a causal connection between rheumatoid arthritis and depression; this presents a challenge to us, rheumatoid arthritis and depression—whether there are other important and common pathogenesis that has not been found. This prompted us to do more research on rheumatoid arthritis and depression.

## AUTHOR CONTRIBUTIONS

**Min‐Yi Wu**: Writing—original draft; writing—review and editing; resources; methodology. **Yu‐Ying Liang**: Software; data curation. **Qi‐Jia Han**: Data curation; formal analysis. **Zhu Ai**: Formal analysis; funding acquisition. **Hao‐Wen Yan**: Software; funding acquisition. **Zhi‐Ming Xiang**: Conceptualization; formal analysis; project administration.

## CONFLICT OF INTEREST STATEMENT

The authors of this manuscript declare no relationships with any companies, the products or services of which may be related to the subject matter of the article.

### PEER REVIEW

The peer review history for this article is available at https://publons.com/publon/10.1002/brb3.3551.

## Supporting information

Supporting Information

Supporting Information

Supporting Information

Supporting Information

Supporting Information

Supporting Information

Supporting Information

Supporting Information

Supporting Information

Supporting Information

Supporting Information

Supporting Information

Supporting Information

Supporting Information

Supporting Information

Supporting Information

Supporting Information

Supporting Information

Supporting Information

Supporting Information

Supporting Information

## Data Availability

The datasets are available from the corresponding author on reasonable request.
