# Supplementary material for: Depression and risk of arthritis: A Mendelian randomization study
Source: Brain Behav. 2024 Jun 7;14(6):e3551. doi: 10.1002/brb3.3551 (PMC11161388; doi:10.1002/brb3.3551)
Supplement: Supplementary file 8 — Supporting Information [file BRB3-14-e3551-s011.pdf]

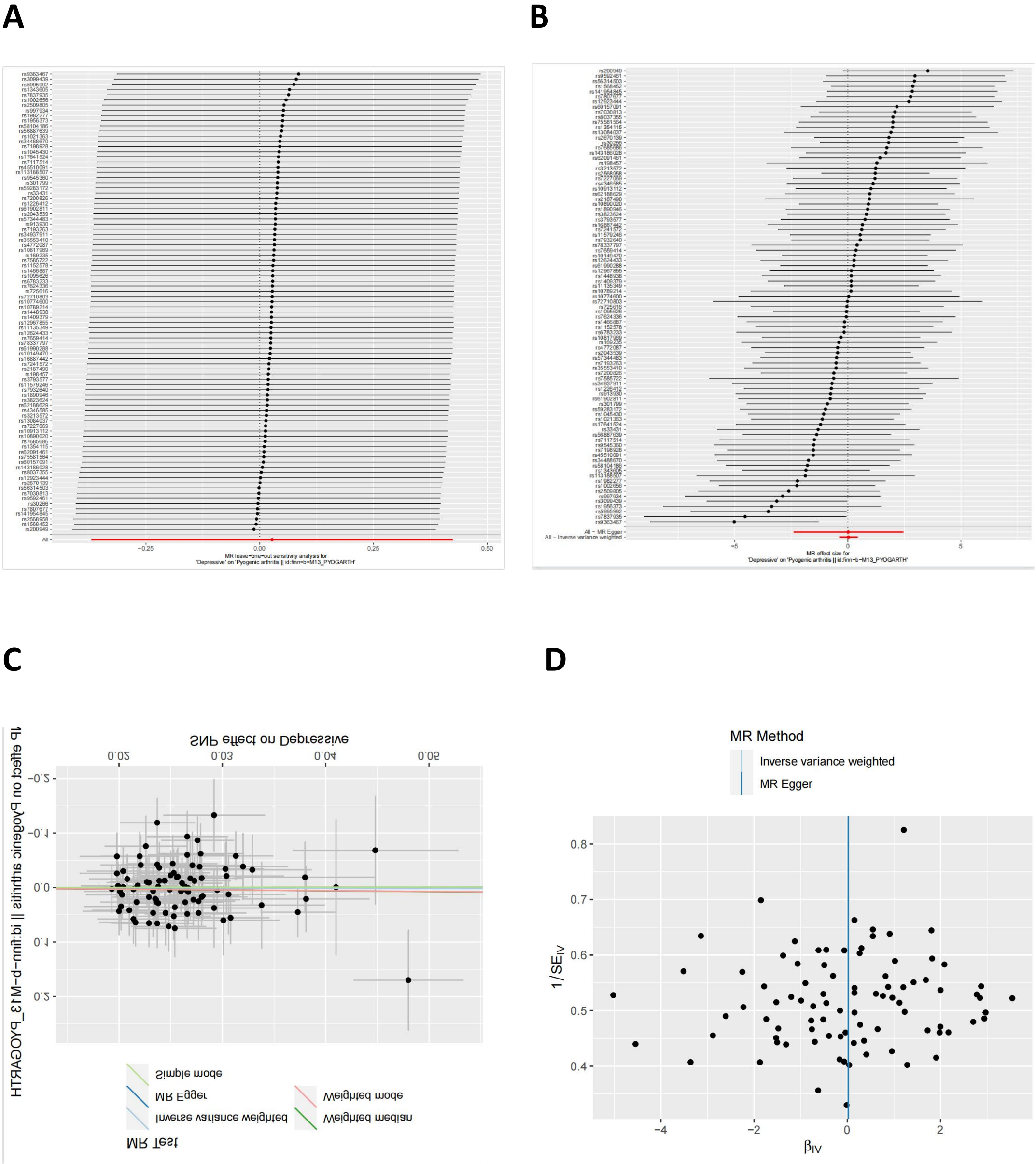


Figure S8 leave-one-out analysis(A),forest plot (B),scatter plot (C),Funnel plot (D) of the causal effect of depression on Pyogenic arthritis risk.

*MR:Mendelian randomized
