# Supplementary material for: Depression and risk of arthritis: A Mendelian randomization study
Source: Brain Behav. 2024 Jun 7;14(6):e3551. doi: 10.1002/brb3.3551 (PMC11161388; doi:10.1002/brb3.3551)
Supplement: Supplementary file 19 — Supporting Information [file BRB3-14-e3551-s004.docx]

| Table S1: Summary of data source of different Traits | | | | | | | | |
| --- | --- | --- | --- | --- | --- | --- | --- | --- |
| **Traits** | **Year** | **Consortium** | **Population** | **Sample size** | **nCases** | **nControls** | **nSNPs** | **Pubmed ID/** |
| Depression | 2019 | 23andMe, PGC and UK Biobank | European | 807553 | 246363 | 561190 | 8098588 | 30718901 |
| Depression | 2018 | MRC-IEU | European | 462,933 | 26595 | 436338 | 9851867 | https://gwas.mrcieu.ac.uk/datasets/ukb-b-12064/ |
| OS | 2017 | Neale Lab | European | 337159 | 28257 | 308902 | 10894596 | https://gwas.mrcieu.ac.uk/datasets/ukb-a-106/ |
| KOS | 2019 | NA | European | 403124 | 24955 | 378169 | 29999696 | 30664745 |
| HOS | 2019 | NA | European | 393873 | 15704 | 378169 | 29771219 | 30664745 |
| Spondyloarthritis | 2021 | NA | European | 201581 | 3037 | 198544 | 16380342 | https://gwas.mrcieu.ac.uk/datasets/finn-b-SPONDYLOARTHRITIS/ |
| AS | 2018 | MRC-IEU | European | 462933 | 1296 | 461637 | 9851867 | https://gwas.mrcieu.ac.uk/datasets/ukb-b-18194/ |
| Seronegative RA | 2021 | NA | European | 174771 | 1937 | 172834 | 16380301 | https://gwas.mrcieu.ac.uk/datasets/finn-b-RHEUMA_SERONEG/ |
| Seropositive RA | 2021 | NA | European | 215661 | 1297 | 214364 | 16380459 | https://gwas.mrcieu.ac.uk/datasets/finn-b-RHEUMA_SEROPOS_STRICT/ |
| Pyogenic arthritis | 2021 | NA | European | 148307 | 1086 | 147221 | 16380139 | https://gwas.mrcieu.ac.uk/datasets/finn-b-M13_PYOGARTH/ |
| gout | 2021 | NA | European | 150797 | 3576 | 147221 | 16380152 | https://gwas.mrcieu.ac.uk/datasets/finn-b-M13_GOUT/ |

OA：osteoarthritis;KOA：knee osteoarthritis;HOA：hip osteoarthritis;AS：ankylosing spondylitis;Seronegative RA：Seronegative rheumatoid arthritis;Seropositive RA：Seropositive rheumatoid arthritis;MR-PRESSO：MR pleiotropy residual sum and outlier;SNP：single-nucleotide polymorphism.
