# Supplementary material for: Depression and risk of arthritis: A Mendelian randomization study
Source: Brain Behav. 2024 Jun 7;14(6):e3551. doi: 10.1002/brb3.3551 (PMC11161388; doi:10.1002/brb3.3551)
Supplement: Supplementary file 20 — Supporting Information [file BRB3-14-e3551-s008.docx]

| **Table S2.** Characteristics of the SNPs related to depression and different types of arthritis | | | | | | | | | | | | | |
| --- | --- | --- | --- | --- | --- | --- | --- | --- | --- | --- | --- | --- | --- |
| SNP | Effects on depression | | | |  | Effects on OA | | | | | Chr | Position | F |
|  |  |  |  |  |  |  |  |  |  |  |  |  |  |
|  | EA | OA | Beta | SE | p-val | EA | OA | Beta | SE | p-val |  |  | statistic |
| rs1002656 | T | C | -0.0266 | 0.0038 | 3.74E-12 | T | C | -0.00087 | 0.000743 | 0.241937 | 1 | 37192741 | 49.00 |
| rs10149470 | A | G | -0.0267 | 0.0035 | 3.72E-14 | A | G | 0.000547 | 0.000675 | 0.41803 | 14 | 104017953 | 58.20 |
| rs1021363 | A | G | 0.0303 | 0.0037 | 4.41E-16 | A | G | 0.001623 | 0.000704 | 0.02116 | 10 | 106610839 | 67.06 |
| rs1045430 | T | G | -0.0253 | 0.0035 | 7.31E-13 | T | G | 0.000445 | 0.000673 | 0.508075 | 14 | 75130235 | 52.25 |
| rs10774600 | T | C | -0.0267 | 0.0048 | 3.39E-08 | T | C | 0.001442 | 0.000914 | 0.114442 | 12 | 110741356 | 30.94 |
| rs10789214 | T | C | 0.0193 | 0.0035 | 4.44E-08 | T | C | -0.0005 | 0.000681 | 0.461522 | 1 | 67146817 | 30.41 |
| rs10817969 | T | G | 0.0261 | 0.0039 | 3.11E-11 | T | G | 0.000905 | 0.000749 | 0.227052 | 9 | 119731045 | 44.79 |
| rs10890020 | A | G | -0.0277 | 0.0035 | 4.03E-15 | A | G | -0.00052 | 0.000675 | 0.438922 | 1 | 73668836 | 62.64 |
| rs10913112 | T | C | -0.0264 | 0.0036 | 3.4E-13 | T | C | -0.00013 | 0.000696 | 0.847928 | 1 | 175913828 | 53.78 |
| rs1095626 | T | C | -0.0264 | 0.0035 | 7.13E-14 | T | C | 0.000496 | 0.000683 | 0.467956 | 3 | 157977962 | 56.89 |
| rs11135349 | A | C | -0.0295 | 0.0035 | 6.04E-17 | A | C | 0.000974 | 0.000675 | 0.149114 | 5 | 164523472 | 71.04 |
| rs113188507 | A | G | 0.0221 | 0.0039 | 1.87E-08 | A | G | -0.00011 | 0.000737 | 0.882574 | 1 | 80809636 | 32.11 |
| rs1152578 | T | C | -0.0218 | 0.0035 | 6.36E-10 | T | C | -0.00013 | 0.00068 | 0.852096 | 14 | 64697037 | 38.80 |
| rs11579246 | A | G | 0.0381 | 0.0061 | 5.71E-10 | A | G | -0.00007 | 0.001188 | 0.952768 | 1 | 50559162 | 39.01 |
| rs1226412 | T | C | 0.0256 | 0.0043 | 3.46E-09 | T | C | -0.00076 | 0.000839 | 0.363508 | 2 | 157111313 | 35.44 |
| rs12624433 | A | G | 0.0233 | 0.004 | 7.44E-09 | A | G | 0.000154 | 0.000778 | 0.843306 | 20 | 44680853 | 33.93 |
| rs12923444 | A | C | -0.0214 | 0.0035 | 1.3E-09 | A | C | -0.00022 | 0.000678 | 0.746328 | 16 | 21639710 | 37.38 |
| rs12967855 | A | G | 0.0265 | 0.0037 | 1.18E-12 | A | G | 0.001202 | 0.00072 | 0.094942 | 18 | 35138245 | 51.30 |
| rs13084037 | A | G | -0.0245 | 0.0042 | 7.08E-09 | A | G | -0.00175 | 0.000806 | 0.029868 | 3 | 49214066 | 34.03 |
| rs1343605 | A | C | 0.0313 | 0.0036 | 6.23E-18 | A | C | 0.001956 | 0.000698 | 0.005064 | 13 | 53647048 | 75.59 |
| rs1354115 | A | C | 0.021 | 0.0036 | 7.08E-09 | A | C | 0.0000504 | 0.000699 | 0.942441 | 9 | 2983774 | 34.03 |
| rs1409379 | T | C | 0.0249 | 0.0041 | 1.67E-09 | T | C | 0.000484 | 0.000793 | 0.542142 | 13 | 31907741 | 36.88 |
| rs141954845 | A | G | 0.0229 | 0.0037 | 8.15E-10 | A | G | -0.00064 | 0.000686 | 0.353554 | 3 | 61196910 | 38.31 |
| rs143186028 | T | G | 0.0277 | 0.0046 | 2.29E-09 | T | G | 0.000667 | 0.00096 | 0.487061 | 20 | 39998520 | 36.26 |
| rs1448938 | A | G | 0.0214 | 0.0035 | 1.3E-09 | A | G | 0.001252 | 0.000681 | 0.066002 | 11 | 30892824 | 37.38 |
| rs1466887 | T | C | -0.0199 | 0.0036 | 4.12E-08 | T | C | 0.000934 | 0.000682 | 0.170727 | 1 | 37709328 | 30.56 |
| rs1568452 | T | C | 0.0248 | 0.0036 | 8.12E-12 | T | C | -0.00091 | 0.000694 | 0.187963 | 2 | 58012833 | 47.46 |
| rs16887442 | T | C | 0.0203 | 0.0035 | 8.62E-09 | T | C | 0.001358 | 0.000682 | 0.046328 | 7 | 82936909 | 33.64 |
| rs169235 | A | G | -0.0229 | 0.0041 | 2.98E-08 | A | G | 0.000354 | 0.000783 | 0.651433 | 1 | 181740924 | 31.20 |
| rs17641524 | T | C | -0.032 | 0.0043 | 1.52E-13 | T | C | -0.00028 | 0.00083 | 0.739685 | 1 | 197704717 | 55.38 |
| rs1890946 | T | C | -0.0235 | 0.0035 | 2.68E-11 | T | C | 0.000505 | 0.000676 | 0.455554 | 1 | 52342427 | 45.08 |
| rs1956373 | T | G | -0.0226 | 0.004 | 2.06E-08 | T | G | -0.000055 | 0.00078 | 0.943733 | 14 | 60141822 | 31.92 |
| rs1982277 | T | C | 0.0279 | 0.0041 | 1.45E-11 | T | C | -0.00033 | 0.000821 | 0.691419 | 9 | 11513617 | 46.31 |
| rs198457 | T | C | -0.0292 | 0.0046 | 2.99E-10 | T | C | 0.000707 | 0.000868 | 0.415794 | 11 | 61471678 | 40.29 |
| rs200949 | A | G | 0.048 | 0.0053 | 2.53E-19 | A | G | -0.00224 | 0.000959 | 0.019532 | 6 | 27835435 | 82.02 |
| rs2043539 | A | G | 0.0273 | 0.0035 | 9.89E-15 | A | G | 0.000483 | 0.000683 | 0.479162 | 7 | 12253880 | 60.84 |
| rs2187490 | T | G | -0.0338 | 0.0061 | 3.82E-08 | T | G | -0.00156 | 0.001154 | 0.175001 | 11 | 118713180 | 30.70 |
| rs2509805 | T | C | 0.022 | 0.0038 | 9.17E-09 | T | C | 0.000333 | 0.000723 | 0.644833 | 11 | 57650796 | 33.52 |
| rs2568958 | A | G | 0.0373 | 0.0036 | 8.47E-25 | A | G | 0.001959 | 0.000687 | 0.004329 | 1 | 72765116 | 107.35 |
| rs2670139 | T | C | -0.0266 | 0.0041 | 1.21E-10 | T | C | -0.00083 | 0.000801 | 0.30185 | 9 | 126634255 | 42.09 |
| rs301799 | T | C | -0.025 | 0.0035 | 1.36E-12 | T | C | 0.000133 | 0.000685 | 0.846012 | 1 | 8489302 | 51.02 |
| rs30266 | A | G | 0.0308 | 0.0037 | 1.45E-16 | A | G | 0.00131 | 0.000718 | 0.068039 | 5 | 103972357 | 69.29 |
| rs3099439 | T | C | -0.0276 | 0.0035 | 5.05E-15 | T | C | -0.00127 | 0.00068 | 0.060964 | 5 | 87545318 | 62.18 |
| rs3213572 | A | G | 0.0217 | 0.0035 | 7.61E-10 | A | G | 0.001061 | 0.000674 | 0.115662 | 12 | 121205078 | 38.44 |
| rs33431 | T | C | 0.0198 | 0.0036 | 4.81E-08 | T | C | 0.000112 | 0.000698 | 0.872501 | 19 | 30939989 | 30.25 |
| rs34488670 | T | C | -0.0252 | 0.0043 | 6.03E-09 | T | C | -0.00046 | 0.000827 | 0.578326 | 15 | 47684936 | 34.35 |
| rs34937911 | T | C | 0.0304 | 0.0055 | 4.13E-08 | T | C | 0.0000505 | 0.001061 | 0.962019 | 4 | 42110353 | 30.55 |
| rs35553410 | T | C | -0.0244 | 0.004 | 1.42E-09 | T | C | -0.00115 | 0.000773 | 0.136238 | 4 | 131237381 | 37.21 |
| rs3793577 | A | G | -0.0229 | 0.0035 | 8.41E-11 | A | G | -0.0014 | 0.000682 | 0.040248 | 9 | 23737627 | 42.81 |
| rs3823624 | T | C | 0.0272 | 0.0045 | 1.99E-09 | T | C | 0.001727 | 0.00088 | 0.049816 | 7 | 2110346 | 36.54 |
| rs4346585 | T | C | -0.0236 | 0.0038 | 7.13E-10 | T | C | -0.00025 | 0.000722 | 0.727472 | 3 | 44906746 | 38.57 |
| rs45510091 | A | G | 0.0448 | 0.008 | 1.83E-08 | A | G | 0.002852 | 0.001465 | 0.051509 | 4 | 123186393 | 31.36 |
| rs4772087 | T | C | 0.0227 | 0.0036 | 3.91E-10 | T | C | -0.00014 | 0.000696 | 0.842645 | 13 | 99115041 | 39.76 |
| rs56314503 | T | G | -0.0254 | 0.004 | 2.95E-10 | T | G | -0.00014 | 0.000781 | 0.860608 | 12 | 84465022 | 40.32 |
| rs56887639 | A | G | -0.0278 | 0.0039 | 1.51E-12 | A | G | -0.00031 | 0.000765 | 0.685992 | 16 | 13755530 | 50.81 |
| rs57344483 | A | G | -0.038 | 0.0068 | 1.82E-08 | A | G | -0.00261 | 0.001282 | 0.042061 | 11 | 127022560 | 31.23 |
| rs58104186 | A | G | 0.0237 | 0.0035 | 1.82E-11 | A | G | 0.000788 | 0.00068 | 0.247062 | 7 | 109099919 | 45.85 |
| rs59283172 | A | G | -0.0329 | 0.0057 | 1.02E-08 | A | G | 0.001593 | 0.001073 | 0.137831 | 9 | 25234672 | 33.32 |
| rs5995992 | T | C | -0.0266 | 0.0039 | 1.3E-11 | T | C | -0.00134 | 0.000746 | 0.072763 | 22 | 41487218 | 46.52 |
| rs60157091 | T | C | 0.02 | 0.0035 | 1.42E-08 | T | C | -0.00102 | 0.000674 | 0.13115 | 5 | 61509655 | 32.65 |
| rs61902811 | A | G | -0.0257 | 0.0036 | 1.4E-12 | A | G | -0.00117 | 0.000698 | 0.093197 | 11 | 113370758 | 50.96 |
| rs61990288 | A | G | -0.026 | 0.0035 | 1.68E-13 | A | G | -0.00068 | 0.000674 | 0.31429 | 14 | 42074726 | 55.18 |
| rs62091461 | T | C | -0.0254 | 0.0042 | 1.95E-09 | T | C | 0.001389 | 0.000797 | 0.081439 | 18 | 52488672 | 36.57 |
| rs62188629 | A | G | 0.0236 | 0.0038 | 7.13E-10 | A | G | 0.00067 | 0.000728 | 0.357181 | 2 | 208044470 | 38.57 |
| rs6783233 | T | C | 0.0218 | 0.0039 | 2.9E-8 | T | C | 0.002551 | 0.000748 | 0.000649 | 3 | 117509984 | 31.25 |
| rs7030813 | T | C | 0.0253 | 0.0036 | 3.07E-12 | T | C | 0.002246 | 0.000699 | 0.001321 | 9 | 36999369 | 49.39 |
| rs7117514 | A | G | -0.0204 | 0.0035 | 7.29E-09 | A | G | 0.000104 | 0.000676 | 0.877862 | 11 | 70544937 | 33.97 |
| rs7193263 | A | G | -0.0239 | 0.0038 | 4.33E-10 | A | G | -0.00019 | 0.000728 | 0.791593 | 16 | 6315880 | 39.56 |
| rs7198928 | T | C | 0.0239 | 0.0036 | 4.45E-11 | T | C | 0.000339 | 0.000698 | 0.627276 | 16 | 7666402 | 44.07 |
| rs7200826 | T | C | 0.028 | 0.004 | 3.74E-12 | T | C | -0.00142 | 0.000772 | 0.066761 | 16 | 13066833 | 49.00 |
| rs7227069 | A | G | 0.0238 | 0.0035 | 1.5E-11 | A | G | 0.00126 | 0.000681 | 0.064297 | 18 | 50731802 | 46.24 |
| rs7241572 | A | G | 0.028 | 0.0044 | 2.7E-10 | A | G | 0.000413 | 0.000841 | 0.623165 | 18 | 77580712 | 40.50 |
| rs725616 | T | C | 0.0204 | 0.0036 | 1.87E-08 | T | C | 0.000683 | 0.000701 | 0.329816 | 6 | 147950422 | 32.11 |
| rs72710803 | A | C | -0.041 | 0.0062 | 5.29E-11 | A | C | -0.000065 | 0.001187 | 0.956622 | 1 | 177428018 | 43.73 |
| rs75581564 | A | G | 0.0301 | 0.0054 | 3.17E-08 | A | G | 0.001324 | 0.001032 | 0.199723 | 17 | 27363750 | 31.07 |
| rs7585722 | T | C | -0.0269 | 0.0048 | 2.68E-08 | T | C | -0.00019 | 0.000935 | 0.836507 | 2 | 86819128 | 31.41 |
| rs7624336 | T | G | 0.0238 | 0.0043 | 3.96E-08 | T | G | -0.0005 | 0.000821 | 0.544112 | 3 | 53244151 | 30.63 |
| rs7659414 | A | C | -0.0201 | 0.0035 | 1.2E-8 | A | C | 0.0000573 | 0.000682 | 0.933008 | 4 | 177350956 | 32.98 |
| rs7685686 | A | G | 0.0202 | 0.0036 | 2.57E-08 | A | G | 0.000973 | 0.000683 | 0.153895 | 4 | 3207142 | 31.48 |
| rs7807677 | T | C | 0.0237 | 0.0035 | 1.82E-11 | T | C | 0.000138 | 0.000678 | 0.839059 | 7 | 117502574 | 45.85 |
| rs78337797 | T | G | 0.0306 | 0.0055 | 3.37E-08 | T | G | 0.001473 | 0.001031 | 0.152936 | 12 | 23987925 | 30.95 |
| rs7837935 | T | G | -0.0292 | 0.0049 | 3.34E-09 | T | G | 0.000449 | 0.000922 | 0.626598 | 8 | 65562019 | 35.51 |
| rs7932640 | T | C | 0.0281 | 0.0035 | 1.62E-15 | T | C | 0.000585 | 0.000683 | 0.391623 | 11 | 88744425 | 64.46 |
| rs8037355 | T | C | -0.0233 | 0.0035 | 3.94E-11 | T | C | -0.00136 | 0.000677 | 0.043959 | 15 | 37643831 | 44.32 |
| rs913930 | A | G | -0.0208 | 0.0037 | 2.42E-08 | A | G | -0.00079 | 0.000709 | 0.267595 | 9 | 120484009 | 31.60 |
| rs9363467 | T | C | 0.0237 | 0.0036 | 6.44E-11 | T | C | 0.000486 | 0.000691 | 0.48195 | 6 | 66605257 | 43.34 |
| rs9545360 | A | C | -0.0271 | 0.0046 | 5.02E-09 | A | C | -0.00023 | 0.000872 | 0.790966 | 13 | 80826373 | 34.71 |
| rs9592461 | A | G | 0.0216 | 0.0035 | 9.1E-10 | A | G | 0.001069 | 0.000674 | 0.112799 | 13 | 66941792 | 38.09 |
| rs997934 | T | C | 0.0198 | 0.0036 | 4.81E-08 | T | C | 0.000323 | 0.000695 | 0.642365 | 10 | 1795194 | 30.25 |
|  |  |  |  |  |  |  |  |  |  |  |  |  |  |
| SNP | Effects on depression | | | | | Effects on KOA | | | | | Chr | Position | F |
|  | EA | OA | Beta | SE | p-val | EA | OA | Beta | SE | p-val |  |  | statistic |
| rs1002656 | T | C | -0.0266 | 0.0038 | 3.74E-12 | T | C | -0.0036 | 0.0102 | 0.725301 | 1 | 37192741 | 49.00 |
| rs10149470 | A | G | -0.0267 | 0.0035 | 3.72E-14 | A | G | 0.0101 | 0.0093 | 0.2763 | 14 | 104017953 | 58.20 |
| rs1021363 | A | G | 0.0303 | 0.0037 | 4.41E-16 | A | G | -0.0029 | 0.0098 | 0.7689 | 10 | 106638740 | 67.06 |
| rs1045430 | T | G | -0.0253 | 0.0035 | 7.31E-13 | T | G | -0.0037 | 0.0093 | 0.687999 | 14 | 75130235 | 52.25 |
| rs10774600 | T | C | -0.0267 | 0.0048 | 3.39E-08 | T | C | 0.0085 | 0.0126 | 0.4971 | 12 | 110741356 | 30.94 |
| rs10789214 | T | C | 0.0193 | 0.0035 | 4.44E-08 | T | C | 0.003 | 0.0094 | 0.7452 | 1 | 67146817 | 30.41 |
| rs10817969 | T | G | 0.0261 | 0.0039 | 3.11E-11 | T | G | 0.0087 | 0.0103 | 0.4013 | 9 | 119731045 | 44.79 |
| rs10890020 | A | G | -0.0277 | 0.0035 | 4.03E-15 | A | G | 0.0053 | 0.0093 | 0.5689 | 1 | 73668836 | 62.64 |
| rs10913112 | T | C | -0.0264 | 0.0036 | 3.4E-13 | T | C | -0.0231 | 0.0096 | 0.01592 | 1 | 175913828 | 53.78 |
| rs1095626 | T | C | -0.0264 | 0.0035 | 7.13E-14 | T | C | 0.008 | 0.0094 | 0.3959 | 3 | 157977962 | 56.89 |
| rs11135349 | A | C | -0.0295 | 0.0035 | 6.04E-17 | A | C | -0.0103 | 0.0093 | 0.266 | 5 | 164523472 | 71.04 |
| rs113188507 | A | G | 0.0221 | 0.0039 | 1.87E-08 | A | G | 0.018 | 0.0102 | 0.076269 | 1 | 80809636 | 32.11 |
| rs1152578 | T | C | -0.0218 | 0.0035 | 6.36E-10 | T | C | -0.0053 | 0.0094 | 0.5734 | 14 | 64697037 | 38.80 |
| rs11579246 | A | G | 0.0381 | 0.0061 | 5.71E-10 | A | G | -0.0113 | 0.0164 | 0.491801 | 1 | 50559162 | 39.01 |
| rs1226412 | T | C | 0.0256 | 0.0043 | 3.46E-09 | T | C | 0.0246 | 0.0115 | 0.03302 | 2 | 157111313 | 35.44 |
| rs12624433 | A | G | 0.0233 | 0.004 | 7.44E-09 | A | G | 0.008 | 0.0107 | 0.4525 | 20 | 44680853 | 33.93 |
| rs12923444 | A | C | -0.0214 | 0.0035 | 1.3E-09 | A | C | 0.002 | 0.0093 | 0.8288 | 16 | 21639710 | 37.38 |
| rs12967855 | A | G | 0.0265 | 0.0037 | 1.18E-12 | A | G | 0.0114 | 0.0099 | 0.2505 | 18 | 35138245 | 51.30 |
| rs13084037 | A | G | -0.0245 | 0.0042 | 7.08E-09 | A | G | -0.0227 | 0.0111 | 0.04081 | 3 | 49214066 | 34.03 |
| rs1343605 | A | C | 0.0313 | 0.0036 | 6.23E-18 | A | C | 0.0094 | 0.0096 | 0.3297 | 13 | 53647048 | 75.59 |
| rs1354115 | A | C | 0.021 | 0.0036 | 7.08E-09 | A | C | 0.0129 | 0.0096 | 0.1809 | 9 | 2983774 | 34.03 |
| rs1409379 | T | C | 0.0249 | 0.0041 | 1.67E-09 | T | C | 0.0185 | 0.0109 | 0.090661 | 13 | 31907741 | 36.88 |
| rs141954845 | A | G | 0.0229 | 0.0037 | 8.15E-10 | A | G | -0.0186 | 0.0099 | 0.06108 | 3 | 61192911 | 38.31 |
| rs143186028 | T | G | 0.0277 | 0.0046 | 2.29E-09 | T | G | -0.0079 | 0.0127 | 0.5347 | 20 | 39997404 | 36.26 |
| rs1448938 | A | G | 0.0214 | 0.0035 | 1.3E-09 | A | G | 0.0233 | 0.0094 | 0.0129 | 11 | 30892824 | 37.38 |
| rs1466887 | T | C | -0.0199 | 0.0036 | 4.12E-08 | T | C | -0.007 | 0.0094 | 0.457 | 1 | 37709328 | 30.56 |
| rs1568452 | T | C | 0.0248 | 0.0036 | 8.12E-12 | T | C | -0.0004 | 0.0096 | 0.9628 | 2 | 58012833 | 47.46 |
| rs16887442 | T | C | 0.0203 | 0.0035 | 8.62E-09 | T | C | -0.0037 | 0.0094 | 0.6936 | 7 | 82936909 | 33.64 |
| rs169235 | A | G | -0.0229 | 0.0041 | 2.98E-08 | A | G | -0.0059 | 0.0108 | 0.585901 | 1 | 181740924 | 31.20 |
| rs17641524 | T | C | -0.032 | 0.0043 | 1.52E-13 | T | C | 0.0003 | 0.0114 | 0.9758 | 1 | 197704717 | 55.38 |
| rs1890946 | T | C | -0.0235 | 0.0035 | 2.68E-11 | T | C | 0.0025 | 0.0093 | 0.788099 | 1 | 52342427 | 45.08 |
| rs1956373 | T | G | -0.0226 | 0.004 | 2.06E-08 | T | G | -0.0024 | 0.0107 | 0.8263 | 14 | 60141822 | 31.92 |
| rs1982277 | T | C | 0.0279 | 0.0041 | 1.45E-11 | T | C | 0.0057 | 0.0115 | 0.6158 | 9 | 11513019 | 46.31 |
| rs2043539 | A | G | 0.0273 | 0.0035 | 9.89E-15 | A | G | 0.0184 | 0.0094 | 0.04991 | 7 | 12253880 | 60.84 |
| rs2187490 | T | G | -0.0338 | 0.0061 | 3.82E-08 | T | G | 0.0181 | 0.0159 | 0.2561 | 11 | 118713180 | 30.70 |
| rs2509805 | T | C | 0.022 | 0.0038 | 9.17E-09 | T | C | -0.0084 | 0.01 | 0.4002 | 11 | 57650796 | 33.52 |
| rs2568958 | A | G | 0.0373 | 0.0036 | 8.47E-25 | A | G | 0.014 | 0.0095 | 0.1404 | 1 | 72765116 | 107.35 |
| rs2670139 | T | C | -0.0266 | 0.0041 | 1.21E-10 | T | C | 0.0022 | 0.011 | 0.8423 | 9 | 126634255 | 42.09 |
| rs301799 | T | C | -0.025 | 0.0035 | 1.36E-12 | T | C | -0.0029 | 0.0094 | 0.7618 | 1 | 8489302 | 51.02 |
| rs30266 | A | G | 0.0308 | 0.0037 | 1.45E-16 | A | G | 0.0106 | 0.0099 | 0.2841 | 5 | 103972357 | 69.29 |
| rs3099439 | T | C | -0.0276 | 0.0035 | 5.05E-15 | T | C | 0.0081 | 0.0094 | 0.3846 | 5 | 87545318 | 62.18 |
| rs3213572 | A | G | 0.0217 | 0.0035 | 7.61E-10 | A | G | -0.0086 | 0.0093 | 0.3566 | 12 | 121205078 | 38.44 |
| rs33431 | T | C | 0.0198 | 0.0036 | 4.81E-08 | T | C | 0.0254 | 0.0096 | 0.008131 | 19 | 30939989 | 30.25 |
| rs34488670 | T | C | -0.0252 | 0.0043 | 6.03E-09 | T | C | 0.0083 | 0.0114 | 0.4667 | 15 | 47684936 | 34.35 |
| rs34937911 | T | C | 0.0304 | 0.0055 | 4.13E-08 | T | C | 0.0091 | 0.0146 | 0.5328 | 4 | 42110353 | 30.55 |
| rs35553410 | T | C | -0.0244 | 0.004 | 1.42E-09 | T | C | -0.0364 | 0.0106 | 0.000633 | 4 | 131237381 | 37.21 |
| rs3823624 | T | C | 0.0272 | 0.0045 | 1.99E-09 | T | C | 0.0299 | 0.0121 | 0.01355 | 7 | 2110346 | 36.54 |
| rs4346585 | T | C | -0.0236 | 0.0038 | 7.13E-10 | T | C | -0.0088 | 0.0105 | 0.4045 | 3 | 44736493 | 38.57 |
| rs45510091 | A | G | 0.0448 | 0.008 | 1.83E-08 | A | G | 0.0467 | 0.0203 | 0.02139 | 4 | 123186393 | 31.36 |
| rs4772087 | T | C | 0.0227 | 0.0036 | 3.91E-10 | T | C | 0.0065 | 0.0096 | 0.5002 | 13 | 99115041 | 39.76 |
| rs56314503 | T | G | -0.0254 | 0.004 | 2.95E-10 | T | G | -0.0238 | 0.0107 | 0.0266 | 12 | 84465022 | 40.32 |
| rs56887639 | A | G | -0.0278 | 0.0039 | 1.51E-12 | A | G | 0.0087 | 0.0105 | 0.4099 | 16 | 13755530 | 50.81 |
| rs57344483 | A | G | -0.038 | 0.0068 | 1.82E-08 | A | G | 0.0094 | 0.0176 | 0.5936 | 11 | 127022560 | 31.23 |
| rs58104186 | A | G | 0.0237 | 0.0035 | 1.82E-11 | A | G | 0.0074 | 0.0094 | 0.43 | 7 | 109099919 | 45.85 |
| rs59283172 | A | G | -0.0329 | 0.0057 | 1.02E-08 | A | G | 0.0181 | 0.0158 | 0.2521 | 9 | 25232978 | 33.32 |
| rs5995992 | T | C | -0.0266 | 0.0039 | 1.3E-11 | T | C | 0.0096 | 0.0103 | 0.3478 | 22 | 41487218 | 46.52 |
| rs60157091 | T | C | 0.02 | 0.0035 | 1.42E-08 | T | C | -0.008 | 0.0093 | 0.3881 | 5 | 61509655 | 32.65 |
| rs61902811 | A | G | -0.0257 | 0.0036 | 1.4E-12 | A | G | -0.0231 | 0.0096 | 0.01636 | 11 | 113370758 | 50.96 |
| rs61990288 | A | G | -0.026 | 0.0035 | 1.68E-13 | A | G | -0.0179 | 0.0093 | 0.05341 | 14 | 42074726 | 55.18 |
| rs62091461 | T | C | -0.0254 | 0.0042 | 1.95E-09 | T | C | 0.0215 | 0.011 | 0.04987 | 18 | 52488672 | 36.57 |
| rs62188629 | A | G | 0.0236 | 0.0038 | 7.13E-10 | A | G | 0.0099 | 0.01 | 0.3217 | 2 | 208044470 | 38.57 |
| rs6783233 | T | C | 0.0218 | 0.0039 | 2.9E-8 | T | C | 0.0263 | 0.0103 | 0.01054 | 3 | 117509984 | 31.25 |
| rs7030813 | T | C | 0.0253 | 0.0036 | 3.07E-12 | T | C | 0.0057 | 0.0096 | 0.5554 | 9 | 36999369 | 49.39 |
| rs7117514 | A | G | -0.0204 | 0.0035 | 7.29E-09 | A | G | -0.0018 | 0.0093 | 0.8502 | 11 | 70544937 | 33.97 |
| rs7193263 | A | G | -0.0239 | 0.0038 | 4.33E-10 | A | G | -0.0024 | 0.01 | 0.8105 | 16 | 6315880 | 39.56 |
| rs7198928 | T | C | 0.0239 | 0.0036 | 4.45E-11 | T | C | 0.0045 | 0.0096 | 0.6427 | 16 | 7666402 | 44.07 |
| rs7200826 | T | C | 0.028 | 0.004 | 3.74E-12 | T | C | -0.0143 | 0.0106 | 0.1776 | 16 | 13066833 | 49.00 |
| rs7227069 | A | G | 0.0238 | 0.0035 | 1.5E-11 | A | G | 0.0203 | 0.0094 | 0.03006 | 18 | 50731802 | 46.24 |
| rs7241572 | A | G | 0.028 | 0.0044 | 2.7E-10 | A | G | 0.0035 | 0.0116 | 0.7595 | 18 | 77580712 | 40.50 |
| rs725616 | T | C | 0.0204 | 0.0036 | 1.87E-08 | T | C | 0.0347 | 0.0097 | 0.000319 | 6 | 147950422 | 32.11 |
| rs72710803 | A | C | -0.041 | 0.0062 | 5.29E-11 | A | C | -0.0081 | 0.0163 | 0.6201 | 1 | 177428018 | 43.73 |
| rs75581564 | A | G | 0.0301 | 0.0054 | 3.17E-08 | A | G | 0.0277 | 0.0142 | 0.05106 | 17 | 27363750 | 31.07 |
| rs7585722 | T | C | -0.0269 | 0.0048 | 2.68E-08 | T | C | -0.0043 | 0.0129 | 0.736999 | 2 | 86819128 | 31.41 |
| rs7624336 | T | G | 0.0238 | 0.0043 | 3.96E-08 | T | G | 0.0068 | 0.0113 | 0.549099 | 3 | 53244151 | 30.63 |
| rs7659414 | A | C | -0.0201 | 0.0035 | 1.2E-8 | A | C | -0.0106 | 0.0094 | 0.2577 | 4 | 177350956 | 32.98 |
| rs7685686 | A | G | 0.0202 | 0.0036 | 2.57E-08 | A | G | 0.0141 | 0.0094 | 0.1345 | 4 | 3207142 | 31.48 |
| rs7807677 | T | C | 0.0237 | 0.0035 | 1.82E-11 | T | C | -0.0173 | 0.0093 | 0.064061 | 7 | 117502574 | 45.85 |
| rs78337797 | T | G | 0.0306 | 0.0055 | 3.37E-08 | T | G | 0.0529 | 0.0142 | 0.000192 | 12 | 23987925 | 30.95 |
| rs7837935 | T | G | -0.0292 | 0.0049 | 3.34E-09 | T | G | -0.0045 | 0.0127 | 0.7224 | 8 | 65562019 | 35.51 |
| rs7932640 | T | C | 0.0281 | 0.0035 | 1.62E-15 | T | C | 0.0157 | 0.0094 | 0.09551 | 11 | 88744425 | 64.46 |
| rs8037355 | T | C | -0.0233 | 0.0035 | 3.94E-11 | T | C | -0.0335 | 0.0093 | 0.000325 | 15 | 37643831 | 44.32 |
| rs913930 | A | G | -0.0208 | 0.0037 | 2.42E-08 | A | G | -0.0066 | 0.0097 | 0.499001 | 9 | 120484009 | 31.60 |
| rs9363467 | T | C | 0.0237 | 0.0036 | 6.44E-11 | T | C | 0.0236 | 0.0101 | 0.01962 | 6 | 66565703 | 43.34 |
| rs9545360 | A | C | -0.0271 | 0.0046 | 5.02E-09 | A | C | 0.0175 | 0.012 | 0.144 | 13 | 80826373 | 34.71 |
| rs9592461 | A | G | 0.0216 | 0.0035 | 9.1E-10 | A | G | 0.0174 | 0.0093 | 0.060111 | 13 | 66941792 | 38.09 |
| rs997934 | T | C | 0.0198 | 0.0036 | 4.81E-08 | T | C | -0.0092 | 0.0096 | 0.3346 | 10 | 1795194 | 30.25 |
|  |  |  |  |  |  |  |  |  |  |  |  |  |  |
| SNP | Effects on depression | | | | | Effects on HOA | | | | | Chr | Position | F |
|  | EA | OA | Beta | SE | p-val | EA | OA | Beta | SE | p-val |  |  | statistic |
| rs1002656 | T | C | -0.0266 | 0.0038 | 3.74E-12 | T | C | -0.0057 | 0.0128 | 0.656 | 1 | 37192741 | 49.00 |
| rs10149470 | A | G | -0.0267 | 0.0035 | 3.72E-14 | A | G | 0.0015 | 0.0117 | 0.9006 | 14 | 104017953 | 58.20 |
| rs1021363 | A | G | 0.0303 | 0.0037 | 4.41E-16 | A | G | -0.0065 | 0.0122 | 0.592899 | 10 | 106610839 | 67.06 |
| rs1045430 | T | G | -0.0253 | 0.0035 | 7.31E-13 | T | G | 0.0204 | 0.0116 | 0.07915 | 14 | 75130235 | 52.25 |
| rs10774600 | T | C | -0.0267 | 0.0048 | 3.39E-08 | T | C | -0.0092 | 0.0159 | 0.5598 | 12 | 110741356 | 30.94 |
| rs10789214 | T | C | 0.0193 | 0.0035 | 4.44E-08 | T | C | -0.0222 | 0.0118 | 0.05889 | 1 | 67146817 | 30.41 |
| rs10817969 | T | G | 0.0261 | 0.0039 | 3.11E-11 | T | G | -0.0045 | 0.013 | 0.7294 | 9 | 119731045 | 44.79 |
| rs10890020 | A | G | -0.0277 | 0.0035 | 4.03E-15 | A | G | -0.001 | 0.0117 | 0.9296 | 1 | 73668836 | 62.64 |
| rs10913112 | T | C | -0.0264 | 0.0036 | 3.4E-13 | T | C | -0.0285 | 0.012 | 0.01812 | 1 | 175913828 | 53.78 |
| rs1095626 | T | C | -0.0264 | 0.0035 | 7.13E-14 | T | C | 0.0123 | 0.0118 | 0.2975 | 3 | 157977962 | 56.89 |
| rs11135349 | A | C | -0.0295 | 0.0035 | 6.04E-17 | A | C | 0.0017 | 0.0117 | 0.8814 | 5 | 164523472 | 71.04 |
| rs113188507 | A | G | 0.0221 | 0.0039 | 1.87E-08 | A | G | 0.0281 | 0.0127 | 0.02735 | 1 | 80809636 | 32.11 |
| rs1152578 | T | C | -0.0218 | 0.0035 | 6.36E-10 | T | C | -0.0046 | 0.0118 | 0.6938 | 14 | 64697037 | 38.80 |
| rs11579246 | A | G | 0.0381 | 0.0061 | 5.71E-10 | A | G | 0.0161 | 0.0206 | 0.4339 | 1 | 50559162 | 39.01 |
| rs1226412 | T | C | 0.0256 | 0.0043 | 3.46E-09 | T | C | 0.0117 | 0.0145 | 0.4168 | 2 | 157111313 | 35.44 |
| rs12624433 | A | G | 0.0233 | 0.004 | 7.44E-09 | A | G | 0.0233 | 0.0134 | 0.082389 | 20 | 44680853 | 33.93 |
| rs12923444 | A | C | -0.0214 | 0.0035 | 1.3E-09 | A | C | 0.0137 | 0.0117 | 0.2412 | 16 | 21639710 | 37.38 |
| rs12967855 | A | G | 0.0265 | 0.0037 | 1.18E-12 | A | G | -0.012 | 0.0124 | 0.3334 | 18 | 35138245 | 51.30 |
| rs13084037 | A | G | -0.0245 | 0.0042 | 7.08E-09 | A | G | -0.0171 | 0.0139 | 0.2197 | 3 | 49214066 | 34.03 |
| rs1343605 | A | C | 0.0313 | 0.0036 | 6.23E-18 | A | C | 0.0067 | 0.0121 | 0.5772 | 13 | 53647048 | 75.59 |
| rs1354115 | A | C | 0.021 | 0.0036 | 7.08E-09 | A | C | 0.0072 | 0.0121 | 0.550301 | 9 | 2983774 | 34.03 |
| rs1409379 | T | C | 0.0249 | 0.0041 | 1.67E-09 | T | C | -0.0071 | 0.0137 | 0.6031 | 13 | 31907741 | 36.88 |
| rs141954845 | A | G | 0.0229 | 0.0037 | 8.15E-10 | A | G | -0.034 | 0.0128 | 0.008098 | 3 | 61192911 | 38.31 |
| rs143186028 | T | G | 0.0277 | 0.0046 | 2.29E-09 | T | G | 0.0047 | 0.0164 | 0.777301 | 20 | 39997404 | 36.26 |
| rs1448938 | A | G | 0.0214 | 0.0035 | 1.3E-09 | A | G | 0.0206 | 0.0118 | 0.08067 | 11 | 30892824 | 37.38 |
| rs1466887 | T | C | -0.0199 | 0.0036 | 4.12E-08 | T | C | -0.0084 | 0.0118 | 0.476 | 1 | 37709328 | 30.56 |
| rs1568452 | T | C | 0.0248 | 0.0036 | 8.12E-12 | T | C | 0.0262 | 0.012 | 0.0293 | 2 | 58012833 | 47.46 |
| rs16887442 | T | C | 0.0203 | 0.0035 | 8.62E-09 | T | C | 0.0024 | 0.0118 | 0.8407 | 7 | 82936909 | 33.64 |
| rs169235 | A | G | -0.0229 | 0.0041 | 2.98E-08 | A | G | -0.0255 | 0.0135 | 0.058979 | 1 | 181740924 | 31.20 |
| rs17641524 | T | C | -0.032 | 0.0043 | 1.52E-13 | T | C | 0.0004 | 0.0143 | 0.9789 | 1 | 197704717 | 55.38 |
| rs1890946 | T | C | -0.0235 | 0.0035 | 2.68E-11 | T | C | 0.03 | 0.0117 | 0.0104 | 1 | 52342427 | 45.08 |
| rs1956373 | T | G | -0.0226 | 0.004 | 2.06E-08 | T | G | 0.0083 | 0.0135 | 0.5399 | 14 | 60141822 | 31.92 |
| rs1982277 | T | C | 0.0279 | 0.0041 | 1.45E-11 | T | C | 0.0169 | 0.0148 | 0.2517 | 9 | 11513019 | 46.31 |
| rs198457 | T | C | -0.0292 | 0.0046 | 2.99E-10 | T | C | -0.0189 | 0.015 | 0.2081 | 11 | 61471678 | 40.29 |
| rs200949 | A | G | 0.048 | 0.0053 | 2.53E-19 | A | G | 0.0011 | 0.0167 | 0.9492 | 6 | 27835435 | 82.02 |
| rs2043539 | A | G | 0.0273 | 0.0035 | 9.89E-15 | A | G | 0.016 | 0.0118 | 0.1757 | 7 | 12253880 | 60.84 |
| rs2187490 | T | G | -0.0338 | 0.0061 | 3.82E-08 | T | G | 0.0318 | 0.02 | 0.1119 | 11 | 118713180 | 30.70 |
| rs2509805 | T | C | 0.022 | 0.0038 | 9.17E-09 | T | C | 0.0304 | 0.0125 | 0.01506 | 11 | 57650796 | 33.52 |
| rs2568958 | A | G | 0.0373 | 0.0036 | 8.47E-25 | A | G | 0.021 | 0.0119 | 0.07785 | 1 | 72765116 | 107.35 |
| rs2670139 | T | C | -0.0266 | 0.0041 | 1.21E-10 | T | C | -0.0205 | 0.0138 | 0.1383 | 9 | 126634255 | 42.09 |
| rs301799 | T | C | -0.025 | 0.0035 | 1.36E-12 | T | C | 0.0162 | 0.0118 | 0.1719 | 1 | 8489302 | 51.02 |
| rs30266 | A | G | 0.0308 | 0.0037 | 1.45E-16 | A | G | 0.005 | 0.0124 | 0.6891 | 5 | 103972357 | 69.29 |
| rs3099439 | T | C | -0.0276 | 0.0035 | 5.05E-15 | T | C | -0.004 | 0.0117 | 0.735201 | 5 | 87545318 | 62.18 |
| rs3213572 | A | G | 0.0217 | 0.0035 | 7.61E-10 | A | G | 0.0022 | 0.0117 | 0.8522 | 12 | 121205078 | 38.44 |
| rs33431 | T | C | 0.0198 | 0.0036 | 4.81E-08 | T | C | -0.0069 | 0.012 | 0.5651 | 19 | 30939989 | 30.25 |
| rs34488670 | T | C | -0.0252 | 0.0043 | 6.03E-09 | T | C | 0.0008 | 0.0143 | 0.9559 | 15 | 47684936 | 34.35 |
| rs34937911 | T | C | 0.0304 | 0.0055 | 4.13E-08 | T | C | 0.0089 | 0.0183 | 0.6262 | 4 | 42110353 | 30.55 |
| rs35553410 | T | C | -0.0244 | 0.004 | 1.42E-09 | T | C | -0.0213 | 0.0134 | 0.1101 | 4 | 131237381 | 37.21 |
| rs3793577 | A | G | -0.0229 | 0.0035 | 8.41E-11 | A | G | -0.0045 | 0.0118 | 0.7059 | 9 | 23737627 | 42.81 |
| rs3823624 | T | C | 0.0272 | 0.0045 | 1.99E-09 | T | C | 0.0215 | 0.0152 | 0.1567 | 7 | 2110346 | 36.54 |
| rs4346585 | T | C | -0.0236 | 0.0038 | 7.13E-10 | T | C | 0.002 | 0.0136 | 0.8851 | 3 | 44736493 | 38.57 |
| rs45510091 | A | G | 0.0448 | 0.008 | 1.83E-08 | A | G | 0.0048 | 0.0275 | 0.8605 | 4 | 123418006 | 31.36 |
| rs4772087 | T | C | 0.0227 | 0.0036 | 3.91E-10 | T | C | 0.024 | 0.012 | 0.04603 | 13 | 99115041 | 39.76 |
| rs56314503 | T | G | -0.0254 | 0.004 | 2.95E-10 | T | G | -0.0028 | 0.0135 | 0.8337 | 12 | 84465022 | 40.32 |
| rs56887639 | A | G | -0.0278 | 0.0039 | 1.51E-12 | A | G | -0.002 | 0.0133 | 0.8803 | 16 | 13755530 | 50.81 |
| rs57344483 | A | G | -0.038 | 0.0068 | 1.82E-08 | A | G | 0.0048 | 0.0221 | 0.8282 | 11 | 127022560 | 31.23 |
| rs58104186 | A | G | 0.0237 | 0.0035 | 1.82E-11 | A | G | -0.0059 | 0.0117 | 0.616401 | 7 | 109099919 | 45.85 |
| rs59283172 | A | G | -0.0329 | 0.0057 | 1.02E-08 | A | G | -0.0193 | 0.0204 | 0.3438 | 9 | 25232978 | 33.32 |
| rs5995992 | T | C | -0.0266 | 0.0039 | 1.3E-11 | T | C | -0.0161 | 0.0129 | 0.2114 | 22 | 41487218 | 46.52 |
| rs60157091 | T | C | 0.02 | 0.0035 | 1.42E-08 | T | C | 0.0043 | 0.0116 | 0.7104 | 5 | 61509655 | 32.65 |
| rs61902811 | A | G | -0.0257 | 0.0036 | 1.4E-12 | A | G | -0.0098 | 0.0121 | 0.4149 | 11 | 113370758 | 50.96 |
| rs61990288 | A | G | -0.026 | 0.0035 | 1.68E-13 | A | G | -0.0064 | 0.0117 | 0.5835 | 14 | 42074726 | 55.18 |
| rs62091461 | T | C | -0.0254 | 0.0042 | 1.95E-09 | T | C | 0.0032 | 0.0138 | 0.8165 | 18 | 52488672 | 36.57 |
| rs62188629 | A | G | 0.0236 | 0.0038 | 7.13E-10 | A | G | -0.0105 | 0.0126 | 0.4028 | 2 | 208044470 | 38.57 |
| rs6783233 | T | C | 0.0218 | 0.0039 | 2.9E-8 | T | C | -0.0011 | 0.0129 | 0.9299 | 3 | 117509984 | 31.25 |
| rs7030813 | T | C | 0.0253 | 0.0036 | 3.07E-12 | T | C | 0.0034 | 0.0121 | 0.777301 | 9 | 36999369 | 49.39 |
| rs7117514 | A | G | -0.0204 | 0.0035 | 7.29E-09 | A | G | 0.0008 | 0.0117 | 0.9425 | 11 | 70544937 | 33.97 |
| rs7193263 | A | G | -0.0239 | 0.0038 | 4.33E-10 | A | G | -0.0043 | 0.0126 | 0.733301 | 16 | 6315880 | 39.56 |
| rs7198928 | T | C | 0.0239 | 0.0036 | 4.45E-11 | T | C | 0.0034 | 0.0121 | 0.7778 | 16 | 7666402 | 44.07 |
| rs7200826 | T | C | 0.028 | 0.004 | 3.74E-12 | T | C | -0.0077 | 0.0134 | 0.5668 | 16 | 13066833 | 49.00 |
| rs7227069 | A | G | 0.0238 | 0.0035 | 1.5E-11 | A | G | 0.0166 | 0.0118 | 0.1582 | 18 | 50731802 | 46.24 |
| rs7241572 | A | G | 0.028 | 0.0044 | 2.7E-10 | A | G | 0.0076 | 0.0145 | 0.599799 | 18 | 77580712 | 40.50 |
| rs725616 | T | C | 0.0204 | 0.0036 | 1.87E-08 | T | C | -0.0033 | 0.0121 | 0.7887 | 6 | 147950422 | 32.11 |
| rs72710803 | A | C | -0.041 | 0.0062 | 5.29E-11 | A | C | -0.015 | 0.0205 | 0.463 | 1 | 177428018 | 43.73 |
| rs75581564 | A | G | 0.0301 | 0.0054 | 3.17E-08 | A | G | 0.0015 | 0.0178 | 0.9317 | 17 | 27363750 | 31.07 |
| rs7585722 | T | C | -0.0269 | 0.0048 | 2.68E-08 | T | C | 0.01 | 0.0162 | 0.5371 | 2 | 86819128 | 31.41 |
| rs7624336 | T | G | 0.0238 | 0.0043 | 3.96E-08 | T | G | -0.0255 | 0.0142 | 0.07349 | 3 | 53244151 | 30.63 |
| rs7659414 | A | C | -0.0201 | 0.0035 | 1.2E-8 | A | C | 0.0059 | 0.0118 | 0.6145 | 4 | 177350956 | 32.98 |
| rs7685686 | A | G | 0.0202 | 0.0036 | 2.57E-08 | A | G | -0.0005 | 0.0118 | 0.9675 | 4 | 3207142 | 31.48 |
| rs7807677 | T | C | 0.0237 | 0.0035 | 1.82E-11 | T | C | -0.0078 | 0.0117 | 0.5069 | 7 | 117502574 | 45.85 |
| rs78337797 | T | G | 0.0306 | 0.0055 | 3.37E-08 | T | G | 0.0208 | 0.0178 | 0.2445 | 12 | 23987925 | 30.95 |
| rs7837935 | T | G | -0.0292 | 0.0049 | 3.34E-09 | T | G | 0.0147 | 0.016 | 0.3572 | 8 | 65562019 | 35.51 |
| rs7932640 | T | C | 0.0281 | 0.0035 | 1.62E-15 | T | C | -0.0197 | 0.0118 | 0.094211 | 11 | 88744425 | 64.46 |
| rs8037355 | T | C | -0.0233 | 0.0035 | 3.94E-11 | T | C | 0.0072 | 0.0117 | 0.5382 | 15 | 37643831 | 44.32 |
| rs913930 | A | G | -0.0208 | 0.0037 | 2.42E-08 | A | G | 0.0114 | 0.0122 | 0.3501 | 9 | 120484009 | 31.60 |
| rs9363467 | T | C | 0.0237 | 0.0036 | 6.44E-11 | T | C | 0.0017 | 0.0131 | 0.8989 | 6 | 66565703 | 43.34 |
| rs9545360 | A | C | -0.0271 | 0.0046 | 5.02E-09 | A | C | 0.005 | 0.0151 | 0.740399 | 13 | 80826373 | 34.71 |
| rs9592461 | A | G | 0.0216 | 0.0035 | 9.1E-10 | A | G | -0.0079 | 0.0117 | 0.4983 | 13 | 66941792 | 38.09 |
| rs997934 | T | C | 0.0198 | 0.0036 | 4.81E-08 | T | C | -0.0038 | 0.012 | 0.7523 | 10 | 1795194 | 30.25 |
|  |  |  |  |  |  |  |  |  |  |  |  |  |  |
| SNP | Effects on depression | | | | | Effects on Spondyloarthritis | | | | | Chr | Position | F |
|  | EA | OA | Beta | SE | p-val | EA | OA | Beta | SE | p-val |  |  | statistic |
| rs1002656 | T | C | -0.0266 | 0.0038 | 3.74E-12 | T | C | -0.0379 | 0.0289 | 0.1896 | 1 | 37192741 | 49.00 |
| rs10149470 | A | G | -0.0267 | 0.0035 | 3.72E-14 | A | G | -0.0376 | 0.027 | 0.164 | 14 | 104017953 | 58.20 |
| rs1021363 | A | G | 0.0303 | 0.0037 | 4.41E-16 | A | G | -0.0163 | 0.0301 | 0.5883 | 10 | 106610839 | 67.06 |
| rs1045430 | T | G | -0.0253 | 0.0035 | 7.31E-13 | T | G | -0.0077 | 0.0268 | 0.7751 | 14 | 75130235 | 52.25 |
| rs10774600 | T | C | -0.0267 | 0.0048 | 3.39E-08 | T | C | -0.0431 | 0.0414 | 0.2983 | 12 | 110741356 | 30.94 |
| rs10789214 | T | C | 0.0193 | 0.0035 | 4.44E-08 | T | C | -0.0032 | 0.027 | 0.9073 | 1 | 67146817 | 30.41 |
| rs10817969 | T | G | 0.0261 | 0.0039 | 3.11E-11 | T | G | 0.0182 | 0.0287 | 0.5265 | 9 | 119731045 | 44.79 |
| rs10890020 | A | G | -0.0277 | 0.0035 | 4.03E-15 | A | G | 0.0197 | 0.0269 | 0.462701 | 1 | 73669845 | 62.64 |
| rs10913112 | T | C | -0.0264 | 0.0036 | 3.4E-13 | T | C | -0.0251 | 0.0277 | 0.3661 | 1 | 175913828 | 53.78 |
| rs1095626 | T | C | -0.0264 | 0.0035 | 7.13E-14 | T | C | -0.0181 | 0.0268 | 0.501 | 3 | 157977962 | 56.89 |
| rs11135349 | A | C | -0.0295 | 0.0035 | 6.04E-17 | A | C | 0.0014 | 0.0275 | 0.9609 | 5 | 164523472 | 71.04 |
| rs113188507 | A | G | 0.0221 | 0.0039 | 1.87E-08 | A | G | 0.0057 | 0.0338 | 0.8656 | 1 | 80809636 | 32.11 |
| rs1152578 | T | C | -0.0218 | 0.0035 | 6.36E-10 | T | C | 0.0034 | 0.0271 | 0.8992 | 14 | 64697037 | 38.80 |
| rs11579246 | A | G | 0.0381 | 0.0061 | 5.71E-10 | A | G | -0.0209 | 0.037 | 0.5724 | 1 | 50559162 | 39.01 |
| rs1226412 | T | C | 0.0256 | 0.0043 | 3.46E-09 | T | C | -0.0348 | 0.0311 | 0.264 | 2 | 157111313 | 35.44 |
| rs12624433 | A | G | 0.0233 | 0.004 | 7.44E-09 | A | G | 0.0046 | 0.0305 | 0.8795 | 20 | 44680853 | 33.93 |
| rs12923444 | A | C | -0.0214 | 0.0035 | 1.3E-09 | A | C | -0.0386 | 0.0276 | 0.1617 | 16 | 21639710 | 37.38 |
| rs12967855 | A | G | 0.0265 | 0.0037 | 1.18E-12 | A | G | 0.044 | 0.0304 | 0.1478 | 18 | 35138245 | 51.30 |
| rs13084037 | A | G | -0.0245 | 0.0042 | 7.08E-09 | A | G | -0.0157 | 0.0366 | 0.668199 | 3 | 49214066 | 34.03 |
| rs1343605 | A | C | 0.0313 | 0.0036 | 6.23E-18 | A | C | 0.0031 | 0.0277 | 0.9112 | 13 | 53647048 | 75.59 |
| rs1354115 | A | C | 0.021 | 0.0036 | 7.08E-09 | A | C | 0.0349 | 0.0283 | 0.2168 | 9 | 2983774 | 34.03 |
| rs1409379 | T | C | 0.0249 | 0.0041 | 1.67E-09 | T | C | 0.0207 | 0.0289 | 0.4735 | 13 | 31907741 | 36.88 |
| rs141954845 | A | G | 0.0229 | 0.0037 | 8.15E-10 | A | G | -0.0183 | 0.0271 | 0.5 | 3 | 61193647 | 38.31 |
| rs143186028 | T | G | 0.0277 | 0.0046 | 2.29E-09 | T | G | 0.055 | 0.031 | 0.07595 | 20 | 39997404 | 36.26 |
| rs1448938 | A | G | 0.0214 | 0.0035 | 1.3E-09 | A | G | 0.0351 | 0.0268 | 0.189 | 11 | 30892824 | 37.38 |
| rs1466887 | T | C | -0.0199 | 0.0036 | 4.12E-08 | T | C | 0.015 | 0.0272 | 0.5811 | 1 | 37709328 | 30.56 |
| rs1568452 | T | C | 0.0248 | 0.0036 | 8.12E-12 | T | C | 0.0422 | 0.0282 | 0.1348 | 2 | 58012833 | 47.46 |
| rs16887442 | T | C | 0.0203 | 0.0035 | 8.62E-09 | T | C | -0.033 | 0.0269 | 0.221 | 7 | 82936909 | 33.64 |
| rs169235 | A | G | -0.0229 | 0.0041 | 2.98E-08 | A | G | -0.0517 | 0.0312 | 0.09751 | 1 | 181740924 | 31.20 |
| rs17641524 | T | C | -0.032 | 0.0043 | 1.52E-13 | T | C | -0.0082 | 0.0382 | 0.8304 | 1 | 197704717 | 55.38 |
| rs1890946 | T | C | -0.0235 | 0.0035 | 2.68E-11 | T | C | -0.017 | 0.0268 | 0.5254 | 1 | 52342427 | 45.08 |
| rs1956373 | T | G | -0.0226 | 0.004 | 2.06E-08 | T | G | -0.0178 | 0.0345 | 0.6055 | 14 | 60141822 | 31.92 |
| rs1982277 | T | C | 0.0279 | 0.0041 | 1.45E-11 | T | C | 0.0027 | 0.0342 | 0.9362 | 9 | 11513019 | 46.31 |
| rs198457 | T | C | -0.0292 | 0.0046 | 2.99E-10 | T | C | -0.005 | 0.0449 | 0.9112 | 11 | 61471678 | 40.29 |
| rs200949 | A | G | 0.048 | 0.0053 | 2.53E-19 | A | G | 0.2066 | 0.0578 | 0.000353 | 6 | 27835435 | 82.02 |
| rs2043539 | A | G | 0.0273 | 0.0035 | 9.89E-15 | A | G | -0.0104 | 0.0278 | 0.708699 | 7 | 12253880 | 60.84 |
| rs2187490 | T | G | -0.0338 | 0.0061 | 3.82E-08 | T | G | 0.0458 | 0.0489 | 0.349 | 11 | 118713180 | 30.70 |
| rs2509805 | T | C | 0.022 | 0.0038 | 9.17E-09 | T | C | 0.0106 | 0.0278 | 0.7038 | 11 | 57650796 | 33.52 |
| rs2568958 | A | G | 0.0373 | 0.0036 | 8.47E-25 | A | G | -0.0123 | 0.0281 | 0.660999 | 1 | 72765116 | 107.35 |
| rs2670139 | T | C | -0.0266 | 0.0041 | 1.21E-10 | T | C | -0.0169 | 0.0278 | 0.5419 | 9 | 126634255 | 42.09 |
| rs301799 | T | C | -0.025 | 0.0035 | 1.36E-12 | T | C | -0.0116 | 0.0282 | 0.681 | 1 | 8489302 | 51.02 |
| rs30266 | A | G | 0.0308 | 0.0037 | 1.45E-16 | A | G | 0.0552 | 0.0297 | 0.06289 | 5 | 103972357 | 69.29 |
| rs3099439 | T | C | -0.0276 | 0.0035 | 5.05E-15 | T | C | -0.0366 | 0.027 | 0.1748 | 5 | 87545318 | 62.18 |
| rs3213572 | A | G | 0.0217 | 0.0035 | 7.61E-10 | A | G | 0.045 | 0.027 | 0.09619 | 12 | 121205078 | 38.44 |
| rs33431 | T | C | 0.0198 | 0.0036 | 4.81E-08 | T | C | 0.0491 | 0.028 | 0.079559 | 19 | 30939989 | 30.25 |
| rs34488670 | T | C | -0.0252 | 0.0043 | 6.03E-09 | T | C | -0.0252 | 0.0321 | 0.4327 | 15 | 47684936 | 34.35 |
| rs34937911 | T | C | 0.0304 | 0.0055 | 4.13E-08 | T | C | -0.0068 | 0.0424 | 0.8731 | 4 | 42110353 | 30.55 |
| rs35553410 | T | C | -0.0244 | 0.004 | 1.42E-09 | T | C | 0.0304 | 0.0312 | 0.3299 | 4 | 131239286 | 37.21 |
| rs3793577 | A | G | -0.0229 | 0.0035 | 8.41E-11 | A | G | -0.0159 | 0.027 | 0.555801 | 9 | 23737627 | 42.81 |
| rs3823624 | T | C | 0.0272 | 0.0045 | 1.99E-09 | T | C | 0.064 | 0.03 | 0.03287 | 7 | 2110346 | 36.54 |
| rs4346585 | T | C | -0.0236 | 0.0038 | 7.13E-10 | T | C | -0.0187 | 0.0283 | 0.5093 | 3 | 44736493 | 38.57 |
| rs45510091 | A | G | 0.0448 | 0.008 | 1.83E-08 | A | G | 0.0417 | 0.0624 | 0.5038 | 4 | 123186393 | 31.36 |
| rs4772087 | T | C | 0.0227 | 0.0036 | 3.91E-10 | T | C | 0.0538 | 0.0273 | 0.04889 | 13 | 99115041 | 39.76 |
| rs56314503 | T | G | -0.0254 | 0.004 | 2.95E-10 | T | G | 0.0299 | 0.0324 | 0.3571 | 12 | 84465022 | 40.32 |
| rs56887639 | A | G | -0.0278 | 0.0039 | 1.51E-12 | A | G | -0.0482 | 0.0287 | 0.093571 | 16 | 13755530 | 50.81 |
| rs57344483 | A | G | -0.038 | 0.0068 | 1.82E-08 | A | G | 0.0369 | 0.0403 | 0.3594 | 11 | 127022560 | 31.23 |
| rs58104186 | A | G | 0.0237 | 0.0035 | 1.82E-11 | A | G | -0.0249 | 0.027 | 0.3556 | 7 | 109099919 | 45.85 |
| rs59283172 | A | G | -0.0329 | 0.0057 | 1.02E-08 | A | G | -0.0414 | 0.0395 | 0.2943 | 9 | 25234672 | 33.32 |
| rs5995992 | T | C | -0.0266 | 0.0039 | 1.3E-11 | T | C | 0.0223 | 0.0288 | 0.4383 | 22 | 41487218 | 46.52 |
| rs60157091 | T | C | 0.02 | 0.0035 | 1.42E-08 | T | C | -0.0126 | 0.0269 | 0.6382 | 5 | 61509655 | 32.65 |
| rs61902811 | A | G | -0.0257 | 0.0036 | 1.4E-12 | A | G | 0.0015 | 0.0333 | 0.9649 | 11 | 113370758 | 50.96 |
| rs61990288 | A | G | -0.026 | 0.0035 | 1.68E-13 | A | G | 0.0079 | 0.0268 | 0.7673 | 14 | 42074726 | 55.18 |
| rs62091461 | T | C | -0.0254 | 0.0042 | 1.95E-09 | T | C | -0.0357 | 0.0285 | 0.2116 | 18 | 52488672 | 36.57 |
| rs62188629 | A | G | 0.0236 | 0.0038 | 7.13E-10 | A | G | 0.0062 | 0.0279 | 0.8232 | 2 | 208059440 | 38.57 |
| rs6783233 | T | C | 0.0218 | 0.0039 | 2.9E-8 | T | C | -0.0008 | 0.0328 | 0.9798 | 3 | 117509984 | 31.25 |
| rs7030813 | T | C | 0.0253 | 0.0036 | 3.07E-12 | T | C | 0.0199 | 0.0269 | 0.459 | 9 | 36999369 | 49.39 |
| rs7117514 | A | G | -0.0204 | 0.0035 | 7.29E-09 | A | G | 0.0233 | 0.027 | 0.3899 | 11 | 70544937 | 33.97 |
| rs7193263 | A | G | -0.0239 | 0.0038 | 4.33E-10 | A | G | 0.0211 | 0.0279 | 0.4499 | 16 | 6315880 | 39.56 |
| rs7198928 | T | C | 0.0239 | 0.0036 | 4.45E-11 | T | C | 0.0249 | 0.0287 | 0.3869 | 16 | 7666402 | 44.07 |
| rs7200826 | T | C | 0.028 | 0.004 | 3.74E-12 | T | C | -0.0188 | 0.0284 | 0.5088 | 16 | 13066833 | 49.00 |
| rs7227069 | A | G | 0.0238 | 0.0035 | 1.5E-11 | A | G | 0.0078 | 0.0272 | 0.7732 | 18 | 50731802 | 46.24 |
| rs7241572 | A | G | 0.028 | 0.0044 | 2.7E-10 | A | G | -0.0325 | 0.0327 | 0.3205 | 18 | 77580712 | 40.50 |
| rs725616 | T | C | 0.0204 | 0.0036 | 1.87E-08 | T | C | 0.0076 | 0.0274 | 0.781399 | 6 | 147950422 | 32.11 |
| rs72710803 | A | C | -0.041 | 0.0062 | 5.29E-11 | A | C | -0.0512 | 0.078 | 0.512 | 1 | 177428018 | 43.73 |
| rs75581564 | A | G | 0.0301 | 0.0054 | 3.17E-08 | A | G | 0.0633 | 0.0394 | 0.1081 | 17 | 27363750 | 31.07 |
| rs7585722 | T | C | -0.0269 | 0.0048 | 2.68E-08 | T | C | -0.0401 | 0.0465 | 0.3883 | 2 | 86819128 | 31.41 |
| rs7624336 | T | G | 0.0238 | 0.0043 | 3.96E-08 | T | G | 0.0068 | 0.0363 | 0.8508 | 3 | 53244151 | 30.63 |
| rs7659414 | A | C | -0.0201 | 0.0035 | 1.2E-8 | A | C | -0.0157 | 0.0279 | 0.574399 | 4 | 177350956 | 32.98 |
| rs7685686 | A | G | 0.0202 | 0.0036 | 2.57E-08 | A | G | 0.0134 | 0.027 | 0.619001 | 4 | 3207142 | 31.48 |
| rs7807677 | T | C | 0.0237 | 0.0035 | 1.82E-11 | T | C | -0.0378 | 0.0277 | 0.1733 | 7 | 117502574 | 45.85 |
| rs78337797 | T | G | 0.0306 | 0.0055 | 3.37E-08 | T | G | 0.0246 | 0.0454 | 0.5887 | 12 | 23987925 | 30.95 |
| rs7837935 | T | G | -0.0292 | 0.0049 | 3.34E-09 | T | G | -0.1071 | 0.0414 | 0.009761 | 8 | 65562019 | 35.51 |
| rs7932640 | T | C | 0.0281 | 0.0035 | 1.62E-15 | T | C | -0.0244 | 0.0269 | 0.3639 | 11 | 88744425 | 64.46 |
| rs8037355 | T | C | -0.0233 | 0.0035 | 3.94E-11 | T | C | -0.0073 | 0.0269 | 0.7865 | 15 | 37643831 | 44.32 |
| rs913930 | A | G | -0.0208 | 0.0037 | 2.42E-08 | A | G | -0.0022 | 0.0276 | 0.9363 | 9 | 120484009 | 31.60 |
| rs9363467 | T | C | 0.0237 | 0.0036 | 6.44E-11 | T | C | -0.0101 | 0.0278 | 0.715599 | 6 | 66605257 | 43.34 |
| rs9545360 | A | C | -0.0271 | 0.0046 | 5.02E-09 | A | C | -0.0245 | 0.038 | 0.519501 | 13 | 80870712 | 34.71 |
| rs9592461 | A | G | 0.0216 | 0.0035 | 9.1E-10 | A | G | -0.0088 | 0.027 | 0.7438 | 13 | 66941792 | 38.09 |
| rs997934 | T | C | 0.0198 | 0.0036 | 4.81E-08 | T | C | -0.0286 | 0.027 | 0.2896 | 10 | 1795194 | 30.25 |
|  |  |  |  |  |  |  |  |  |  |  |  |  |  |
| SNP | Effects on depression | | | | | Effects on AS | | | | | Chr | Position | F |
|  | EA | OA | Beta | SE | p-val | EA | OA | Beta | SE | p-val |  |  | statistic |
| rs1002656 | T | C | -0.0266 | 0.0038 | 3.74E-12 | T | C | 0.0000402 | 0.000121 | 0.74 | 1 | 37192741 | 49.00 |
| rs10149470 | A | G | -0.0267 | 0.0035 | 3.72E-14 | A | G | 0.000161 | 0.00011 | 0.14 | 14 | 104017953 | 58.20 |
| rs1021363 | A | G | 0.0303 | 0.0037 | 4.41E-16 | A | G | 0.00002 | 0.000115 | 0.86 | 10 | 106610839 | 67.06 |
| rs1045430 | T | G | -0.0253 | 0.0035 | 7.31E-13 | T | G | -0.000032 | 0.00011 | 0.77 | 14 | 75130235 | 52.25 |
| rs10789214 | T | C | 0.0193 | 0.0035 | 4.44E-08 | T | C | 0.000123 | 0.000111 | 0.27 | 1 | 67146817 | 30.41 |
| rs10817969 | T | G | 0.0261 | 0.0039 | 3.11E-11 | T | G | 0.000144 | 0.000122 | 0.24 | 9 | 119731045 | 44.79 |
| rs10890020 | A | G | -0.0277 | 0.0035 | 4.03E-15 | A | G | -0.000035 | 0.00011 | 0.75 | 1 | 73668836 | 62.64 |
| rs10913112 | T | C | -0.0264 | 0.0036 | 3.4E-13 | T | C | -0.000067 | 0.000113 | 0.56 | 1 | 175913828 | 53.78 |
| rs1095626 | T | C | -0.0264 | 0.0035 | 7.13E-14 | T | C | 0.000193 | 0.000111 | 0.083 | 3 | 157977962 | 56.89 |
| rs11135349 | A | C | -0.0295 | 0.0035 | 6.04E-17 | A | C | -0.000069 | 0.00011 | 0.53 | 5 | 164523472 | 71.04 |
| rs113188507 | A | G | 0.0221 | 0.0039 | 1.87E-08 | A | G | -0.00021 | 0.00012 | 0.075999 | 1 | 80809636 | 32.11 |
| rs1152578 | T | C | -0.0218 | 0.0035 | 6.36E-10 | T | C | 0.00019 | 0.000111 | 0.086 | 14 | 64697037 | 38.80 |
| rs12923444 | A | C | -0.0214 | 0.0035 | 1.3E-09 | A | C | -0.000044 | 0.000111 | 0.69 | 16 | 21639710 | 37.38 |
| rs12967855 | A | G | 0.0265 | 0.0037 | 1.18E-12 | A | G | 0.0000742 | 0.000117 | 0.53 | 18 | 35138245 | 51.30 |
| rs1343605 | A | C | 0.0313 | 0.0036 | 6.23E-18 | A | C | -0.00011 | 0.000114 | 0.32 | 13 | 53647048 | 75.59 |
| rs1354115 | A | C | 0.021 | 0.0036 | 7.08E-09 | A | C | -0.000023 | 0.000114 | 0.84 | 9 | 2983774 | 34.03 |
| rs1409379 | T | C | 0.0249 | 0.0041 | 1.67E-09 | T | C | 0.0000414 | 0.000124 | 0.74 | 13 | 31910189 | 36.88 |
| rs141954845 | A | G | 0.0229 | 0.0037 | 8.15E-10 | A | G | -0.00021 | 0.000112 | 0.056 | 3 | 61196910 | 38.31 |
| rs1448938 | A | G | 0.0214 | 0.0035 | 1.3E-09 | A | G | -0.000035 | 0.000111 | 0.760001 | 11 | 30892824 | 37.38 |
| rs1466887 | T | C | -0.0199 | 0.0036 | 4.12E-08 | T | C | -0.000026 | 0.000111 | 0.81 | 1 | 37709328 | 30.56 |
| rs1568452 | T | C | 0.0248 | 0.0036 | 8.12E-12 | T | C | 0.000133 | 0.000113 | 0.24 | 2 | 58012833 | 47.46 |
| rs16887442 | T | C | 0.0203 | 0.0035 | 8.62E-09 | T | C | -0.00012 | 0.000111 | 0.29 | 7 | 82936909 | 33.64 |
| rs1890946 | T | C | -0.0235 | 0.0035 | 2.68E-11 | T | C | -0.000069 | 0.00011 | 0.53 | 1 | 52342427 | 45.08 |
| rs2043539 | A | G | 0.0273 | 0.0035 | 9.89E-15 | A | G | 0.000116 | 0.000111 | 0.3 | 7 | 12253880 | 60.84 |
| rs2509805 | T | C | 0.022 | 0.0038 | 9.17E-09 | T | C | -0.000095 | 0.000118 | 0.42 | 11 | 57650796 | 33.52 |
| rs2568958 | A | G | 0.0373 | 0.0036 | 8.47E-25 | A | G | 0.000129 | 0.000112 | 0.25 | 1 | 72765116 | 107.35 |
| rs301799 | T | C | -0.025 | 0.0035 | 1.36E-12 | T | C | 0.00000189 | 0.000111 | 0.99 | 1 | 8489302 | 51.02 |
| rs30266 | A | G | 0.0308 | 0.0037 | 1.45E-16 | A | G | -0.0002 | 0.000117 | 0.083 | 5 | 103972357 | 69.29 |
| rs3099439 | T | C | -0.0276 | 0.0035 | 5.05E-15 | T | C | -0.00000031 | 0.000111 | 1 | 5 | 87545318 | 62.18 |
| rs3213572 | A | G | 0.0217 | 0.0035 | 7.61E-10 | A | G | 0.00000486 | 0.00011 | 0.96 | 12 | 121205078 | 38.44 |
| rs33431 | T | C | 0.0198 | 0.0036 | 4.81E-08 | T | C | 0.00000574 | 0.000114 | 0.96 | 19 | 30939989 | 30.25 |
| rs35553410 | T | C | -0.0244 | 0.004 | 1.42E-09 | T | C | -0.00009 | 0.000122 | 0.46 | 4 | 131238051 | 37.21 |
| rs3793577 | A | G | -0.0229 | 0.0035 | 8.41E-11 | A | G | -0.00004 | 0.000111 | 0.719999 | 9 | 23737627 | 42.81 |
| rs4346585 | T | C | -0.0236 | 0.0038 | 7.13E-10 | T | C | -0.00017 | 0.000118 | 0.14 | 3 | 44906746 | 38.57 |
| rs4772087 | T | C | 0.0227 | 0.0036 | 3.91E-10 | T | C | 0.0000393 | 0.000114 | 0.73 | 13 | 99115041 | 39.76 |
| rs58104186 | A | G | 0.0237 | 0.0035 | 1.82E-11 | A | G | 0.000107 | 0.000111 | 0.33 | 7 | 109099919 | 45.85 |
| rs5995992 | T | C | -0.0266 | 0.0039 | 1.3E-11 | T | C | 0.000142 | 0.000121 | 0.24 | 22 | 41487218 | 46.52 |
| rs60157091 | T | C | 0.02 | 0.0035 | 1.42E-08 | T | C | 0.000094 | 0.00011 | 0.39 | 5 | 61509655 | 32.65 |
| rs61902811 | A | G | -0.0257 | 0.0036 | 1.4E-12 | A | G | -0.000087 | 0.000114 | 0.44 | 11 | 113370758 | 50.96 |
| rs61990288 | A | G | -0.026 | 0.0035 | 1.68E-13 | A | G | -0.00017 | 0.00011 | 0.12 | 14 | 42074726 | 55.18 |
| rs62188629 | A | G | 0.0236 | 0.0038 | 7.13E-10 | A | G | -0.000058 | 0.000119 | 0.630001 | 2 | 208044470 | 38.57 |
| rs6783233 | T | C | 0.0218 | 0.0039 | 2.9E-8 | T | C | 0.0000712 | 0.000122 | 0.56 | 3 | 117509984 | 31.25 |
| rs7030813 | T | C | 0.0253 | 0.0036 | 3.07E-12 | T | C | 0.000208 | 0.000114 | 0.068 | 9 | 36999369 | 49.39 |
| rs7117514 | A | G | -0.0204 | 0.0035 | 7.29E-09 | A | G | 0.0000239 | 0.00011 | 0.83 | 11 | 70544937 | 33.97 |
| rs7193263 | A | G | -0.0239 | 0.0038 | 4.33E-10 | A | G | 0.000206 | 0.000118 | 0.081001 | 16 | 6315880 | 39.56 |
| rs7198928 | T | C | 0.0239 | 0.0036 | 4.45E-11 | T | C | 0.000129 | 0.000114 | 0.26 | 16 | 7666402 | 44.07 |
| rs7200826 | T | C | 0.028 | 0.004 | 3.74E-12 | T | C | 0.0000389 | 0.000124 | 0.75 | 16 | 13101618 | 49.00 |
| rs7227069 | A | G | 0.0238 | 0.0035 | 1.5E-11 | A | G | 0.0000689 | 0.000111 | 0.54 | 18 | 50731802 | 46.24 |
| rs725616 | T | C | 0.0204 | 0.0036 | 1.87E-08 | T | C | -0.000031 | 0.000114 | 0.79 | 6 | 147950422 | 32.11 |
| rs7659414 | A | C | -0.0201 | 0.0035 | 1.2E-8 | A | C | 0.000033 | 0.000111 | 0.77 | 4 | 177350956 | 32.98 |
| rs7685686 | A | G | 0.0202 | 0.0036 | 2.57E-08 | A | G | 0.0000496 | 0.000111 | 0.66 | 4 | 3207142 | 31.48 |
| rs7807677 | T | C | 0.0237 | 0.0035 | 1.82E-11 | T | C | -0.000053 | 0.00011 | 0.630001 | 7 | 117502574 | 45.85 |
| rs7932640 | T | C | 0.0281 | 0.0035 | 1.62E-15 | T | C | 0.00000349 | 0.000111 | 0.97 | 11 | 88744425 | 64.46 |
| rs8037355 | T | C | -0.0233 | 0.0035 | 3.94E-11 | T | C | 0.0000248 | 0.00011 | 0.82 | 15 | 37643831 | 44.32 |
| rs913930 | A | G | -0.0208 | 0.0037 | 2.42E-08 | A | G | -0.00012 | 0.000116 | 0.29 | 9 | 120484009 | 31.60 |
| rs9363467 | T | C | 0.0237 | 0.0036 | 6.44E-11 | T | C | -0.00022 | 0.000113 | 0.052 | 6 | 66605257 | 43.34 |
| rs9592461 | A | G | 0.0216 | 0.0035 | 9.1E-10 | A | G | -0.000095 | 0.00011 | 0.39 | 13 | 66941792 | 38.09 |
| rs997934 | T | C | 0.0198 | 0.0036 | 4.81E-08 | T | C | 0.000116 | 0.000113 | 0.31 | 10 | 1795194 | 30.25 |
|  |  |  |  |  |  |  |  |  |  |  |  |  |  |
| SNP | Effects on depression | | | | | Effects on Seronegative RA | | | | | Chr | Position | F |
|  | EA | OA | Beta | SE | p-val | EA | OA | Beta | SE | p-val |  |  | statistic |
| rs1002656 | T | C | -0.0266 | 0.0038 | 3.74E-12 | T | C | -0.0461 | 0.0357 | 0.1967 | 1 | 37192741 | 49.00 |
| rs10149470 | A | G | -0.0267 | 0.0035 | 3.72E-14 | A | G | -0.0024 | 0.0333 | 0.9432 | 14 | 104017953 | 58.20 |
| rs1021363 | A | G | 0.0303 | 0.0037 | 4.41E-16 | A | G | 0.0209 | 0.0371 | 0.5737 | 10 | 106610839 | 67.06 |
| rs1045430 | T | G | -0.0253 | 0.0035 | 7.31E-13 | T | G | -0.0434 | 0.0331 | 0.1895 | 14 | 75130235 | 52.25 |
| rs10774600 | T | C | -0.0267 | 0.0048 | 3.39E-08 | T | C | 0.0492 | 0.0508 | 0.3331 | 12 | 110741356 | 30.94 |
| rs10789214 | T | C | 0.0193 | 0.0035 | 4.44E-08 | T | C | -0.0116 | 0.0334 | 0.729001 | 1 | 67146817 | 30.41 |
| rs10817969 | T | G | 0.0261 | 0.0039 | 3.11E-11 | T | G | -0.0319 | 0.0356 | 0.3691 | 9 | 119731045 | 44.79 |
| rs10890020 | A | G | -0.0277 | 0.0035 | 4.03E-15 | A | G | 0.0075 | 0.0332 | 0.8209 | 1 | 73669845 | 62.64 |
| rs10913112 | T | C | -0.0264 | 0.0036 | 3.4E-13 | T | C | -0.0264 | 0.0343 | 0.4406 | 1 | 175913828 | 53.78 |
| rs1095626 | T | C | -0.0264 | 0.0035 | 7.13E-14 | T | C | 0.0059 | 0.0332 | 0.8592 | 3 | 157977962 | 56.89 |
| rs11135349 | A | C | -0.0295 | 0.0035 | 6.04E-17 | A | C | -0.0011 | 0.0339 | 0.9731 | 5 | 164523472 | 71.04 |
| rs113188507 | A | G | 0.0221 | 0.0039 | 1.87E-08 | A | G | -0.0444 | 0.0416 | 0.2852 | 1 | 80809636 | 32.11 |
| rs1152578 | T | C | -0.0218 | 0.0035 | 6.36E-10 | T | C | 0.0146 | 0.0335 | 0.6631 | 14 | 64697037 | 38.80 |
| rs11579246 | A | G | 0.0381 | 0.0061 | 5.71E-10 | A | G | 0.0221 | 0.0459 | 0.6304 | 1 | 50559162 | 39.01 |
| rs1226412 | T | C | 0.0256 | 0.0043 | 3.46E-09 | T | C | 0.0344 | 0.0384 | 0.37 | 2 | 157111313 | 35.44 |
| rs12624433 | A | G | 0.0233 | 0.004 | 7.44E-09 | A | G | -0.042 | 0.0375 | 0.2637 | 20 | 44680853 | 33.93 |
| rs12923444 | A | C | -0.0214 | 0.0035 | 1.3E-09 | A | C | -0.047 | 0.0341 | 0.1686 | 16 | 21639710 | 37.38 |
| rs12967855 | A | G | 0.0265 | 0.0037 | 1.18E-12 | A | G | 0.0271 | 0.0375 | 0.4701 | 18 | 35138245 | 51.30 |
| rs13084037 | A | G | -0.0245 | 0.0042 | 7.08E-09 | A | G | -0.0402 | 0.0451 | 0.3734 | 3 | 49214066 | 34.03 |
| rs1343605 | A | C | 0.0313 | 0.0036 | 6.23E-18 | A | C | 0.0287 | 0.0342 | 0.4006 | 13 | 53647048 | 75.59 |
| rs1354115 | A | C | 0.021 | 0.0036 | 7.08E-09 | A | C | 0.0506 | 0.0349 | 0.1469 | 9 | 2983774 | 34.03 |
| rs1409379 | T | C | 0.0249 | 0.0041 | 1.67E-09 | T | C | 0.0163 | 0.0357 | 0.6484 | 13 | 31907741 | 36.88 |
| rs141954845 | A | G | 0.0229 | 0.0037 | 8.15E-10 | A | G | -0.0576 | 0.0334 | 0.08446 | 3 | 61193647 | 38.31 |
| rs143186028 | T | G | 0.0277 | 0.0046 | 2.29E-09 | T | G | -0.0219 | 0.0383 | 0.567799 | 20 | 39997404 | 36.26 |
| rs1448938 | A | G | 0.0214 | 0.0035 | 1.3E-09 | A | G | -0.0368 | 0.033 | 0.2651 | 11 | 30892824 | 37.38 |
| rs1466887 | T | C | -0.0199 | 0.0036 | 4.12E-08 | T | C | -0.0337 | 0.0336 | 0.3161 | 1 | 37709328 | 30.56 |
| rs1568452 | T | C | 0.0248 | 0.0036 | 8.12E-12 | T | C | -0.0045 | 0.0348 | 0.8975 | 2 | 58012833 | 47.46 |
| rs16887442 | T | C | 0.0203 | 0.0035 | 8.62E-09 | T | C | -0.0484 | 0.0333 | 0.1457 | 7 | 82936909 | 33.64 |
| rs169235 | A | G | -0.0229 | 0.0041 | 2.98E-08 | A | G | -0.0648 | 0.0385 | 0.092711 | 1 | 181740924 | 31.20 |
| rs17641524 | T | C | -0.032 | 0.0043 | 1.52E-13 | T | C | 0.0814 | 0.0468 | 0.081941 | 1 | 197704717 | 55.38 |
| rs1890946 | T | C | -0.0235 | 0.0035 | 2.68E-11 | T | C | -0.0173 | 0.0331 | 0.6026 | 1 | 52342427 | 45.08 |
| rs1956373 | T | G | -0.0226 | 0.004 | 2.06E-08 | T | G | -0.0028 | 0.0423 | 0.9473 | 14 | 60141822 | 31.92 |
| rs1982277 | T | C | 0.0279 | 0.0041 | 1.45E-11 | T | C | -0.0047 | 0.0421 | 0.9109 | 9 | 11513019 | 46.31 |
| rs198457 | T | C | -0.0292 | 0.0046 | 2.99E-10 | T | C | 0.022 | 0.0557 | 0.6931 | 11 | 61471678 | 40.29 |
| rs200949 | A | G | 0.048 | 0.0053 | 2.53E-19 | A | G | -0.1319 | 0.0702 | 0.06051 | 6 | 27835435 | 82.02 |
| rs2043539 | A | G | 0.0273 | 0.0035 | 9.89E-15 | A | G | -0.0109 | 0.0342 | 0.749899 | 7 | 12253880 | 60.84 |
| rs2187490 | T | G | -0.0338 | 0.0061 | 3.82E-08 | T | G | -0.0017 | 0.0609 | 0.9778 | 11 | 118713180 | 30.70 |
| rs2509805 | T | C | 0.022 | 0.0038 | 9.17E-09 | T | C | -0.0091 | 0.0344 | 0.7921 | 11 | 57650796 | 33.52 |
| rs2568958 | A | G | 0.0373 | 0.0036 | 8.47E-25 | A | G | -0.0046 | 0.0346 | 0.8941 | 1 | 72765116 | 107.35 |
| rs2670139 | T | C | -0.0266 | 0.0041 | 1.21E-10 | T | C | -0.0458 | 0.0343 | 0.1819 | 9 | 126634255 | 42.09 |
| rs301799 | T | C | -0.025 | 0.0035 | 1.36E-12 | T | C | 0.0388 | 0.0348 | 0.2646 | 1 | 8489302 | 51.02 |
| rs30266 | A | G | 0.0308 | 0.0037 | 1.45E-16 | A | G | 0.0518 | 0.0365 | 0.1556 | 5 | 103972357 | 69.29 |
| rs3099439 | T | C | -0.0276 | 0.0035 | 5.05E-15 | T | C | -0.0349 | 0.0333 | 0.2944 | 5 | 87545318 | 62.18 |
| rs3213572 | A | G | 0.0217 | 0.0035 | 7.61E-10 | A | G | -0.0332 | 0.0334 | 0.3196 | 12 | 121205078 | 38.44 |
| rs33431 | T | C | 0.0198 | 0.0036 | 4.81E-08 | T | C | 0.0365 | 0.0345 | 0.2899 | 19 | 30939989 | 30.25 |
| rs34488670 | T | C | -0.0252 | 0.0043 | 6.03E-09 | T | C | -0.0574 | 0.0396 | 0.1474 | 15 | 47684936 | 34.35 |
| rs34937911 | T | C | 0.0304 | 0.0055 | 4.13E-08 | T | C | -0.0294 | 0.0526 | 0.576301 | 4 | 42110353 | 30.55 |
| rs35553410 | T | C | -0.0244 | 0.004 | 1.42E-09 | T | C | -0.0296 | 0.0384 | 0.441199 | 4 | 131239286 | 37.21 |
| rs3793577 | A | G | -0.0229 | 0.0035 | 8.41E-11 | A | G | -0.0186 | 0.0334 | 0.577301 | 9 | 23737627 | 42.81 |
| rs3823624 | T | C | 0.0272 | 0.0045 | 1.99E-09 | T | C | 0.0203 | 0.0371 | 0.5854 | 7 | 2110346 | 36.54 |
| rs4346585 | T | C | -0.0236 | 0.0038 | 7.13E-10 | T | C | 0.0377 | 0.035 | 0.2822 | 3 | 44736493 | 38.57 |
| rs45510091 | A | G | 0.0448 | 0.008 | 1.83E-08 | A | G | 0.0139 | 0.0763 | 0.8556 | 4 | 123186393 | 31.36 |
| rs4772087 | T | C | 0.0227 | 0.0036 | 3.91E-10 | T | C | -0.0103 | 0.0338 | 0.759299 | 13 | 99115041 | 39.76 |
| rs56314503 | T | G | -0.0254 | 0.004 | 2.95E-10 | T | G | 0.0019 | 0.0399 | 0.9618 | 12 | 84465022 | 40.32 |
| rs56887639 | A | G | -0.0278 | 0.0039 | 1.51E-12 | A | G | -0.0242 | 0.0355 | 0.4959 | 16 | 13755530 | 50.81 |
| rs57344483 | A | G | -0.038 | 0.0068 | 1.82E-08 | A | G | -0.0883 | 0.0498 | 0.07653 | 11 | 127022560 | 31.23 |
| rs58104186 | A | G | 0.0237 | 0.0035 | 1.82E-11 | A | G | -0.0147 | 0.0333 | 0.659601 | 7 | 109099919 | 45.85 |
| rs59283172 | A | G | -0.0329 | 0.0057 | 1.02E-08 | A | G | -0.0352 | 0.0486 | 0.4686 | 9 | 25234672 | 33.32 |
| rs5995992 | T | C | -0.0266 | 0.0039 | 1.3E-11 | T | C | 0.0713 | 0.0356 | 0.04493 | 22 | 41487218 | 46.52 |
| rs60157091 | T | C | 0.02 | 0.0035 | 1.42E-08 | T | C | 0.0065 | 0.0332 | 0.8446 | 5 | 61509655 | 32.65 |
| rs61902811 | A | G | -0.0257 | 0.0036 | 1.4E-12 | A | G | -0.034 | 0.0407 | 0.4025 | 11 | 113370758 | 50.96 |
| rs61990288 | A | G | -0.026 | 0.0035 | 1.68E-13 | A | G | 0.0172 | 0.0331 | 0.6033 | 14 | 42074726 | 55.18 |
| rs62091461 | T | C | -0.0254 | 0.0042 | 1.95E-09 | T | C | 0.0253 | 0.0352 | 0.4731 | 18 | 52488672 | 36.57 |
| rs62188629 | A | G | 0.0236 | 0.0038 | 7.13E-10 | A | G | 0.0422 | 0.0344 | 0.2203 | 2 | 208059440 | 38.57 |
| rs6783233 | T | C | 0.0218 | 0.0039 | 2.9E-8 | T | C | 0.0016 | 0.0404 | 0.9674 | 3 | 117509984 | 31.25 |
| rs7030813 | T | C | 0.0253 | 0.0036 | 3.07E-12 | T | C | -0.0179 | 0.0332 | 0.5892 | 9 | 36999369 | 49.39 |
| rs7117514 | A | G | -0.0204 | 0.0035 | 7.29E-09 | A | G | -0.0277 | 0.0334 | 0.4064 | 11 | 70544937 | 33.97 |
| rs7193263 | A | G | -0.0239 | 0.0038 | 4.33E-10 | A | G | -0.0049 | 0.0344 | 0.8856 | 16 | 6315880 | 39.56 |
| rs7198928 | T | C | 0.0239 | 0.0036 | 4.45E-11 | T | C | 0.0001 | 0.0355 | 0.9979 | 16 | 7666402 | 44.07 |
| rs7200826 | T | C | 0.028 | 0.004 | 3.74E-12 | T | C | -0.0158 | 0.0351 | 0.6519 | 16 | 13066833 | 49.00 |
| rs7227069 | A | G | 0.0238 | 0.0035 | 1.5E-11 | A | G | -0.0062 | 0.0336 | 0.8537 | 18 | 50731802 | 46.24 |
| rs7241572 | A | G | 0.028 | 0.0044 | 2.7E-10 | A | G | 0.0128 | 0.0404 | 0.7511 | 18 | 77580712 | 40.50 |
| rs725616 | T | C | 0.0204 | 0.0036 | 1.87E-08 | T | C | -0.0064 | 0.0339 | 0.8504 | 6 | 147950422 | 32.11 |
| rs72710803 | A | C | -0.041 | 0.0062 | 5.29E-11 | A | C | -0.0997 | 0.0952 | 0.2946 | 1 | 177428018 | 43.73 |
| rs75581564 | A | G | 0.0301 | 0.0054 | 3.17E-08 | A | G | 0.012 | 0.0488 | 0.8049 | 17 | 27363750 | 31.07 |
| rs7585722 | T | C | -0.0269 | 0.0048 | 2.68E-08 | T | C | -0.0689 | 0.0573 | 0.2294 | 2 | 86819128 | 31.41 |
| rs7624336 | T | G | 0.0238 | 0.0043 | 3.96E-08 | T | G | -0.021 | 0.0448 | 0.639401 | 3 | 53244151 | 30.63 |
| rs7659414 | A | C | -0.0201 | 0.0035 | 1.2E-8 | A | C | 0.0192 | 0.0345 | 0.5784 | 4 | 177350956 | 32.98 |
| rs7685686 | A | G | 0.0202 | 0.0036 | 2.57E-08 | A | G | 0.0146 | 0.0333 | 0.6601 | 4 | 3207142 | 31.48 |
| rs7807677 | T | C | 0.0237 | 0.0035 | 1.82E-11 | T | C | -0.0192 | 0.0342 | 0.575 | 7 | 117502574 | 45.85 |
| rs78337797 | T | G | 0.0306 | 0.0055 | 3.37E-08 | T | G | -0.0009 | 0.0559 | 0.9874 | 12 | 23987925 | 30.95 |
| rs7837935 | T | G | -0.0292 | 0.0049 | 3.34E-09 | T | G | 0.0539 | 0.0506 | 0.287 | 8 | 65562019 | 35.51 |
| rs7932640 | T | C | 0.0281 | 0.0035 | 1.62E-15 | T | C | 0.0166 | 0.0332 | 0.617401 | 11 | 88744425 | 64.46 |
| rs8037355 | T | C | -0.0233 | 0.0035 | 3.94E-11 | T | C | 0.0156 | 0.0332 | 0.6383 | 15 | 37643831 | 44.32 |
| rs913930 | A | G | -0.0208 | 0.0037 | 2.42E-08 | A | G | 0.019 | 0.0341 | 0.5783 | 9 | 120484009 | 31.60 |
| rs9363467 | T | C | 0.0237 | 0.0036 | 6.44E-11 | T | C | -0.0308 | 0.0343 | 0.3685 | 6 | 66605257 | 43.34 |
| rs9545360 | A | C | -0.0271 | 0.0046 | 5.02E-09 | A | C | 0.0449 | 0.0467 | 0.3363 | 13 | 80870712 | 34.71 |
| rs9592461 | A | G | 0.0216 | 0.0035 | 9.1E-10 | A | G | 0.0217 | 0.0333 | 0.513701 | 13 | 66941792 | 38.09 |
| rs997934 | T | C | 0.0198 | 0.0036 | 4.81E-08 | T | C | 0.0091 | 0.0333 | 0.785599 | 10 | 1795194 | 30.25 |
|  |  |  |  |  |  |  |  |  |  |  |  |  |  |
| SNP | Effects on depression | | | | | Effects on Seropositive RA | | | | | Chr | Position | F |
|  | EA | OA | Beta | SE | p-val | EA | OA | Beta | SE | p-val |  |  | statistic |
| rs1002656 | T | C | -0.0266 | 0.0038 | 3.74E-12 | T | C | -0.0298 | 0.0432 | 0.4898 | 1 | 37192741 | 49.00 |
| rs10149470 | A | G | -0.0267 | 0.0035 | 3.72E-14 | A | G | 0.0076 | 0.0404 | 0.8501 | 14 | 104017953 | 58.20 |
| rs1021363 | A | G | 0.0303 | 0.0037 | 4.41E-16 | A | G | -0.0638 | 0.0451 | 0.1566 | 10 | 106610839 | 67.06 |
| rs1045430 | T | G | -0.0253 | 0.0035 | 7.31E-13 | T | G | -0.0348 | 0.0402 | 0.3859 | 14 | 75130235 | 52.25 |
| rs10774600 | T | C | -0.0267 | 0.0048 | 3.39E-08 | T | C | 0.0142 | 0.0619 | 0.8191 | 12 | 110741356 | 30.94 |
| rs10789214 | T | C | 0.0193 | 0.0035 | 4.44E-08 | T | C | 0.017 | 0.0405 | 0.675 | 1 | 67146817 | 30.41 |
| rs10817969 | T | G | 0.0261 | 0.0039 | 3.11E-11 | T | G | 0.0036 | 0.0431 | 0.9332 | 9 | 119731045 | 44.79 |
| rs10890020 | A | G | -0.0277 | 0.0035 | 4.03E-15 | A | G | 0.0207 | 0.0403 | 0.6069 | 1 | 73669845 | 62.64 |
| rs10913112 | T | C | -0.0264 | 0.0036 | 3.4E-13 | T | C | -0.0646 | 0.0415 | 0.1201 | 1 | 175913828 | 53.78 |
| rs1095626 | T | C | -0.0264 | 0.0035 | 7.13E-14 | T | C | -0.0628 | 0.0402 | 0.1185 | 3 | 157977962 | 56.89 |
| rs11135349 | A | C | -0.0295 | 0.0035 | 6.04E-17 | A | C | 0.0432 | 0.0412 | 0.2937 | 5 | 164523472 | 71.04 |
| rs113188507 | A | G | 0.0221 | 0.0039 | 1.87E-08 | A | G | 0.0467 | 0.0507 | 0.3572 | 1 | 80809636 | 32.11 |
| rs1152578 | T | C | -0.0218 | 0.0035 | 6.36E-10 | T | C | 0.0243 | 0.0406 | 0.549099 | 14 | 64697037 | 38.80 |
| rs11579246 | A | G | 0.0381 | 0.0061 | 5.71E-10 | A | G | -0.0088 | 0.0553 | 0.8739 | 1 | 50559162 | 39.01 |
| rs1226412 | T | C | 0.0256 | 0.0043 | 3.46E-09 | T | C | 0.051 | 0.0466 | 0.2741 | 2 | 157111313 | 35.44 |
| rs12624433 | A | G | 0.0233 | 0.004 | 7.44E-09 | A | G | -0.0662 | 0.0456 | 0.1466 | 20 | 44680853 | 33.93 |
| rs12923444 | A | C | -0.0214 | 0.0035 | 1.3E-09 | A | C | -0.0043 | 0.0413 | 0.917 | 16 | 21639710 | 37.38 |
| rs12967855 | A | G | 0.0265 | 0.0037 | 1.18E-12 | A | G | -0.0055 | 0.0456 | 0.9039 | 18 | 35138245 | 51.30 |
| rs13084037 | A | G | -0.0245 | 0.0042 | 7.08E-09 | A | G | -0.1098 | 0.0552 | 0.04682 | 3 | 49214066 | 34.03 |
| rs1343605 | A | C | 0.0313 | 0.0036 | 6.23E-18 | A | C | 0.0132 | 0.0415 | 0.7504 | 13 | 53647048 | 75.59 |
| rs1354115 | A | C | 0.021 | 0.0036 | 7.08E-09 | A | C | 0.0079 | 0.0423 | 0.8515 | 9 | 2983774 | 34.03 |
| rs1409379 | T | C | 0.0249 | 0.0041 | 1.67E-09 | T | C | -0.0982 | 0.0432 | 0.023 | 13 | 31907741 | 36.88 |
| rs141954845 | A | G | 0.0229 | 0.0037 | 8.15E-10 | A | G | 0.0049 | 0.0406 | 0.9041 | 3 | 61193647 | 38.31 |
| rs143186028 | T | G | 0.0277 | 0.0046 | 2.29E-09 | T | G | 0.015 | 0.0464 | 0.7457 | 20 | 39997404 | 36.26 |
| rs1448938 | A | G | 0.0214 | 0.0035 | 1.3E-09 | A | G | -0.046 | 0.0401 | 0.2515 | 11 | 30892824 | 37.38 |
| rs1466887 | T | C | -0.0199 | 0.0036 | 4.12E-08 | T | C | -0.0199 | 0.0408 | 0.625801 | 1 | 37709328 | 30.56 |
| rs1568452 | T | C | 0.0248 | 0.0036 | 8.12E-12 | T | C | 0.0996 | 0.0422 | 0.01831 | 2 | 58012833 | 47.46 |
| rs16887442 | T | C | 0.0203 | 0.0035 | 8.62E-09 | T | C | 0.0359 | 0.0404 | 0.3735 | 7 | 82936909 | 33.64 |
| rs169235 | A | G | -0.0229 | 0.0041 | 2.98E-08 | A | G | 0.0246 | 0.0468 | 0.5997 | 1 | 181740924 | 31.20 |
| rs17641524 | T | C | -0.032 | 0.0043 | 1.52E-13 | T | C | 0.0423 | 0.0571 | 0.4585 | 1 | 197704717 | 55.38 |
| rs1890946 | T | C | -0.0235 | 0.0035 | 2.68E-11 | T | C | 0.0167 | 0.0402 | 0.6773 | 1 | 52342427 | 45.08 |
| rs1956373 | T | G | -0.0226 | 0.004 | 2.06E-08 | T | G | 0.029 | 0.052 | 0.577301 | 14 | 60141822 | 31.92 |
| rs1982277 | T | C | 0.0279 | 0.0041 | 1.45E-11 | T | C | 0.0125 | 0.0513 | 0.8071 | 9 | 11513019 | 46.31 |
| rs198457 | T | C | -0.0292 | 0.0046 | 2.99E-10 | T | C | -0.0077 | 0.0677 | 0.9093 | 11 | 61471678 | 40.29 |
| rs200949 | A | G | 0.048 | 0.0053 | 2.53E-19 | A | G | -0.0733 | 0.0884 | 0.4074 | 6 | 27835435 | 82.02 |
| rs2043539 | A | G | 0.0273 | 0.0035 | 9.89E-15 | A | G | 0.0288 | 0.0416 | 0.4891 | 7 | 12253880 | 60.84 |
| rs2187490 | T | G | -0.0338 | 0.0061 | 3.82E-08 | T | G | 0.078 | 0.0734 | 0.2884 | 11 | 118713180 | 30.70 |
| rs2509805 | T | C | 0.022 | 0.0038 | 9.17E-09 | T | C | 0.0473 | 0.0416 | 0.2557 | 11 | 57650796 | 33.52 |
| rs2568958 | A | G | 0.0373 | 0.0036 | 8.47E-25 | A | G | 0.0141 | 0.0421 | 0.7387 | 1 | 72765116 | 107.35 |
| rs2670139 | T | C | -0.0266 | 0.0041 | 1.21E-10 | T | C | 0.0605 | 0.0415 | 0.1454 | 9 | 126634255 | 42.09 |
| rs301799 | T | C | -0.025 | 0.0035 | 1.36E-12 | T | C | -0.0729 | 0.0423 | 0.08461 | 1 | 8489302 | 51.02 |
| rs30266 | A | G | 0.0308 | 0.0037 | 1.45E-16 | A | G | -0.0623 | 0.0444 | 0.1605 | 5 | 103972357 | 69.29 |
| rs3099439 | T | C | -0.0276 | 0.0035 | 5.05E-15 | T | C | 0.0199 | 0.0404 | 0.6226 | 5 | 87545318 | 62.18 |
| rs3213572 | A | G | 0.0217 | 0.0035 | 7.61E-10 | A | G | -0.0289 | 0.0405 | 0.4754 | 12 | 121205078 | 38.44 |
| rs33431 | T | C | 0.0198 | 0.0036 | 4.81E-08 | T | C | 0.0154 | 0.0421 | 0.715201 | 19 | 30939989 | 30.25 |
| rs34488670 | T | C | -0.0252 | 0.0043 | 6.03E-09 | T | C | -0.0043 | 0.048 | 0.928 | 15 | 47684936 | 34.35 |
| rs34937911 | T | C | 0.0304 | 0.0055 | 4.13E-08 | T | C | 0.0691 | 0.0639 | 0.2797 | 4 | 42110353 | 30.55 |
| rs35553410 | T | C | -0.0244 | 0.004 | 1.42E-09 | T | C | -0.0046 | 0.0466 | 0.9213 | 4 | 131239286 | 37.21 |
| rs3793577 | A | G | -0.0229 | 0.0035 | 8.41E-11 | A | G | -0.0331 | 0.0405 | 0.4133 | 9 | 23737627 | 42.81 |
| rs3823624 | T | C | 0.0272 | 0.0045 | 1.99E-09 | T | C | -0.0296 | 0.0449 | 0.508899 | 7 | 2110346 | 36.54 |
| rs4346585 | T | C | -0.0236 | 0.0038 | 7.13E-10 | T | C | -0.0508 | 0.0424 | 0.231 | 3 | 44736493 | 38.57 |
| rs45510091 | A | G | 0.0448 | 0.008 | 1.83E-08 | A | G | -0.0091 | 0.0934 | 0.9227 | 4 | 123186393 | 31.36 |
| rs4772087 | T | C | 0.0227 | 0.0036 | 3.91E-10 | T | C | -0.0097 | 0.0409 | 0.8127 | 13 | 99115041 | 39.76 |
| rs56314503 | T | G | -0.0254 | 0.004 | 2.95E-10 | T | G | -0.0643 | 0.0484 | 0.184 | 12 | 84465022 | 40.32 |
| rs56887639 | A | G | -0.0278 | 0.0039 | 1.51E-12 | A | G | 0.0094 | 0.0431 | 0.8277 | 16 | 13755530 | 50.81 |
| rs57344483 | A | G | -0.038 | 0.0068 | 1.82E-08 | A | G | -0.0814 | 0.0599 | 0.1745 | 11 | 127022560 | 31.23 |
| rs58104186 | A | G | 0.0237 | 0.0035 | 1.82E-11 | A | G | -0.0285 | 0.0404 | 0.4795 | 7 | 109099919 | 45.85 |
| rs59283172 | A | G | -0.0329 | 0.0057 | 1.02E-08 | A | G | 0.0379 | 0.0591 | 0.520701 | 9 | 25234672 | 33.32 |
| rs5995992 | T | C | -0.0266 | 0.0039 | 1.3E-11 | T | C | 0.054 | 0.0433 | 0.2124 | 22 | 41487218 | 46.52 |
| rs60157091 | T | C | 0.02 | 0.0035 | 1.42E-08 | T | C | 0.038 | 0.0402 | 0.345 | 5 | 61509655 | 32.65 |
| rs61902811 | A | G | -0.0257 | 0.0036 | 1.4E-12 | A | G | -0.0724 | 0.0499 | 0.1466 | 11 | 113370758 | 50.96 |
| rs61990288 | A | G | -0.026 | 0.0035 | 1.68E-13 | A | G | 0.0173 | 0.0402 | 0.666699 | 14 | 42074726 | 55.18 |
| rs62091461 | T | C | -0.0254 | 0.0042 | 1.95E-09 | T | C | 0.0068 | 0.0427 | 0.8738 | 18 | 52488672 | 36.57 |
| rs62188629 | A | G | 0.0236 | 0.0038 | 7.13E-10 | A | G | -0.0256 | 0.0418 | 0.54 | 2 | 208059440 | 38.57 |
| rs6783233 | T | C | 0.0218 | 0.0039 | 2.9E-8 | T | C | -0.0428 | 0.0491 | 0.3829 | 3 | 117509984 | 31.25 |
| rs7030813 | T | C | 0.0253 | 0.0036 | 3.07E-12 | T | C | -0.0121 | 0.0403 | 0.7648 | 9 | 36999369 | 49.39 |
| rs7117514 | A | G | -0.0204 | 0.0035 | 7.29E-09 | A | G | -0.0088 | 0.0405 | 0.8282 | 11 | 70544937 | 33.97 |
| rs7193263 | A | G | -0.0239 | 0.0038 | 4.33E-10 | A | G | -0.0082 | 0.0418 | 0.8452 | 16 | 6315880 | 39.56 |
| rs7198928 | T | C | 0.0239 | 0.0036 | 4.45E-11 | T | C | 0.0425 | 0.043 | 0.3237 | 16 | 7666402 | 44.07 |
| rs7200826 | T | C | 0.028 | 0.004 | 3.74E-12 | T | C | 0.0093 | 0.0425 | 0.8272 | 16 | 13066833 | 49.00 |
| rs7227069 | A | G | 0.0238 | 0.0035 | 1.5E-11 | A | G | -0.022 | 0.0408 | 0.589899 | 18 | 50731802 | 46.24 |
| rs7241572 | A | G | 0.028 | 0.0044 | 2.7E-10 | A | G | 0.0446 | 0.0489 | 0.3615 | 18 | 77580712 | 40.50 |
| rs725616 | T | C | 0.0204 | 0.0036 | 1.87E-08 | T | C | -0.0303 | 0.0411 | 0.4608 | 6 | 147950422 | 32.11 |
| rs72710803 | A | C | -0.041 | 0.0062 | 5.29E-11 | A | C | 0.0613 | 0.12 | 0.6096 | 1 | 177428018 | 43.73 |
| rs75581564 | A | G | 0.0301 | 0.0054 | 3.17E-08 | A | G | -0.1008 | 0.0591 | 0.08831 | 17 | 27363750 | 31.07 |
| rs7585722 | T | C | -0.0269 | 0.0048 | 2.68E-08 | T | C | -0.0834 | 0.0705 | 0.2363 | 2 | 86819128 | 31.41 |
| rs7624336 | T | G | 0.0238 | 0.0043 | 3.96E-08 | T | G | -0.0037 | 0.0544 | 0.9465 | 3 | 53244151 | 30.63 |
| rs7659414 | A | C | -0.0201 | 0.0035 | 1.2E-8 | A | C | 0.004 | 0.0418 | 0.9235 | 4 | 177350956 | 32.98 |
| rs7685686 | A | G | 0.0202 | 0.0036 | 2.57E-08 | A | G | 0.0669 | 0.0404 | 0.098141 | 4 | 3207142 | 31.48 |
| rs7807677 | T | C | 0.0237 | 0.0035 | 1.82E-11 | T | C | 0.0028 | 0.0416 | 0.9472 | 7 | 117502574 | 45.85 |
| rs78337797 | T | G | 0.0306 | 0.0055 | 3.37E-08 | T | G | 0.0668 | 0.0683 | 0.3276 | 12 | 23987925 | 30.95 |
| rs7837935 | T | G | -0.0292 | 0.0049 | 3.34E-09 | T | G | -0.052 | 0.0626 | 0.4065 | 8 | 65562019 | 35.51 |
| rs7932640 | T | C | 0.0281 | 0.0035 | 1.62E-15 | T | C | 0.0399 | 0.0403 | 0.3223 | 11 | 88744425 | 64.46 |
| rs8037355 | T | C | -0.0233 | 0.0035 | 3.94E-11 | T | C | 0.0187 | 0.0403 | 0.643101 | 15 | 37643831 | 44.32 |
| rs913930 | A | G | -0.0208 | 0.0037 | 2.42E-08 | A | G | 0.0782 | 0.0414 | 0.05879 | 9 | 120484009 | 31.60 |
| rs9363467 | T | C | 0.0237 | 0.0036 | 6.44E-11 | T | C | 0.0713 | 0.0416 | 0.086561 | 6 | 66605257 | 43.34 |
| rs9545360 | A | C | -0.0271 | 0.0046 | 5.02E-09 | A | C | -0.0236 | 0.0571 | 0.6798 | 13 | 80870712 | 34.71 |
| rs9592461 | A | G | 0.0216 | 0.0035 | 9.1E-10 | A | G | -0.0091 | 0.0404 | 0.8212 | 13 | 66941792 | 38.09 |
| rs997934 | T | C | 0.0198 | 0.0036 | 4.81E-08 | T | C | -0.0155 | 0.0404 | 0.7021 | 10 | 1795194 | 30.25 |
|  |  |  |  |  |  |  |  |  |  |  |  |  |  |
| SNP | Effects on depression | | | | | Effects on Pyogenic arthritis | | | | | Chr | Position | F |
|  | EA | OA | Beta | SE | p-val | EA | OA | Beta | SE | p-val |  |  | statistic |
| rs1002656 | T | C | -0.0266 | 0.0038 | 3.74E-12 | T | C | 0.0599 | 0.0467 | 0.1996 | 1 | 37192741 | 49.00 |
| rs10149470 | A | G | -0.0267 | 0.0035 | 3.72E-14 | A | G | -0.0081 | 0.0436 | 0.8527 | 14 | 104017953 | 58.20 |
| rs1021363 | A | G | 0.0303 | 0.0037 | 4.41E-16 | A | G | -0.0341 | 0.0485 | 0.4821 | 10 | 106610839 | 67.06 |
| rs1045430 | T | G | -0.0253 | 0.0035 | 7.31E-13 | T | G | 0.027 | 0.0433 | 0.5319 | 14 | 75130235 | 52.25 |
| rs10774600 | T | C | -0.0267 | 0.0048 | 3.39E-08 | T | C | -0.0011 | 0.0664 | 0.9867 | 12 | 110741356 | 30.94 |
| rs10789214 | T | C | 0.0193 | 0.0035 | 4.44E-08 | T | C | 0.0027 | 0.0437 | 0.9512 | 1 | 67146817 | 30.41 |
| rs10817969 | T | G | 0.0261 | 0.0039 | 3.11E-11 | T | G | -0.008 | 0.0464 | 0.8627 | 9 | 119731045 | 44.79 |
| rs10890020 | A | G | -0.0277 | 0.0035 | 4.03E-15 | A | G | -0.0252 | 0.0434 | 0.561 | 1 | 73669845 | 62.64 |
| rs10913112 | T | C | -0.0264 | 0.0036 | 3.4E-13 | T | C | -0.027 | 0.0448 | 0.5472 | 1 | 175913828 | 53.78 |
| rs1095626 | T | C | -0.0264 | 0.0035 | 7.13E-14 | T | C | 0.0017 | 0.0434 | 0.9693 | 3 | 157977962 | 56.89 |
| rs11135349 | A | C | -0.0295 | 0.0035 | 6.04E-17 | A | C | -0.0045 | 0.0445 | 0.9188 | 5 | 164523472 | 71.04 |
| rs113188507 | A | G | 0.0221 | 0.0039 | 1.87E-08 | A | G | -0.0414 | 0.0543 | 0.4456 | 1 | 80809636 | 32.11 |
| rs1152578 | T | C | -0.0218 | 0.0035 | 6.36E-10 | T | C | 0.0034 | 0.0436 | 0.9383 | 14 | 64697037 | 38.80 |
| rs11579246 | A | G | 0.0381 | 0.0061 | 5.71E-10 | A | G | 0.0209 | 0.0601 | 0.728299 | 1 | 50559162 | 39.01 |
| rs1226412 | T | C | 0.0256 | 0.0043 | 3.46E-09 | T | C | -0.0187 | 0.0504 | 0.710101 | 2 | 157111313 | 35.44 |
| rs12624433 | A | G | 0.0233 | 0.004 | 7.44E-09 | A | G | 0.0064 | 0.0491 | 0.8958 | 20 | 44680853 | 33.93 |
| rs12923444 | A | C | -0.0214 | 0.0035 | 1.3E-09 | A | C | -0.0578 | 0.0446 | 0.1948 | 16 | 21639710 | 37.38 |
| rs12967855 | A | G | 0.0265 | 0.0037 | 1.18E-12 | A | G | 0.0041 | 0.049 | 0.9326 | 18 | 35138245 | 51.30 |
| rs13084037 | A | G | -0.0245 | 0.0042 | 7.08E-09 | A | G | -0.0467 | 0.059 | 0.4294 | 3 | 49214066 | 34.03 |
| rs1343605 | A | C | 0.0313 | 0.0036 | 6.23E-18 | A | C | -0.058 | 0.0448 | 0.1952 | 13 | 53647048 | 75.59 |
| rs1354115 | A | C | 0.021 | 0.0036 | 7.08E-09 | A | C | 0.0416 | 0.0456 | 0.3621 | 9 | 2983774 | 34.03 |
| rs1409379 | T | C | 0.0249 | 0.0041 | 1.67E-09 | T | C | 0.0038 | 0.0468 | 0.9349 | 13 | 31907741 | 36.88 |
| rs141954845 | A | G | 0.0229 | 0.0037 | 8.15E-10 | A | G | 0.0652 | 0.0438 | 0.1363 | 3 | 61193647 | 38.31 |
| rs143186028 | T | G | 0.0277 | 0.0046 | 2.29E-09 | T | G | 0.0467 | 0.0499 | 0.35 | 20 | 39997404 | 36.26 |
| rs1448938 | A | G | 0.0214 | 0.0035 | 1.3E-09 | A | G | 0.0033 | 0.0431 | 0.9385 | 11 | 30892824 | 37.38 |
| rs1466887 | T | C | -0.0199 | 0.0036 | 4.12E-08 | T | C | 0.0029 | 0.0439 | 0.9473 | 1 | 37709328 | 30.56 |
| rs1568452 | T | C | 0.0248 | 0.0036 | 8.12E-12 | T | C | 0.0712 | 0.0456 | 0.1187 | 2 | 58012833 | 47.46 |
| rs16887442 | T | C | 0.0203 | 0.0035 | 8.62E-09 | T | C | 0.0132 | 0.0435 | 0.7619 | 7 | 82936909 | 33.64 |
| rs169235 | A | G | -0.0229 | 0.0041 | 2.98E-08 | A | G | 0.009 | 0.0504 | 0.859 | 1 | 181740924 | 31.20 |
| rs17641524 | T | C | -0.032 | 0.0043 | 1.52E-13 | T | C | 0.0385 | 0.061 | 0.5284 | 1 | 197704717 | 55.38 |
| rs1890946 | T | C | -0.0235 | 0.0035 | 2.68E-11 | T | C | -0.0206 | 0.0433 | 0.634501 | 1 | 52342427 | 45.08 |
| rs1956373 | T | G | -0.0226 | 0.004 | 2.06E-08 | T | G | 0.076 | 0.0555 | 0.1708 | 14 | 60141822 | 31.92 |
| rs1982277 | T | C | 0.0279 | 0.0041 | 1.45E-11 | T | C | -0.0622 | 0.0551 | 0.259 | 9 | 11513019 | 46.31 |
| rs198457 | T | C | -0.0292 | 0.0046 | 2.99E-10 | T | C | -0.0375 | 0.0726 | 0.6056 | 11 | 61471678 | 40.29 |
| rs200949 | A | G | 0.048 | 0.0053 | 2.53E-19 | A | G | 0.1699 | 0.0919 | 0.064441 | 6 | 27835435 | 82.02 |
| rs2043539 | A | G | 0.0273 | 0.0035 | 9.89E-15 | A | G | -0.0123 | 0.0448 | 0.783401 | 7 | 12253880 | 60.84 |
| rs2187490 | T | G | -0.0338 | 0.0061 | 3.82E-08 | T | G | -0.0323 | 0.0792 | 0.6831 | 11 | 118713180 | 30.70 |
| rs2509805 | T | C | 0.022 | 0.0038 | 9.17E-09 | T | C | -0.0574 | 0.0449 | 0.201 | 11 | 57650796 | 33.52 |
| rs2568958 | A | G | 0.0373 | 0.0036 | 8.47E-25 | A | G | 0.0452 | 0.0452 | 0.3177 | 1 | 72765116 | 107.35 |
| rs2670139 | T | C | -0.0266 | 0.0041 | 1.21E-10 | T | C | -0.0484 | 0.0448 | 0.2805 | 9 | 126634255 | 42.09 |
| rs301799 | T | C | -0.025 | 0.0035 | 1.36E-12 | T | C | 0.0225 | 0.0455 | 0.6216 | 1 | 8489302 | 51.02 |
| rs30266 | A | G | 0.0308 | 0.0037 | 1.45E-16 | A | G | 0.0556 | 0.0478 | 0.2441 | 5 | 103972357 | 69.29 |
| rs3099439 | T | C | -0.0276 | 0.0035 | 5.05E-15 | T | C | 0.0867 | 0.0435 | 0.04623 | 5 | 87545318 | 62.18 |
| rs3213572 | A | G | 0.0217 | 0.0035 | 7.61E-10 | A | G | 0.0267 | 0.0436 | 0.539699 | 12 | 121205078 | 38.44 |
| rs33431 | T | C | 0.0198 | 0.0036 | 4.81E-08 | T | C | -0.026 | 0.0451 | 0.564901 | 19 | 30939989 | 30.25 |
| rs34488670 | T | C | -0.0252 | 0.0043 | 6.03E-09 | T | C | 0.0438 | 0.052 | 0.4 | 15 | 47684936 | 34.35 |
| rs34937911 | T | C | 0.0304 | 0.0055 | 4.13E-08 | T | C | -0.0212 | 0.0685 | 0.7565 | 4 | 42110353 | 30.55 |
| rs35553410 | T | C | -0.0244 | 0.004 | 1.42E-09 | T | C | 0.0126 | 0.0504 | 0.803 | 4 | 131239286 | 37.21 |
| rs3793577 | A | G | -0.0229 | 0.0035 | 8.41E-11 | A | G | -0.0175 | 0.0435 | 0.6883 | 9 | 23737627 | 42.81 |
| rs3823624 | T | C | 0.0272 | 0.0045 | 1.99E-09 | T | C | 0.0223 | 0.0484 | 0.645601 | 7 | 2110346 | 36.54 |
| rs4346585 | T | C | -0.0236 | 0.0038 | 7.13E-10 | T | C | -0.0264 | 0.0459 | 0.5651 | 3 | 44736493 | 38.57 |
| rs45510091 | A | G | 0.0448 | 0.008 | 1.83E-08 | A | G | -0.0684 | 0.0994 | 0.4912 | 4 | 123186393 | 31.36 |
| rs4772087 | T | C | 0.0227 | 0.0036 | 3.91E-10 | T | C | -0.0102 | 0.0442 | 0.818 | 13 | 99115041 | 39.76 |
| rs56314503 | T | G | -0.0254 | 0.004 | 2.95E-10 | T | G | -0.0747 | 0.0523 | 0.1536 | 12 | 84465022 | 40.32 |
| rs56887639 | A | G | -0.0278 | 0.0039 | 1.51E-12 | A | G | 0.0383 | 0.0464 | 0.4094 | 16 | 13755530 | 50.81 |
| rs57344483 | A | G | -0.038 | 0.0068 | 1.82E-08 | A | G | 0.0188 | 0.0653 | 0.773599 | 11 | 127022560 | 31.23 |
| rs58104186 | A | G | 0.0237 | 0.0035 | 1.82E-11 | A | G | -0.0423 | 0.0436 | 0.3315 | 7 | 109099919 | 45.85 |
| rs59283172 | A | G | -0.0329 | 0.0057 | 1.02E-08 | A | G | 0.0326 | 0.0635 | 0.6076 | 9 | 25234672 | 33.32 |
| rs5995992 | T | C | -0.0266 | 0.0039 | 1.3E-11 | T | C | 0.0935 | 0.0466 | 0.04471 | 22 | 41487218 | 46.52 |
| rs60157091 | T | C | 0.02 | 0.0035 | 1.42E-08 | T | C | 0.0434 | 0.0434 | 0.317 | 5 | 61509655 | 32.65 |
| rs61902811 | A | G | -0.0257 | 0.0036 | 1.4E-12 | A | G | 0.0199 | 0.0533 | 0.7085 | 11 | 113370758 | 50.96 |
| rs61990288 | A | G | -0.026 | 0.0035 | 1.68E-13 | A | G | -0.0069 | 0.0431 | 0.8731 | 14 | 42074726 | 55.18 |
| rs62091461 | T | C | -0.0254 | 0.0042 | 1.95E-09 | T | C | -0.0361 | 0.0461 | 0.4338 | 18 | 52488672 | 36.57 |
| rs62188629 | A | G | 0.0236 | 0.0038 | 7.13E-10 | A | G | 0.0228 | 0.0451 | 0.612901 | 2 | 208059440 | 38.57 |
| rs6783233 | T | C | 0.0218 | 0.0039 | 2.9E-8 | T | C | -0.0036 | 0.0529 | 0.9456 | 3 | 117509984 | 31.25 |
| rs7030813 | T | C | 0.0253 | 0.0036 | 3.07E-12 | T | C | 0.0527 | 0.0434 | 0.225 | 9 | 36999369 | 49.39 |
| rs7117514 | A | G | -0.0204 | 0.0035 | 7.29E-09 | A | G | 0.0302 | 0.0436 | 0.4881 | 11 | 70544937 | 33.97 |
| rs7193263 | A | G | -0.0239 | 0.0038 | 4.33E-10 | A | G | 0.0123 | 0.0451 | 0.784401 | 16 | 6315880 | 39.56 |
| rs7198928 | T | C | 0.0239 | 0.0036 | 4.45E-11 | T | C | -0.0364 | 0.0464 | 0.4321 | 16 | 7666402 | 44.07 |
| rs7200826 | T | C | 0.028 | 0.004 | 3.74E-12 | T | C | -0.0173 | 0.046 | 0.7062 | 16 | 13066833 | 49.00 |
| rs7227069 | A | G | 0.0238 | 0.0035 | 1.5E-11 | A | G | 0.0286 | 0.0439 | 0.5152 | 18 | 50731802 | 46.24 |
| rs7241572 | A | G | 0.028 | 0.0044 | 2.7E-10 | A | G | 0.0172 | 0.0528 | 0.744699 | 18 | 77580712 | 40.50 |
| rs725616 | T | C | 0.0204 | 0.0036 | 1.87E-08 | T | C | -0.0008 | 0.0443 | 0.9859 | 6 | 147950422 | 32.11 |
| rs72710803 | A | C | -0.041 | 0.0062 | 5.29E-11 | A | C | 0.0006 | 0.1243 | 0.9961 | 1 | 177428018 | 43.73 |
| rs75581564 | A | G | 0.0301 | 0.0054 | 3.17E-08 | A | G | 0.0601 | 0.0639 | 0.347 | 17 | 27363750 | 31.07 |
| rs7585722 | T | C | -0.0269 | 0.0048 | 2.68E-08 | T | C | 0.0168 | 0.0755 | 0.8235 | 2 | 86819128 | 31.41 |
| rs7624336 | T | G | 0.0238 | 0.0043 | 3.96E-08 | T | G | -0.0016 | 0.0583 | 0.9781 | 3 | 53244151 | 30.63 |
| rs7659414 | A | C | -0.0201 | 0.0035 | 1.2E-8 | A | C | -0.0072 | 0.0451 | 0.8724 | 4 | 177350956 | 32.98 |
| rs7685686 | A | G | 0.0202 | 0.0036 | 2.57E-08 | A | G | 0.0348 | 0.0435 | 0.4235 | 4 | 3207142 | 31.48 |
| rs7807677 | T | C | 0.0237 | 0.0035 | 1.82E-11 | T | C | 0.0658 | 0.0448 | 0.1425 | 7 | 117502574 | 45.85 |
| rs78337797 | T | G | 0.0306 | 0.0055 | 3.37E-08 | T | G | 0.0125 | 0.0727 | 0.8634 | 12 | 23987925 | 30.95 |
| rs7837935 | T | G | -0.0292 | 0.0049 | 3.34E-09 | T | G | 0.1327 | 0.0664 | 0.04567 | 8 | 65562019 | 35.51 |
| rs7932640 | T | C | 0.0281 | 0.0035 | 1.62E-15 | T | C | 0.0154 | 0.0435 | 0.7224 | 11 | 88744425 | 64.46 |
| rs8037355 | T | C | -0.0233 | 0.0035 | 3.94E-11 | T | C | -0.0466 | 0.0434 | 0.2825 | 15 | 37643831 | 44.32 |
| rs913930 | A | G | -0.0208 | 0.0037 | 2.42E-08 | A | G | 0.0158 | 0.0446 | 0.7229 | 9 | 120484009 | 31.60 |
| rs9363467 | T | C | 0.0237 | 0.0036 | 6.44E-11 | T | C | -0.119 | 0.0449 | 0.008024 | 6 | 66605257 | 43.34 |
| rs9545360 | A | C | -0.0271 | 0.0046 | 5.02E-09 | A | C | 0.0408 | 0.0612 | 0.5048 | 13 | 80870712 | 34.71 |
| rs9592461 | A | G | 0.0216 | 0.0035 | 9.1E-10 | A | G | 0.0641 | 0.0435 | 0.14 | 13 | 66941792 | 38.09 |
| rs997934 | T | C | 0.0198 | 0.0036 | 4.81E-08 | T | C | -0.0571 | 0.0435 | 0.1898 | 10 | 1795194 | 30.25 |
|  |  |  |  |  |  |  |  |  |  |  |  |  |  |
| SNP | Effects on depression | | | | | Effects on gout | | | | | Chr | Position | F |
|  | EA | OA | Beta | SE | p-val | EA | OA | Beta | SE | p-val |  |  | statistic |
| rs1002656 | T | C | -0.0266 | 0.0038 | 3.74E-12 | T | C | -0.0261 | 0.0273 | 0.3386 | 1 | 37192741 | 49.00 |
| rs10149470 | A | G | -0.0267 | 0.0035 | 3.72E-14 | A | G | -0.0375 | 0.0255 | 0.1409 | 14 | 104017953 | 58.20 |
| rs1021363 | A | G | 0.0303 | 0.0037 | 4.41E-16 | A | G | -0.0123 | 0.0283 | 0.6635 | 10 | 106610839 | 67.06 |
| rs1045430 | T | G | -0.0253 | 0.0035 | 7.31E-13 | T | G | -0.0099 | 0.0252 | 0.6951 | 14 | 75130235 | 52.25 |
| rs10774600 | T | C | -0.0267 | 0.0048 | 3.39E-08 | T | C | 0.0056 | 0.0388 | 0.8852 | 12 | 110741356 | 30.94 |
| rs10789214 | T | C | 0.0193 | 0.0035 | 4.44E-08 | T | C | 0.0455 | 0.0255 | 0.074269 | 1 | 67146817 | 30.41 |
| rs10817969 | T | G | 0.0261 | 0.0039 | 3.11E-11 | T | G | 0.0033 | 0.0271 | 0.9043 | 9 | 119731045 | 44.79 |
| rs10890020 | A | G | -0.0277 | 0.0035 | 4.03E-15 | A | G | -0.002 | 0.0253 | 0.9355 | 1 | 73669845 | 62.64 |
| rs10913112 | T | C | -0.0264 | 0.0036 | 3.4E-13 | T | C | -0.0334 | 0.0262 | 0.202 | 1 | 175913828 | 53.78 |
| rs1095626 | T | C | -0.0264 | 0.0035 | 7.13E-14 | T | C | 0.0301 | 0.0253 | 0.2339 | 3 | 157977962 | 56.89 |
| rs11135349 | A | C | -0.0295 | 0.0035 | 6.04E-17 | A | C | 0.0329 | 0.0259 | 0.2042 | 5 | 164523472 | 71.04 |
| rs113188507 | A | G | 0.0221 | 0.0039 | 1.87E-08 | A | G | 0.0175 | 0.0317 | 0.5818 | 1 | 80809636 | 32.11 |
| rs1152578 | T | C | -0.0218 | 0.0035 | 6.36E-10 | T | C | 0.0013 | 0.0255 | 0.9596 | 14 | 64697037 | 38.80 |
| rs11579246 | A | G | 0.0381 | 0.0061 | 5.71E-10 | A | G | -0.0187 | 0.035 | 0.593 | 1 | 50559162 | 39.01 |
| rs1226412 | T | C | 0.0256 | 0.0043 | 3.46E-09 | T | C | 0.0303 | 0.0293 | 0.3015 | 2 | 157111313 | 35.44 |
| rs12624433 | A | G | 0.0233 | 0.004 | 7.44E-09 | A | G | -0.0153 | 0.0286 | 0.5924 | 20 | 44680853 | 33.93 |
| rs12923444 | A | C | -0.0214 | 0.0035 | 1.3E-09 | A | C | -0.0465 | 0.0259 | 0.07325 | 16 | 21639710 | 37.38 |
| rs12967855 | A | G | 0.0265 | 0.0037 | 1.18E-12 | A | G | 0.0503 | 0.0284 | 0.076961 | 18 | 35138245 | 51.30 |
| rs13084037 | A | G | -0.0245 | 0.0042 | 7.08E-09 | A | G | 0.0088 | 0.0345 | 0.7979 | 3 | 49214066 | 34.03 |
| rs1343605 | A | C | 0.0313 | 0.0036 | 6.23E-18 | A | C | 0.0217 | 0.0261 | 0.4057 | 13 | 53647048 | 75.59 |
| rs1354115 | A | C | 0.021 | 0.0036 | 7.08E-09 | A | C | -0.0114 | 0.0266 | 0.6691 | 9 | 2983774 | 34.03 |
| rs1409379 | T | C | 0.0249 | 0.0041 | 1.67E-09 | T | C | -0.007 | 0.0273 | 0.7969 | 13 | 31907741 | 36.88 |
| rs141954845 | A | G | 0.0229 | 0.0037 | 8.15E-10 | A | G | 0.0156 | 0.0255 | 0.542199 | 3 | 61193647 | 38.31 |
| rs143186028 | T | G | 0.0277 | 0.0046 | 2.29E-09 | T | G | -0.0156 | 0.0291 | 0.5903 | 20 | 39997404 | 36.26 |
| rs1448938 | A | G | 0.0214 | 0.0035 | 1.3E-09 | A | G | 0.065 | 0.0251 | 0.009718 | 11 | 30892824 | 37.38 |
| rs1466887 | T | C | -0.0199 | 0.0036 | 4.12E-08 | T | C | -0.008 | 0.0257 | 0.7545 | 1 | 37709328 | 30.56 |
| rs1568452 | T | C | 0.0248 | 0.0036 | 8.12E-12 | T | C | -0.0039 | 0.0266 | 0.8822 | 2 | 58012833 | 47.46 |
| rs16887442 | T | C | 0.0203 | 0.0035 | 8.62E-09 | T | C | -0.0065 | 0.0253 | 0.7963 | 7 | 82936909 | 33.64 |
| rs169235 | A | G | -0.0229 | 0.0041 | 2.98E-08 | A | G | -0.0078 | 0.0294 | 0.791199 | 1 | 181740924 | 31.20 |
| rs17641524 | T | C | -0.032 | 0.0043 | 1.52E-13 | T | C | 0.004 | 0.0355 | 0.91 | 1 | 197704717 | 55.38 |
| rs1890946 | T | C | -0.0235 | 0.0035 | 2.68E-11 | T | C | -0.0493 | 0.0252 | 0.05022 | 1 | 52342427 | 45.08 |
| rs1956373 | T | G | -0.0226 | 0.004 | 2.06E-08 | T | G | 0.0196 | 0.0324 | 0.5456 | 14 | 60141822 | 31.92 |
| rs1982277 | T | C | 0.0279 | 0.0041 | 1.45E-11 | T | C | -0.0023 | 0.0321 | 0.9424 | 9 | 11513019 | 46.31 |
| rs198457 | T | C | -0.0292 | 0.0046 | 2.99E-10 | T | C | -0.0126 | 0.0423 | 0.7668 | 11 | 61471678 | 40.29 |
| rs200949 | A | G | 0.048 | 0.0053 | 2.53E-19 | A | G | -0.1171 | 0.0525 | 0.02584 | 6 | 27835435 | 82.02 |
| rs2043539 | A | G | 0.0273 | 0.0035 | 9.89E-15 | A | G | 0.0327 | 0.0261 | 0.2099 | 7 | 12253880 | 60.84 |
| rs2187490 | T | G | -0.0338 | 0.0061 | 3.82E-08 | T | G | -0.0711 | 0.0463 | 0.1243 | 11 | 118713180 | 30.70 |
| rs2509805 | T | C | 0.022 | 0.0038 | 9.17E-09 | T | C | 0.0291 | 0.0261 | 0.2648 | 11 | 57650796 | 33.52 |
| rs2568958 | A | G | 0.0373 | 0.0036 | 8.47E-25 | A | G | 0.002 | 0.0265 | 0.9386 | 1 | 72765116 | 107.35 |
| rs2670139 | T | C | -0.0266 | 0.0041 | 1.21E-10 | T | C | 0.0041 | 0.0262 | 0.8763 | 9 | 126634255 | 42.09 |
| rs301799 | T | C | -0.025 | 0.0035 | 1.36E-12 | T | C | 0.0092 | 0.0265 | 0.728201 | 1 | 8489302 | 51.02 |
| rs30266 | A | G | 0.0308 | 0.0037 | 1.45E-16 | A | G | 0.0171 | 0.0279 | 0.5401 | 5 | 103972357 | 69.29 |
| rs3099439 | T | C | -0.0276 | 0.0035 | 5.05E-15 | T | C | 0.0253 | 0.0254 | 0.3193 | 5 | 87545318 | 62.18 |
| rs3213572 | A | G | 0.0217 | 0.0035 | 7.61E-10 | A | G | 0.0557 | 0.0254 | 0.0282 | 12 | 121205078 | 38.44 |
| rs33431 | T | C | 0.0198 | 0.0036 | 4.81E-08 | T | C | 0.0448 | 0.0264 | 0.089801 | 19 | 30939989 | 30.25 |
| rs34488670 | T | C | -0.0252 | 0.0043 | 6.03E-09 | T | C | 0.0098 | 0.0303 | 0.7466 | 15 | 47684936 | 34.35 |
| rs34937911 | T | C | 0.0304 | 0.0055 | 4.13E-08 | T | C | 0.0342 | 0.0399 | 0.3915 | 4 | 42110353 | 30.55 |
| rs35553410 | T | C | -0.0244 | 0.004 | 1.42E-09 | T | C | 0.041 | 0.0293 | 0.1619 | 4 | 131239286 | 37.21 |
| rs3793577 | A | G | -0.0229 | 0.0035 | 8.41E-11 | A | G | -0.0329 | 0.0253 | 0.1943 | 9 | 23737627 | 42.81 |
| rs3823624 | T | C | 0.0272 | 0.0045 | 1.99E-09 | T | C | 0.0799 | 0.0284 | 0.004864 | 7 | 2110346 | 36.54 |
| rs4346585 | T | C | -0.0236 | 0.0038 | 7.13E-10 | T | C | 0.0068 | 0.0268 | 0.7984 | 3 | 44736493 | 38.57 |
| rs45510091 | A | G | 0.0448 | 0.008 | 1.83E-08 | A | G | 0.1098 | 0.0582 | 0.05928 | 4 | 123186393 | 31.36 |
| rs4772087 | T | C | 0.0227 | 0.0036 | 3.91E-10 | T | C | -0.044 | 0.0258 | 0.087289 | 13 | 99115041 | 39.76 |
| rs56314503 | T | G | -0.0254 | 0.004 | 2.95E-10 | T | G | 0.0291 | 0.0306 | 0.3412 | 12 | 84465022 | 40.32 |
| rs56887639 | A | G | -0.0278 | 0.0039 | 1.51E-12 | A | G | 0.0292 | 0.0271 | 0.2819 | 16 | 13755530 | 50.81 |
| rs57344483 | A | G | -0.038 | 0.0068 | 1.82E-08 | A | G | -0.0088 | 0.0381 | 0.8173 | 11 | 127022560 | 31.23 |
| rs58104186 | A | G | 0.0237 | 0.0035 | 1.82E-11 | A | G | 0.0204 | 0.0254 | 0.4229 | 7 | 109099919 | 45.85 |
| rs59283172 | A | G | -0.0329 | 0.0057 | 1.02E-08 | A | G | -0.0031 | 0.037 | 0.9333 | 9 | 25234672 | 33.32 |
| rs5995992 | T | C | -0.0266 | 0.0039 | 1.3E-11 | T | C | -0.0335 | 0.0271 | 0.2163 | 22 | 41487218 | 46.52 |
| rs60157091 | T | C | 0.02 | 0.0035 | 1.42E-08 | T | C | 0.0411 | 0.0253 | 0.1044 | 5 | 61509655 | 32.65 |
| rs61902811 | A | G | -0.0257 | 0.0036 | 1.4E-12 | A | G | -0.0517 | 0.031 | 0.09562 | 11 | 113370758 | 50.96 |
| rs61990288 | A | G | -0.026 | 0.0035 | 1.68E-13 | A | G | -0.0598 | 0.0251 | 0.01714 | 14 | 42074726 | 55.18 |
| rs62091461 | T | C | -0.0254 | 0.0042 | 1.95E-09 | T | C | 0.0054 | 0.0268 | 0.8414 | 18 | 52488672 | 36.57 |
| rs62188629 | A | G | 0.0236 | 0.0038 | 7.13E-10 | A | G | -0.0231 | 0.0263 | 0.3802 | 2 | 208059440 | 38.57 |
| rs6783233 | T | C | 0.0218 | 0.0039 | 2.9E-8 | T | C | 0.0223 | 0.0309 | 0.4711 | 3 | 117509984 | 31.25 |
| rs7030813 | T | C | 0.0253 | 0.0036 | 3.07E-12 | T | C | 0.049 | 0.0253 | 0.05246 | 9 | 36999369 | 49.39 |
| rs7117514 | A | G | -0.0204 | 0.0035 | 7.29E-09 | A | G | -0.0298 | 0.0254 | 0.2407 | 11 | 70544937 | 33.97 |
| rs7193263 | A | G | -0.0239 | 0.0038 | 4.33E-10 | A | G | 0.0244 | 0.0263 | 0.3545 | 16 | 6315880 | 39.56 |
| rs7198928 | T | C | 0.0239 | 0.0036 | 4.45E-11 | T | C | 0.0017 | 0.0271 | 0.949 | 16 | 7666402 | 44.07 |
| rs7200826 | T | C | 0.028 | 0.004 | 3.74E-12 | T | C | -0.0469 | 0.0268 | 0.08053 | 16 | 13066833 | 49.00 |
| rs7227069 | A | G | 0.0238 | 0.0035 | 1.5E-11 | A | G | -0.0092 | 0.0256 | 0.720299 | 18 | 50731802 | 46.24 |
| rs7241572 | A | G | 0.028 | 0.0044 | 2.7E-10 | A | G | -0.0017 | 0.0308 | 0.9558 | 18 | 77580712 | 40.50 |
| rs725616 | T | C | 0.0204 | 0.0036 | 1.87E-08 | T | C | -0.0204 | 0.0259 | 0.4305 | 6 | 147950422 | 32.11 |
| rs72710803 | A | C | -0.041 | 0.0062 | 5.29E-11 | A | C | 0.0757 | 0.0733 | 0.3019 | 1 | 177428018 | 43.73 |
| rs75581564 | A | G | 0.0301 | 0.0054 | 3.17E-08 | A | G | 0.0311 | 0.0372 | 0.4025 | 17 | 27363750 | 31.07 |
| rs7585722 | T | C | -0.0269 | 0.0048 | 2.68E-08 | T | C | -0.0142 | 0.044 | 0.746799 | 2 | 86819128 | 31.41 |
| rs7624336 | T | G | 0.0238 | 0.0043 | 3.96E-08 | T | G | -0.0536 | 0.0341 | 0.116 | 3 | 53244151 | 30.63 |
| rs7659414 | A | C | -0.0201 | 0.0035 | 1.2E-8 | A | C | 0.0029 | 0.0263 | 0.9129 | 4 | 177350956 | 32.98 |
| rs7685686 | A | G | 0.0202 | 0.0036 | 2.57E-08 | A | G | 0.0227 | 0.0253 | 0.3709 | 4 | 3207142 | 31.48 |
| rs7807677 | T | C | 0.0237 | 0.0035 | 1.82E-11 | T | C | -0.0138 | 0.0261 | 0.596901 | 7 | 117502574 | 45.85 |
| rs78337797 | T | G | 0.0306 | 0.0055 | 3.37E-08 | T | G | 0.0002 | 0.0424 | 0.9954 | 12 | 23987925 | 30.95 |
| rs7837935 | T | G | -0.0292 | 0.0049 | 3.34E-09 | T | G | -0.0383 | 0.0389 | 0.3255 | 8 | 65562019 | 35.51 |
| rs7932640 | T | C | 0.0281 | 0.0035 | 1.62E-15 | T | C | 0.0139 | 0.0253 | 0.584201 | 11 | 88744425 | 64.46 |
| rs8037355 | T | C | -0.0233 | 0.0035 | 3.94E-11 | T | C | 0.0076 | 0.0253 | 0.7642 | 15 | 37643831 | 44.32 |
| rs913930 | A | G | -0.0208 | 0.0037 | 2.42E-08 | A | G | 0.0308 | 0.0259 | 0.2353 | 9 | 120484009 | 31.60 |
| rs9363467 | T | C | 0.0237 | 0.0036 | 6.44E-11 | T | C | -0.0081 | 0.0261 | 0.7573 | 6 | 66605257 | 43.34 |
| rs9545360 | A | C | -0.0271 | 0.0046 | 5.02E-09 | A | C | -0.0026 | 0.0358 | 0.9415 | 13 | 80870712 | 34.71 |
| rs9592461 | A | G | 0.0216 | 0.0035 | 9.1E-10 | A | G | 0.011 | 0.0254 | 0.6654 | 13 | 66941792 | 38.09 |
| rs997934 | T | C | 0.0198 | 0.0036 | 4.81E-08 | T | C | 0.0018 | 0.0254 | 0.9423 | 10 | 1795194 | 30.25 |

OA：osteoarthritis;KOA：knee osteoarthritis;HOA：hip osteoarthritis;AS：ankylosing spondylitis;Seronegative RA：Seronegative rheumatoid arthritis;Seropositive RA：Seropositive rheumatoid arthritis;MR-PRESSO：MR pleiotropy residual sum and outlier;SNP：single-nucleotide polymorphism.
