# Supplementary material for: Depression and risk of arthritis: A Mendelian randomization study
Source: Brain Behav. 2024 Jun 7;14(6):e3551. doi: 10.1002/brb3.3551 (PMC11161388; doi:10.1002/brb3.3551)
Supplement: Supplementary file 21 — Supporting Information [file BRB3-14-e3551-s003.docx]

| **Table S3.** Characteristics of the SNPs related to depression and different types of arthritis | | | | | | | | | | | | | |
| --- | --- | --- | --- | --- | --- | --- | --- | --- | --- | --- | --- | --- | --- |
|  |  |  |  |  |  |  |  |  |  |  |  |  |  |
| SNP | Effects on OA | | | | | Effects on depression | | | | | Chr | Position | F |
|  | EA | OA | Beta | SE | p-val | EA | OA | Beta | SE | p-val |  |  | statistic |
| rs10112083 | G | C | 0.003423 | 0.000722 | 0.00000213 | G | C | 0.000413 | 0.000517 | 0.43 | 8 | 3660204 | 22.48 |
| rs10929072 | C | T | -0.0037 | 0.000685 | 6.74E-08 | C | T | 0.000011 | 0.000491 | 0.98 | 2 | 234086900 | 29.18 |
| rs112715306 | A | G | 0.006515 | 0.001412 | 0.00000395 | A | G | 0.001607 | 0.001007 | 0.11 | 7 | 124360975 | 21.29 |
| rs113471597 | G | C | 0.003827 | 0.00079 | 0.00000128 | G | C | 0.000672 | 0.000567 | 0.24 | 7 | 6532167 | 23.47 |
| rs114680493 | C | T | 0.010551 | 0.00231 | 0.00000491 | C | T | 0.001548 | 0.001629 | 0.34 | 18 | 71818161 | 20.86 |
| rs11809524 | T | C | 0.004506 | 0.000918 | 0.000000919 | T | C | 0.00018 | 0.000657 | 0.780001 | 1 | 103459537 | 24.09 |
| rs12154055 | A | G | -0.00327 | 0.000691 | 0.0000022 | A | G | -0.00011 | 0.000496 | 0.83 | 6 | 44449697 | 22.39 |
| rs13107325 | T | C | 0.009966 | 0.001282 | 7.79E-15 | T | C | 0.001076 | 0.000918 | 0.24 | 4 | 103188709 | 60.43 |
| rs143582413 | T | G | 0.006712 | 0.001271 | 0.00000013 | T | G | 0.001179 | 0.000916 | 0.2 | 6 | 33443005 | 27.89 |
| rs147760547 | G | A | 0.010462 | 0.002164 | 0.00000134 | G | A | 0.001576 | 0.001569 | 0.32 | 15 | 78816130 | 23.37 |
| rs1560707 | G | T | -0.00349 | 0.000699 | 0.000000588 | G | T | -0.00018 | 0.000501 | 0.709999 | 19 | 10750738 | 24.93 |
| rs1642595 | A | G | -0.00533 | 0.001108 | 0.00000149 | A | G | 0.000899 | 0.000792 | 0.26 | 17 | 42065227 | 23.14 |
| rs1713484 | G | A | -0.00366 | 0.000782 | 0.00000286 | G | A | -0.00018 | 0.000561 | 0.75 | 16 | 12745851 | 21.91 |
| rs2047885 | C | G | -0.00356 | 0.000692 | 0.00000026 | C | G | -0.000067 | 0.000496 | 0.89 | 3 | 1117149 | 26.47 |
| rs2290573 | A | G | 0.003256 | 0.000686 | 0.00000209 | A | G | 0.000637 | 0.000491 | 0.19 | 15 | 75129594 | 22.53 |
| rs2807867 | A | C | -0.00333 | 0.000675 | 0.000000845 | A | C | -0.00079 | 0.000484 | 0.1 | 1 | 221082794 | 24.34 |
| rs2835328 | C | A | 0.003596 | 0.000727 | 0.000000755 | C | A | -0.00011 | 0.000522 | 0.83 | 21 | 37622915 | 24.47 |
| rs2997925 | G | A | -0.0034 | 0.000721 | 0.00000236 | G | A | 0.000154 | 0.000518 | 0.77 | 10 | 51832748 | 22.24 |
| rs3771501 | G | A | -0.00442 | 0.000677 | 6.61E-11 | G | A | -0.00045 | 0.000485 | 0.35 | 2 | 70717653 | 42.63 |
| rs42079 | G | A | -0.0032 | 0.000676 | 0.00000218 | G | A | -0.00151 | 0.000485 | 0.0018 | 7 | 95711301 | 22.41 |
| rs4978572 | G | C | 0.003476 | 0.000687 | 0.000000418 | G | C | 0.000352 | 0.000492 | 0.47 | 9 | 116943302 | 25.60 |
| rs61734410 | T | C | -0.00396 | 0.000793 | 0.000000618 | T | C | -0.00117 | 0.000567 | 0.04 | 16 | 1252369 | 24.94 |
| rs62124868 | C | T | 0.004546 | 0.000863 | 0.00000014 | C | T | 0.000557 | 0.000617 | 0.37 | 2 | 22620162 | 27.75 |
| rs62355921 | G | A | 0.004634 | 0.000948 | 0.00000103 | G | A | -0.00014 | 0.000678 | 0.84 | 5 | 107108006 | 23.89 |
| rs6701965 | T | C | -0.0042 | 0.000882 | 0.00000197 | T | C | -0.00018 | 0.000632 | 0.780001 | 1 | 11681568 | 22.68 |
| rs6724084 | A | G | -0.00346 | 0.000712 | 0.00000121 | A | G | 0.000062 | 0.00051 | 0.9 | 2 | 142476207 | 23.62 |
| rs6863101 | A | G | -0.00432 | 0.000898 | 0.0000015 | A | G | -0.00103 | 0.000644 | 0.11 | 5 | 170761347 | 23.14 |
| rs72805578 | A | C | -0.00433 | 0.000827 | 0.000000164 | A | C | 0.000609 | 0.000595 | 0.31 | 16 | 89682491 | 27.41 |
| rs75621460 | A | G | 0.011431 | 0.002274 | 0.000000501 | A | G | 0.000357 | 0.001636 | 0.83 | 19 | 41833784 | 25.27 |
| rs77817631 | A | G | -0.01258 | 0.002712 | 0.00000347 | A | G | -0.00384 | 0.001958 | 0.05 | 3 | 73477270 | 21.52 |
| rs7915299 | G | T | 0.012648 | 0.002592 | 0.00000106 | G | T | -0.00372 | 0.001881 | 0.048 | 10 | 6409592 | 23.81 |
| rs8102677 | G | A | -0.00347 | 0.000725 | 0.00000175 | G | A | -0.00094 | 0.00052 | 0.071 | 19 | 4018100 | 22.91 |
| rs881009 | T | C | -0.00317 | 0.000693 | 0.00000461 | T | C | -0.00021 | 0.000495 | 0.68 | 10 | 80296708 | 20.92 |
|  |  |  |  |  |  |  |  |  |  |  |  |  |  |
| SNP | Effects on KOA | | | | | Effects on depression | | | | | Chr | Position | F |
|  | EA | OA | Beta | SE | p-val | EA | OA | Beta | SE | p-val |  |  | statistic |
| rs10042443 | T | C | -0.0494 | 0.0099 | 0.00000058 | T | C | -0.00081 | 0.000515 | 0.11 | 5 | 55352770 | 24.90 |
| rs10053979 | G | A | 0.0571 | 0.0108 | 0.000000138 | G | A | 0.000191 | 0.000565 | 0.74 | 5 | 141774373 | 27.95 |
| rs10181210 | G | A | 0.0838 | 0.0158 | 0.000000113 | G | A | 0.00065 | 0.00082 | 0.43 | 2 | 16901980 | 28.13 |
| rs10228472 | C | T | 0.0503 | 0.0098 | 0.000000284 | C | T | 0.001429 | 0.000511 | 0.0052 | 7 | 95711278 | 26.34 |
| rs1029425 | T | G | -0.0484 | 0.0098 | 0.000000774 | T | G | -0.00054 | 0.000511 | 0.29 | 9 | 81294685 | 24.39 |
| rs1078301 | T | A | 0.0679 | 0.0106 | 1.27E-10 | T | A | 0.000198 | 0.000551 | 0.719999 | 9 | 116909146 | 41.03 |
| rs111701331 | T | C | -0.1078 | 0.0233 | 0.00000361 | T | C | 0.001282 | 0.001211 | 0.29 | 3 | 150647801 | 21.41 |
| rs112534929 | A | G | -0.0857 | 0.0178 | 0.0000015 | A | G | 0.000653 | 0.000928 | 0.48 | 18 | 45777464 | 23.18 |
| rs112957563 | T | C | 0.0576 | 0.0117 | 0.000000885 | T | C | 0.000925 | 0.000612 | 0.13 | 7 | 6531355 | 24.24 |
| rs114803656 | T | C | 0.1026 | 0.0218 | 0.00000259 | T | C | 0.000701 | 0.001141 | 0.54 | 1 | 70053590 | 22.15 |
| rs11612970 | C | T | 0.0688 | 0.0135 | 0.000000333 | C | T | 0.000326 | 0.000663 | 0.62 | 12 | 48456823 | 25.97 |
| rs11705555 | C | A | -0.0498 | 0.0107 | 0.00000346 | C | A | -0.00059 | 0.000558 | 0.29 | 22 | 28206912 | 21.66 |
| rs117830633 | A | G | 0.1028 | 0.0222 | 0.00000365 | A | G | -0.00197 | 0.001154 | 0.088 | 16 | 48411484 | 21.44 |
| rs12205483 | T | C | 0.1456 | 0.0294 | 0.000000711 | T | C | 0.002925 | 0.001535 | 0.056999 | 6 | 21309632 | 24.53 |
| rs12314838 | C | T | 0.0484 | 0.01 | 0.00000121 | C | T | 0.000643 | 0.00052 | 0.22 | 12 | 94195307 | 23.43 |
| rs12421186 | T | C | 0.0472 | 0.0102 | 0.00000335 | T | C | -0.00034 | 0.00053 | 0.52 | 11 | 120464049 | 21.41 |
| rs12664526 | C | T | -0.0547 | 0.0101 | 5.93E-08 | C | T | 0.000732 | 0.000526 | 0.16 | 6 | 102763549 | 29.33 |
| rs12913453 | G | A | 0.0552 | 0.0106 | 0.000000199 | G | A | 0.000634 | 0.000554 | 0.25 | 15 | 62015217 | 27.12 |
| rs12986150 | T | C | -0.0703 | 0.0135 | 0.000000179 | T | C | -0.0005 | 0.0007 | 0.47 | 19 | 18073659 | 27.12 |
| rs13018037 | A | G | -0.0616 | 0.0134 | 0.00000392 | A | G | -0.00075 | 0.000697 | 0.28 | 2 | 199117963 | 21.13 |
| rs13086966 | C | G | -0.0541 | 0.01 | 6.02E-08 | C | G | -0.00142 | 0.00052 | 0.0062 | 3 | 131782871 | 29.27 |
| rs13394110 | G | A | 0.0584 | 0.012 | 0.00000113 | G | A | 0.001189 | 0.000625 | 0.056999 | 2 | 100873975 | 23.68 |
| rs1409263 | T | C | 0.1118 | 0.0242 | 0.00000391 | T | C | 0.001614 | 0.001272 | 0.2 | 13 | 93425642 | 21.34 |
| rs142320277 | C | G | 0.1603 | 0.035 | 0.00000466 | C | G | 0.003874 | 0.001852 | 0.037 | 2 | 141422979 | 20.98 |
| rs143384 | G | A | -0.0935 | 0.0095 | 4.77E-23 | G | A | -0.00092 | 0.000492 | 0.061 | 20 | 34025756 | 96.87 |
| rs143870933 | A | G | 0.2294 | 0.0491 | 0.00000302 | A | G | -0.00061 | 0.002428 | 0.8 | 16 | 820603 | 21.83 |
| rs148995486 | C | G | 0.169 | 0.0352 | 0.00000153 | C | G | -0.00269 | 0.00185 | 0.15 | 5 | 122947707 | 23.05 |
| rs151431 | A | G | 0.0533 | 0.0116 | 0.00000442 | A | G | -0.000041 | 0.000571 | 0.94 | 4 | 103132210 | 21.11 |
| rs17659798 | C | A | -0.054 | 0.0104 | 0.000000182 | C | A | 0.000335 | 0.000539 | 0.53 | 11 | 28874997 | 26.96 |
| rs1800472 | A | G | 0.1539 | 0.0327 | 0.00000263 | A | G | -0.0002 | 0.001654 | 0.91 | 19 | 41847860 | 22.15 |
| rs1952689 | C | G | -0.0469 | 0.0097 | 0.00000141 | C | G | 0.0000915 | 0.000505 | 0.86 | 9 | 16723226 | 23.38 |
| rs1982418 | A | G | 0.048 | 0.01 | 0.00000147 | A | G | 0.000695 | 0.000519 | 0.18 | 5 | 101638381 | 23.04 |
| rs2160930 | G | A | -0.0443 | 0.0093 | 0.00000199 | G | A | -0.00046 | 0.000486 | 0.35 | 2 | 53882527 | 22.69 |
| rs2294098 | A | G | 0.1354 | 0.0249 | 5.23E-08 | A | G | -0.00121 | 0.001293 | 0.35 | 8 | 42148049 | 29.57 |
| rs2472297 | T | C | 0.0557 | 0.0105 | 0.000000111 | T | C | 0.001001 | 0.000548 | 0.068 | 15 | 75027880 | 28.14 |
| rs2741389 | C | T | 0.0465 | 0.0097 | 0.00000176 | C | T | -0.00012 | 0.000507 | 0.81 | 20 | 52100468 | 22.98 |
| rs2820430 | C | T | -0.0494 | 0.0101 | 0.000000881 | C | T | -0.00049 | 0.000524 | 0.35 | 1 | 219651779 | 23.92 |
| rs2908729 | G | A | -0.0506 | 0.01 | 0.000000401 | G | A | 0.0000978 | 0.000521 | 0.85 | 7 | 12075642 | 25.60 |
| rs3122169 | A | C | -0.0557 | 0.0117 | 0.00000197 | A | C | 0.000257 | 0.000612 | 0.67 | 6 | 55113411 | 22.66 |
| rs332823 | G | T | 0.0439 | 0.0093 | 0.00000255 | G | T | -0.000082 | 0.000486 | 0.87 | 1 | 61674052 | 22.28 |
| rs34557301 | C | T | -0.0865 | 0.0189 | 0.00000487 | C | T | 0.000286 | 0.000987 | 0.77 | 8 | 81215710 | 20.95 |
| rs34762282 | A | G | -0.0454 | 0.0093 | 0.00000116 | A | G | -0.000028 | 0.000487 | 0.95 | 1 | 110576662 | 23.83 |
| rs35003739 | A | T | 0.1442 | 0.0308 | 0.00000293 | A | T | -0.00161 | 0.001604 | 0.32 | 11 | 121240231 | 21.92 |
| rs35611929 | A | G | 0.0559 | 0.0098 | 1.21E-08 | A | G | 0.00009 | 0.000511 | 0.86 | 5 | 77467824 | 32.54 |
| rs35615188 | A | G | 0.1387 | 0.0298 | 0.00000322 | A | G | 0.001402 | 0.001551 | 0.37 | 4 | 8478002 | 21.66 |
| rs35996600 | A | G | 0.0494 | 0.0103 | 0.00000179 | A | G | -0.00048 | 0.000539 | 0.37 | 1 | 245743967 | 23.00 |
| rs36710 | G | A | -0.0512 | 0.0106 | 0.00000126 | G | A | 0.000619 | 0.00055 | 0.26 | 5 | 127559444 | 23.33 |
| rs3764002 | T | C | -0.0564 | 0.0106 | 9.15E-08 | T | C | 0.000172 | 0.000549 | 0.75 | 12 | 108618630 | 28.31 |
| rs3843661 | A | G | 0.1055 | 0.023 | 0.00000467 | A | G | -0.00108 | 0.001203 | 0.37 | 12 | 63108904 | 21.04 |
| rs3892354 | G | T | 0.0482 | 0.0094 | 0.000000332 | G | T | -0.000099 | 0.000491 | 0.84 | 9 | 4282942 | 26.29 |
| rs3958122 | T | C | 0.0504 | 0.0098 | 0.000000276 | T | C | 0.000012 | 0.000511 | 0.98 | 4 | 1693931 | 26.45 |
| rs4315502 | G | T | -0.0493 | 0.01 | 0.000000871 | G | T | 0.000551 | 0.000492 | 0.26 | 2 | 204831455 | 24.30 |
| rs4775006 | A | C | 0.0578 | 0.0094 | 8.4E-10 | A | C | 0.000361 | 0.00049 | 0.46 | 15 | 58215727 | 37.81 |
| rs4960263 | A | G | 0.0692 | 0.0141 | 0.000000889 | A | G | -0.00046 | 0.000734 | 0.53 | 6 | 6884733 | 24.09 |
| rs56116847 | A | G | 0.0612 | 0.0097 | 3.19E-10 | A | G | 0.000482 | 0.000507 | 0.34 | 12 | 123835233 | 39.81 |
| rs659437 | C | T | 0.0588 | 0.0126 | 0.00000327 | C | T | 0.000153 | 0.000658 | 0.82 | 1 | 46037394 | 21.78 |
| rs6940215 | A | G | 0.0539 | 0.0107 | 0.000000478 | A | G | 0.000489 | 0.000545 | 0.37 | 6 | 34764443 | 25.38 |
| rs7045125 | C | T | -0.0483 | 0.01 | 0.00000126 | C | T | 0.000363 | 0.000484 | 0.450001 | 9 | 95171852 | 23.33 |
| rs7141443 | A | G | 0.1193 | 0.0261 | 0.00000472 | A | G | -0.00114 | 0.001337 | 0.4 | 14 | 34964486 | 20.89 |
| rs7305875 | A | T | 0.0479 | 0.0095 | 0.000000429 | A | T | 0.000011 | 0.000493 | 0.98 | 12 | 23971243 | 25.42 |
| rs73093407 | A | C | 0.0815 | 0.0158 | 0.000000242 | A | C | 0.001403 | 0.000822 | 0.088 | 3 | 71512674 | 26.61 |
| rs731831 | A | G | 0.0466 | 0.0099 | 0.00000274 | A | G | -0.00092 | 0.000517 | 0.075999 | 3 | 52536514 | 22.16 |
| rs73230709 | G | C | 0.0589 | 0.0124 | 0.00000191 | G | C | -0.00103 | 0.000647 | 0.11 | 4 | 38514577 | 22.56 |
| rs751450 | G | A | -0.0459 | 0.0095 | 0.00000137 | G | A | 0.0000565 | 0.000495 | 0.91 | 10 | 73761015 | 23.34 |
| rs75621460 | A | G | 0.1433 | 0.0313 | 0.00000482 | A | G | 0.000357 | 0.001636 | 0.83 | 19 | 41833784 | 20.96 |
| rs75691457 | G | A | -0.0461 | 0.0099 | 0.00000302 | G | A | 0.000405 | 0.000485 | 0.4 | 17 | 42097956 | 21.68 |
| rs774299 | A | G | 0.0471 | 0.0096 | 0.000000962 | A | G | 0.0000477 | 0.000502 | 0.92 | 16 | 84808900 | 24.07 |
| rs78853750 | C | T | -0.1026 | 0.0224 | 0.00000474 | C | T | -0.001 | 0.001172 | 0.39 | 12 | 98356937 | 20.98 |
| rs7913051 | G | A | -0.0462 | 0.0095 | 0.00000102 | G | A | -0.00048 | 0.000493 | 0.33 | 10 | 130344003 | 23.65 |
| rs79359532 | G | T | 0.0705 | 0.013 | 6.23E-08 | G | T | -0.00138 | 0.000642 | 0.032 | 15 | 50735291 | 29.41 |
| rs8067763 | A | G | -0.0566 | 0.0095 | 2.39E-09 | A | G | 0.001205 | 0.000495 | 0.015 | 17 | 70012939 | 35.50 |
| rs8067895 | A | G | 0.0616 | 0.0103 | 1.89E-09 | A | G | 0.000647 | 0.000535 | 0.23 | 17 | 2030234 | 35.77 |
| rs8132883 | C | T | -0.0476 | 0.0101 | 0.00000275 | C | T | 0.0000481 | 0.00053 | 0.93 | 21 | 37601881 | 22.21 |
| rs9277552 | T | C | -0.064 | 0.0114 | 1.97E-08 | T | C | 0.000667 | 0.000592 | 0.26 | 6 | 33055501 | 31.52 |
| rs9357004 | C | T | -0.0481 | 0.0093 | 0.00000021 | C | T | -0.00036 | 0.000482 | 0.450001 | 6 | 26303319 | 26.75 |
| rs9557430 | A | G | 0.0557 | 0.012 | 0.0000037 | A | G | -0.00015 | 0.000629 | 0.82 | 13 | 101060995 | 21.55 |
| rs9890032 | G | C | -0.0519 | 0.0096 | 5.45E-08 | G | C | 0.000263 | 0.000497 | 0.6 | 17 | 29165934 | 29.23 |
| rs9950467 | C | T | 0.0544 | 0.0112 | 0.00000124 | C | T | 0.0000619 | 0.000556 | 0.91 | 18 | 57704377 | 23.59 |
|  |  |  |  |  |  |  |  |  |  |  |  |  |  |
| SNP | Effects on HOA | | | | | Effects on depression | | | | | Chr | Position | F |
|  | EA | OA | Beta | SE | p-val | EA | OA | Beta | SE | p-val |  |  | statistic |
| rs10492367 | T | G | 0.1518 | 0.0148 | 1.25E-24 | T | G | 0.000611 | 0.00062 | 0.32 | 12 | 28014970 | 105.20 |
| rs10513845 | C | T | 0.0677 | 0.0138 | 0.000000849 | C | T | -0.00138 | 0.000572 | 0.016 | 3 | 189746461 | 24.07 |
| rs10773083 | T | G | -0.0609 | 0.0124 | 0.000000863 | T | G | -0.00064 | 0.000516 | 0.22 | 12 | 124791650 | 24.12 |
| rs10831477 | G | T | -0.0721 | 0.015 | 0.00000164 | G | T | -0.00075 | 0.000621 | 0.23 | 11 | 95797111 | 23.10 |
| rs10896015 | A | G | -0.0782 | 0.0132 | 2.74E-09 | A | G | 0.000631 | 0.000544 | 0.25 | 11 | 65323725 | 35.10 |
| rs11059094 | T | C | 0.0759 | 0.0117 | 7.38E-11 | T | C | -0.000092 | 0.000483 | 0.85 | 12 | 122606837 | 42.08 |
| rs115740542 | C | T | 0.1263 | 0.0224 | 0.000000016 | C | T | -0.000098 | 0.000929 | 0.92 | 6 | 26123502 | 31.79 |
| rs11583641 | T | C | -0.0811 | 0.0131 | 5.57E-10 | T | C | 0.001056 | 0.000543 | 0.052 | 1 | 183906245 | 38.33 |
| rs12040949 | T | C | -0.0665 | 0.012 | 2.83E-08 | T | C | -0.00049 | 0.000497 | 0.32 | 1 | 150447462 | 30.71 |
| rs12141730 | A | G | 0.074 | 0.0157 | 0.0000023 | A | G | 0.0000274 | 0.000655 | 0.97 | 1 | 40809127 | 22.22 |
| rs12209223 | A | C | 0.1558 | 0.0191 | 3.88E-16 | A | C | -0.0005 | 0.000805 | 0.54 | 6 | 76164589 | 66.54 |
| rs12229894 | T | C | -0.0679 | 0.0146 | 0.00000348 | T | C | -0.000078 | 0.000562 | 0.89 | 12 | 64035480 | 21.63 |
| rs1228024 | A | C | -0.0606 | 0.0123 | 0.000000843 | A | C | -0.00123 | 0.000511 | 0.016 | 11 | 47951353 | 24.27 |
| rs1245535 | G | C | 0.0572 | 0.012 | 0.00000182 | G | C | -0.00019 | 0.000496 | 0.7 | 10 | 73789033 | 22.72 |
| rs12602666 | A | G | -0.0537 | 0.0117 | 0.00000413 | A | G | 0.0000945 | 0.000484 | 0.85 | 17 | 54850753 | 21.07 |
| rs12827555 | A | G | 0.0793 | 0.015 | 0.000000112 | A | G | -0.000081 | 0.000621 | 0.9 | 12 | 85621934 | 27.95 |
| rs13300602 | G | A | 0.0716 | 0.0119 | 1.65E-09 | G | A | 0.000146 | 0.000494 | 0.77 | 9 | 129412938 | 36.20 |
| rs140581193 | T | G | -0.1184 | 0.0225 | 0.000000132 | T | G | 0.00156 | 0.000935 | 0.094999 | 5 | 71960209 | 27.69 |
| rs1421085 | C | T | 0.0571 | 0.0119 | 0.00000147 | C | T | 0.001157 | 0.000492 | 0.019 | 16 | 53800954 | 23.02 |
| rs149319406 | C | T | -0.2244 | 0.0472 | 0.00000197 | C | T | 0.000516 | 0.001954 | 0.79 | 7 | 6393386 | 22.60 |
| rs1591421 | C | T | 0.0638 | 0.0121 | 0.00000013 | C | T | -0.0003 | 0.000501 | 0.55 | 9 | 117945427 | 27.80 |
| rs17677555 | C | G | 0.071 | 0.0132 | 7.91E-08 | C | G | -0.00056 | 0.00055 | 0.31 | 5 | 127852612 | 28.93 |
| rs1835323 | T | C | -0.0673 | 0.0123 | 4.56E-08 | T | C | 0.000643 | 0.00051 | 0.21 | 2 | 43512130 | 29.94 |
| rs1913707 | G | A | -0.0795 | 0.012 | 2.96E-11 | G | A | 0.000395 | 0.000496 | 0.43 | 4 | 13039440 | 43.89 |
| rs2396502 | C | A | 0.0842 | 0.012 | 2.12E-12 | C | A | 0.000785 | 0.000497 | 0.11 | 6 | 45357699 | 49.23 |
| rs2785988 | A | C | 0.0828 | 0.0127 | 7.3E-11 | A | C | 0.000723 | 0.000528 | 0.17 | 1 | 219744138 | 42.51 |
| rs2836618 | A | G | 0.0876 | 0.0132 | 3.2E-11 | A | G | 0.000505 | 0.000549 | 0.36 | 21 | 40048295 | 44.04 |
| rs2894875 | G | C | -0.1807 | 0.0376 | 0.00000151 | G | C | -0.000091 | 0.001535 | 0.95 | 5 | 7442527 | 23.10 |
| rs30152 | T | G | 0.0584 | 0.0126 | 0.00000353 | T | G | 0.000419 | 0.000524 | 0.42 | 16 | 14382647 | 21.48 |
| rs3774355 | A | G | 0.0907 | 0.0121 | 8.2E-14 | A | G | -0.00175 | 0.000504 | 0.00053 | 3 | 52817778 | 56.19 |
| rs4252548 | T | C | 0.2785 | 0.0396 | 1.96E-12 | T | C | -0.00068 | 0.001647 | 0.68 | 19 | 55879672 | 49.46 |
| rs4303288 | C | A | 0.0574 | 0.012 | 0.00000175 | C | A | -0.00078 | 0.000498 | 0.12 | 12 | 48336619 | 22.88 |
| rs4338381 | G | A | -0.095 | 0.0121 | 4.37E-15 | G | A | 0.000189 | 0.000502 | 0.709999 | 1 | 103572927 | 61.64 |
| rs4714874 | G | A | -0.0613 | 0.0118 | 0.0000002 | G | A | 0.000301 | 0.00049 | 0.54 | 6 | 45777475 | 26.99 |
| rs4837129 | A | G | 0.0703 | 0.0138 | 0.000000375 | A | G | -0.00117 | 0.000576 | 0.042 | 9 | 129848136 | 25.95 |
| rs62059839 | T | C | 0.0613 | 0.0133 | 0.00000386 | T | C | -0.00043 | 0.000552 | 0.44 | 17 | 7533015 | 21.24 |
| rs62063281 | G | A | 0.0964 | 0.014 | 5.3E-12 | G | A | 0.001019 | 0.000583 | 0.08 | 17 | 44038785 | 47.41 |
| rs66579515 | G | A | -0.0571 | 0.0124 | 0.00000417 | G | A | -0.00041 | 0.000516 | 0.43 | 16 | 83953384 | 21.20 |
| rs7222178 | A | T | 0.0965 | 0.0146 | 3.77E-11 | A | T | -0.00023 | 0.000605 | 0.7 | 17 | 59652282 | 43.69 |
| rs7294671 | G | A | 0.0569 | 0.0117 | 0.00000114 | G | A | 0.00049 | 0.000484 | 0.31 | 12 | 90344695 | 23.65 |
| rs73004362 | T | A | 0.0793 | 0.0153 | 0.000000221 | T | A | -0.00056 | 0.000638 | 0.38 | 2 | 242365353 | 26.86 |
| rs74767794 | G | A | -0.0751 | 0.0126 | 2.56E-09 | G | A | -0.00014 | 0.000523 | 0.79 | 1 | 184006128 | 35.53 |
| rs75621460 | A | G | 0.2007 | 0.0392 | 0.000000311 | A | G | 0.000357 | 0.001636 | 0.83 | 19 | 41833784 | 26.21 |
| rs75686861 | A | G | 0.0957 | 0.0203 | 0.00000234 | A | G | -0.00129 | 0.00084 | 0.12 | 4 | 145621328 | 22.22 |
| rs7571789 | C | T | -0.0886 | 0.0117 | 3.26E-14 | C | T | -0.00043 | 0.000484 | 0.38 | 2 | 70714793 | 57.35 |
| rs76573659 | C | T | 0.2056 | 0.0388 | 0.000000119 | C | T | 0.00233 | 0.001641 | 0.16 | 1 | 118690084 | 28.08 |
| rs7663743 | A | G | 0.0547 | 0.0117 | 0.00000298 | A | G | -0.000051 | 0.000485 | 0.92 | 4 | 13076120 | 21.86 |
| rs77069578 | G | A | 0.1894 | 0.0348 | 5.43E-08 | G | A | 0.000554 | 0.001442 | 0.7 | 7 | 18607826 | 29.62 |
| rs771372 | A | T | 0.084 | 0.0175 | 0.00000161 | A | T | 0.00047 | 0.000729 | 0.52 | 11 | 10880371 | 23.04 |
| rs7809623 | T | C | -0.0605 | 0.0125 | 0.00000131 | T | C | -0.00071 | 0.000519 | 0.17 | 7 | 50805499 | 23.43 |
| rs78903563 | T | C | 0.143 | 0.0295 | 0.00000122 | T | C | -0.00043 | 0.001127 | 0.709999 | 6 | 41616445 | 23.50 |
| rs79056043 | G | A | 0.1625 | 0.0268 | 1.33E-09 | G | A | 0.001156 | 0.001133 | 0.31 | 12 | 59289598 | 36.77 |
| rs79164994 | G | A | -0.0878 | 0.0152 | 8.62E-09 | G | A | -0.00111 | 0.000599 | 0.063 | 8 | 130725665 | 33.37 |
| rs79560566 | A | G | 0.0966 | 0.0203 | 0.00000208 | A | G | 0.000798 | 0.000567 | 0.16 | 3 | 170641587 | 22.64 |
| rs79752115 | A | C | -0.0927 | 0.0178 | 0.00000018 | A | C | -0.00066 | 0.000737 | 0.37 | 9 | 110428477 | 27.12 |
| rs798748 | C | T | 0.0715 | 0.012 | 2.5E-09 | C | T | -0.000025 | 0.000497 | 0.96 | 4 | 1716770 | 35.50 |
| rs80287694 | G | A | 0.1093 | 0.0184 | 2.66E-09 | G | A | -0.00024 | 0.000759 | 0.75 | 6 | 55636940 | 35.29 |
| rs8056012 | G | A | -0.0596 | 0.0118 | 0.000000439 | G | A | 0.000106 | 0.00049 | 0.83 | 16 | 87184318 | 25.51 |
| rs931698 | A | C | 0.064 | 0.0137 | 0.00000294 | A | C | -0.000079 | 0.00057 | 0.89 | 3 | 55540870 | 21.82 |
|  |  |  |  |  |  |  |  |  |  |  |  |  |  |
|  |  |  |  |  |  |  |  |  |  |  |  |  |  |
|  |  |  |  |  |  |  |  |  |  |  |  |  |  |
| SNP | Effects on Spondyloarthritis | | | | | Effects on depression | | | | | Chr | Position | F |
|  | EA | OA | Beta | SE | p-val | EA | OA | Beta | SE | p-val |  |  | statistic |
| rs10807943 | C | T | -0.3449 | 0.057 | 1.49E-09 | C | T | 0.000255 | 0.000775 | 0.74 | 7 | 5340664 | 36.61 |
| rs115446347 | C | T | 1.9146 | 0.0513 | 1E-200 | C | T | -0.00103 | 0.001028 | 0.32 | 6 | 31301732 | 1392.90 |
| rs11751487 | T | C | 1.1175 | 0.0563 | 1.31E-87 | T | C | -0.0012 | 0.001261 | 0.34 | 6 | 28329625 | 393.98 |
| rs13015130 | T | C | -0.1516 | 0.0318 | 0.00000193 | T | C | 0.000446 | 0.000577 | 0.44 | 2 | 114034952 | 22.73 |
| rs1368900 | G | A | 0.1238 | 0.027 | 0.00000449 | G | A | 0.000182 | 0.00049 | 0.709999 | 2 | 80734826 | 21.02 |
| rs2032890 | C | A | -0.1479 | 0.0303 | 0.00000108 | C | A | -0.00069 | 0.000523 | 0.19 | 5 | 96121152 | 23.83 |
| rs241426 | A | T | -0.2334 | 0.0277 | 3.99E-17 | A | T | -0.00103 | 0.000497 | 0.037 | 6 | 32804553 | 71.00 |
| rs28418842 | A | C | 0.3684 | 0.0739 | 0.000000623 | A | C | -0.00033 | 0.001001 | 0.75 | 16 | 78654343 | 24.85 |
| rs60556766 | T | C | 0.1887 | 0.0397 | 0.00000196 | T | C | 0.000771 | 0.000673 | 0.25 | 7 | 4934453 | 22.59 |
| rs6924600 | A | G | 0.4757 | 0.0319 | 3.24E-50 | A | G | 0.000254 | 0.000641 | 0.69 | 6 | 30857542 | 222.37 |
| rs715863 | G | A | -0.1259 | 0.0271 | 0.00000333 | G | A | -0.00017 | 0.000503 | 0.73 | 20 | 24525848 | 21.58 |
| rs72828977 | T | C | 0.379 | 0.0554 | 7.77E-12 | T | C | 0.001597 | 0.001139 | 0.16 | 6 | 24211368 | 46.80 |
| rs73992454 | T | C | 0.4555 | 0.0849 | 8.21E-08 | T | C | -0.00045 | 0.001763 | 0.8 | 2 | 225183298 | 28.78 |
| rs7762253 | T | C | 0.3824 | 0.0275 | 7.62E-44 | T | C | -0.00096 | 0.000486 | 0.048 | 6 | 29676823 | 193.36 |
| rs78791153 | C | G | 0.363 | 0.0772 | 0.00000254 | C | G | -0.00042 | 0.001202 | 0.73 | 4 | 113976450 | 22.11 |
| rs821596 | T | C | 0.1334 | 0.0286 | 0.00000315 | T | C | 0.001002 | 0.00053 | 0.059 | 1 | 232101598 | 21.76 |
| rs999942 | C | G | 0.278 | 0.029 | 7.95E-22 | C | G | -0.00031 | 0.000581 | 0.59 | 6 | 33624902 | 91.90 |
|  |  |  |  |  |  |  |  |  |  |  |  |  |  |
| SNP | Effects on AS | | | | | Effects on depression | | | | | Chr | Position | F |
|  | EA | OA | Beta | SE | p-val | EA | OA | Beta | SE | p-val |  |  | statistic |
| rs1015568 | G | A | -0.00056 | 0.000111 | 0.00000047 | G | A | 0.0000918 | 0.000487 | 0.85 | 6 | 16432284 | 25.45 |
| rs1038888 | G | T | 0.000515 | 0.00011 | 0.000003 | G | T | 0.000334 | 0.000485 | 0.49 | 16 | 62660689 | 21.92 |
| rs12145316 | T | C | -0.00054 | 0.000111 | 0.0000013 | T | C | -0.00085 | 0.000487 | 0.081001 | 1 | 30245413 | 23.67 |
| rs2395143 | G | C | 0.000938 | 0.00012 | 4.8E-15 | G | C | 0.000574 | 0.000527 | 0.28 | 6 | 32293578 | 61.10 |
| rs2523586 | G | T | 0.002571 | 0.000123 | 1.2E-97 | G | T | 0.000465 | 0.000539 | 0.39 | 6 | 31327435 | 436.91 |
| rs2836902 | T | G | 0.000519 | 0.00011 | 0.0000024 | T | G | 0.000673 | 0.000485 | 0.16 | 21 | 40499467 | 22.26 |
| rs3749939 | C | T | 0.002792 | 0.000124 | 4.6E-113 | C | T | 0.000543 | 0.000544 | 0.32 | 6 | 31407247 | 506.98 |
| rs9266639 | T | C | 0.00272 | 0.000122 | 7.4E-110 | T | C | -0.0014 | 0.000537 | 0.0094 | 6 | 31347069 | 497.07 |
| rs9405064 | C | A | 0.000933 | 0.000139 | 1.7E-11 | C | A | 0.000283 | 0.000609 | 0.64 | 6 | 31292582 | 45.05 |
|  |  |  |  |  |  |  |  |  |  |  |  |  |  |
| SNP | Effects on Seronegative RA | | | | | Effects on depression | | | | | Chr | Position | F |
|  | EA | OA | Beta | SE | p-val | EA | OA | Beta | SE | p-val |  |  | statistic |
| rs142678586 | A | G | 2.0712 | 0.4473 | 0.00000365 | A | G | 0.001453 | 0.001988 | 0.46 | 3 | 6804479 | 21.44 |
| rs145575084 | C | G | 0.873 | 0.0682 | 1.49E-37 | C | G | -0.00131 | 0.001201 | 0.27 | 6 | 31365089 | 163.86 |
| rs17120968 | T | C | 0.5819 | 0.1223 | 0.00000194 | T | C | -0.00203 | 0.001678 | 0.23 | 11 | 117493839 | 22.64 |
| rs2476601 | G | A | -0.3212 | 0.0472 | 9.96E-12 | G | A | -0.00207 | 0.000801 | 0.0099 | 1 | 114377568 | 46.31 |
| rs304729 | A | G | 0.1996 | 0.043 | 0.00000346 | A | G | 0.001161 | 0.000618 | 0.06 | 19 | 44084403 | 21.55 |
| rs34057012 | A | G | -0.1572 | 0.0336 | 0.00000285 | A | G | -0.00089 | 0.000505 | 0.077 | 16 | 89743083 | 21.89 |
| rs4660553 | A | G | 0.2971 | 0.0649 | 0.00000469 | A | G | 0.00111 | 0.001223 | 0.36 | 1 | 42134604 | 20.96 |
| rs4902632 | T | A | -0.1797 | 0.0389 | 0.00000378 | T | A | 0.000277 | 0.000648 | 0.67 | 14 | 69149428 | 21.34 |
| rs5761256 | G | A | 0.1753 | 0.0364 | 0.00000145 | G | A | -0.00029 | 0.000537 | 0.59 | 22 | 26218164 | 23.19 |
| rs72933309 | T | C | -0.3687 | 0.0807 | 0.00000489 | T | C | 0.001161 | 0.001933 | 0.55 | 6 | 109194634 | 20.87 |
| rs74483355 | T | C | 0.3235 | 0.068 | 0.00000198 | T | C | -0.00027 | 0.000765 | 0.719999 | 16 | 30836430 | 22.63 |
| rs75132258 | T | C | 0.3641 | 0.0774 | 0.00000255 | T | C | 0.000107 | 0.001004 | 0.92 | 10 | 126812002 | 22.13 |
| rs75351188 | T | G | -0.3515 | 0.076 | 0.00000371 | T | G | 0.002693 | 0.001046 | 0.01 | 12 | 117005064 | 21.39 |
| rs75549101 | C | G | 0.7694 | 0.1662 | 0.00000366 | C | G | 0.002638 | 0.001372 | 0.054 | 4 | 11910875 | 21.43 |
| rs7731626 | A | G | -0.1743 | 0.0369 | 0.00000235 | A | G | 0.000133 | 0.000499 | 0.79 | 5 | 55444683 | 22.31 |
| rs78502259 | A | G | 0.4986 | 0.109 | 0.00000479 | A | G | -0.0026 | 0.001343 | 0.052 | 5 | 138752403 | 20.92 |
| rs78525147 | A | C | 0.5994 | 0.1268 | 0.00000226 | A | C | 0.001032 | 0.001523 | 0.5 | 3 | 115614340 | 22.35 |
| rs9296004 | C | A | 0.3251 | 0.0566 | 9.39E-09 | C | A | -0.00024 | 0.000886 | 0.780001 | 6 | 31933977 | 32.99 |
|  |  |  |  |  |  |  |  |  |  |  |  |  |  |
| SNP | Effects on Seropositive RA | | | | | Effects on depression | | | | | Chr | Position | F |
|  | EA | OA | Beta | SE | p-val | EA | OA | Beta | SE | p-val |  |  | statistic |
| rs13135106 | C | T | 0.3245 | 0.07 | 0.00000353 | C | T | -0.0007 | 0.000792 | 0.38 | 4 | 185812423 | 21.49 |
| rs149696396 | A | G | 0.5908 | 0.1262 | 0.00000286 | A | G | -0.00082 | 0.002275 | 0.719999 | 14 | 99934133 | 21.92 |
| rs2517929 | G | A | 0.2035 | 0.0426 | 0.00000182 | G | A | -0.00049 | 0.000521 | 0.34 | 6 | 29745175 | 22.82 |
| rs2524084 | G | A | 0.3771 | 0.0443 | 1.63E-17 | G | A | -0.0013 | 0.000505 | 0.0099 | 6 | 31241639 | 72.46 |
| rs2856822 | C | A | -0.4896 | 0.0504 | 2.45E-22 | C | A | 0.000475 | 0.000522 | 0.36 | 6 | 33047432 | 94.37 |
| rs62149098 | A | G | 0.3983 | 0.0849 | 0.00000272 | A | G | 0.00017 | 0.000845 | 0.84 | 2 | 36044575 | 22.01 |
| rs62318061 | A | G | 1.2431 | 0.2716 | 0.0000047 | A | G | -0.00003 | 0.001325 | 0.98 | 4 | 133293637 | 20.95 |
| rs62402721 | T | C | -0.5488 | 0.1171 | 0.00000277 | T | C | -0.0022 | 0.001649 | 0.18 | 6 | 32087258 | 21.96 |
| rs6448434 | A | G | 0.2002 | 0.0434 | 0.000004 | A | G | -0.00081 | 0.000522 | 0.12 | 4 | 26110113 | 21.28 |
| rs6923060 | A | G | -0.1959 | 0.0404 | 0.00000127 | A | G | -0.0003 | 0.000486 | 0.54 | 6 | 18506050 | 23.51 |
| rs78979751 | C | T | -0.2776 | 0.0604 | 0.00000436 | C | T | -0.00188 | 0.000927 | 0.043 | 19 | 45685068 | 21.12 |
| rs9544907 | T | C | 0.3582 | 0.0769 | 0.00000316 | T | C | -0.0003 | 0.000777 | 0.7 | 13 | 79450814 | 21.70 |
| rs9558538 | T | C | -0.2062 | 0.0425 | 0.00000122 | T | C | 0.00027 | 0.000541 | 0.62 | 13 | 106007838 | 23.54 |
|  |  |  |  |  |  |  |  |  |  |  |  |  |  |
| SNP | Effects on Pyogenic arthritis | | | | | Effects on depression | | | | | Chr | Position | F |
|  | EA | OA | Beta | SE | p-val | EA | OA | Beta | SE | p-val |  |  | statistic |
| rs149080440 | G | A | 1.6171 | 0.3347 | 0.00000135 | G | A | -0.0003 | 0.002167 | 0.89 | 8 | 122309077 | 23.34 |
| rs324459 | A | T | -0.2209 | 0.0454 | 0.00000113 | A | T | -0.00032 | 0.000518 | 0.54 | 9 | 9051277 | 23.67 |
| rs684795 | C | T | 0.4009 | 0.0876 | 0.00000469 | C | T | -0.00018 | 0.001169 | 0.87 | 9 | 90672032 | 20.94 |
| rs7141387 | C | A | 0.2125 | 0.0437 | 0.00000117 | C | A | 0.000325 | 0.000488 | 0.51 | 14 | 33070125 | 23.65 |
| rs72502580 | T | A | 0.5758 | 0.0762 | 4.18E-14 | T | A | -0.00045 | 0.000978 | 0.64 | 6 | 31367052 | 57.10 |
| rs73044104 | G | A | 0.6137 | 0.1253 | 0.000000959 | G | A | -0.00000091 | 0.001106 | 1 | 19 | 53723346 | 23.99 |
|  |  |  |  |  |  |  |  |  |  |  |  |  |  |
| SNP | Effects on gout | | | | | Effects on depression | | | | | Chr | Position | F |
|  | EA | OA | Beta | SE | p-val | EA | OA | Beta | SE | p-val |  |  | statistic |
| rs1064257 | G | C | 1.0269 | 0.0913 | 2.55E-29 | G | C | -0.00183 | 0.001087 | 0.092001 | 19 | 49993535 | 126.51 |
| rs112969496 | A | G | 2.0286 | 0.4414 | 0.00000432 | A | G | -0.000017 | 0.002007 | 0.99 | 22 | 38801610 | 21.12 |
| rs114266618 | A | T | 0.6065 | 0.1318 | 0.00000421 | A | T | 0.001055 | 0.001938 | 0.59 | 4 | 20532307 | 21.18 |
| rs118159268 | A | G | 0.5239 | 0.1074 | 0.00000107 | A | G | 0.000881 | 0.001498 | 0.56 | 15 | 76826557 | 23.80 |
| rs12435058 | C | T | 0.1668 | 0.0327 | 0.000000348 | C | T | -0.00094 | 0.000652 | 0.15 | 14 | 106291182 | 26.02 |
| rs1260326 | C | T | -0.1366 | 0.0265 | 0.000000248 | C | T | -0.00046 | 0.000493 | 0.35 | 2 | 27730940 | 26.57 |
| rs149136965 | C | G | 0.3958 | 0.0711 | 2.62E-08 | C | G | 0.002477 | 0.002119 | 0.24 | 6 | 30958409 | 30.99 |
| rs17884118 | C | T | 0.4655 | 0.1011 | 0.0000041 | C | T | 0.001368 | 0.001623 | 0.4 | 6 | 53385961 | 21.20 |
| rs239504 | G | A | -0.2085 | 0.0437 | 0.00000185 | G | A | 0.000612 | 0.000869 | 0.48 | 6 | 80685699 | 22.76 |
| rs2581792 | C | T | 0.13 | 0.0279 | 0.00000316 | C | T | 0.00089 | 0.000534 | 0.096 | 3 | 53035044 | 21.71 |
| rs2616133 | A | C | -0.1212 | 0.0259 | 0.00000273 | A | C | 0.000551 | 0.000487 | 0.26 | 10 | 71470529 | 21.90 |
| rs34004016 | C | T | -0.161 | 0.0278 | 7.06E-09 | C | T | 0.0000974 | 0.000567 | 0.86 | 4 | 9697965 | 33.54 |
| rs3747207 | A | G | -0.1595 | 0.0303 | 0.000000145 | A | G | -0.00061 | 0.000588 | 0.3 | 22 | 44324855 | 27.71 |
| rs3775948 | C | G | 0.3616 | 0.0302 | 4.81E-33 | C | G | -0.0006 | 0.000561 | 0.28 | 4 | 9995182 | 143.36 |
| rs45499402 | C | G | 0.7005 | 0.0509 | 5.03E-43 | C | G | 0.0004 | 0.000761 | 0.6 | 4 | 89043634 | 189.40 |
| rs4645896 | A | G | 0.4085 | 0.0655 | 4.48E-10 | A | G | 0.0000434 | 0.000981 | 0.96 | 19 | 49461834 | 38.90 |
| rs4861739 | A | G | -0.1155 | 0.0252 | 0.00000455 | A | G | -0.00022 | 0.000496 | 0.649999 | 4 | 188000355 | 21.01 |
| rs72791484 | A | C | 0.2059 | 0.0449 | 0.00000456 | A | C | -0.00056 | 0.000763 | 0.47 | 5 | 151801582 | 21.03 |
| rs75653560 | A | G | 0.5786 | 0.1198 | 0.00000136 | A | G | 0.003582 | 0.002098 | 0.088 | 17 | 51771005 | 23.33 |
| rs75858678 | T | C | 0.3208 | 0.066 | 0.00000118 | T | C | 0.002415 | 0.002033 | 0.23 | 2 | 8423440 | 23.63 |
| rs7759330 | G | A | -0.133 | 0.0285 | 0.00000298 | G | A | -0.00109 | 0.000547 | 0.046 | 6 | 7011934 | 21.78 |
| rs857499 | A | G | -0.1983 | 0.0414 | 0.00000165 | A | G | 0.000351 | 0.000756 | 0.64 | 17 | 70520808 | 22.94 |

OA：osteoarthritis;KOA：knee osteoarthritis;HOA：hip osteoarthritis;AS：ankylosing spondylitis;Seronegative RA：Seronegative rheumatoid arthritis;Seropositive RA：Seropositive rheumatoid arthritis;MR-PRESSO：MR pleiotropy residual sum and outlier;SNP：single-nucleotide polymorphism.
